# Supplementary material for: 3′,4′-Dihydro-2′H-spiro[indoline-3,1′-isoquinolin]-2-ones as potential anti-cancer agents: synthesis and preliminary screening
Source: R Soc Open Sci. 2020 Jan 8;7(1):191316. doi: 10.1098/rsos.191316 (PMC7029914; doi:10.1098/rsos.191316)
Supplement: Supplementarty Material: Spectral and Biological Data [file rsos191316supp1.docx]

**SUPPORTING INFORMATION**

3’,4’-Dihydro-2’H-spiro[indolin-3:1’-isoquinolin]-2-ones as Potential Anticancer Agents: Synthesis and Preliminary Screening.

Maloba M. M. Lobe^1^ and Simon M. N. Efange^1^*

^1^Department of Chemistry, University of Buea, P.O. Box 63, Buea, Cameroon.

*Corresponding author: Simon M.N. Efange, University of Buea, P.O. Box 63, Buea, Cameroon.

*Email: efange.mbua@ubuea.cm*

**Table of Contents page no.**

**^1^H NMR and ^13^C NMR spectra of target compounds S 2 - S81**

**LC-MS Spectra of target compounds S82 – S121**

**Biological Screening Data S122 –S127**


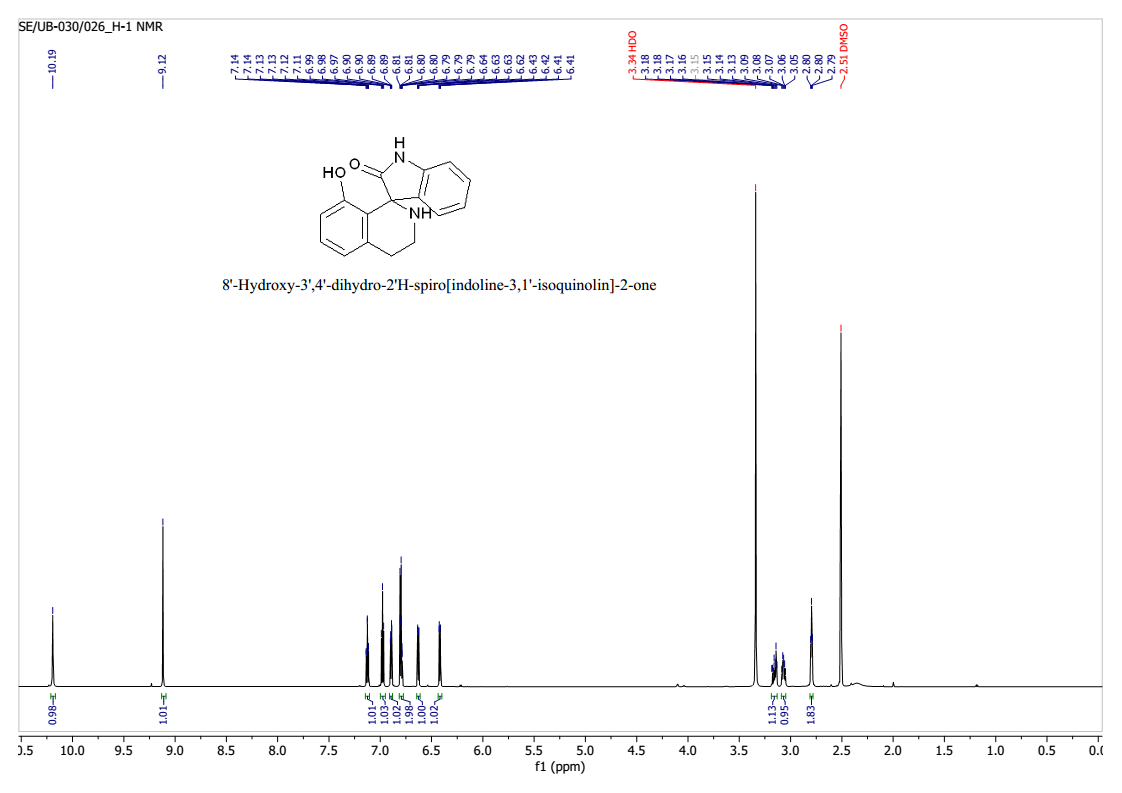
**^1^H NMR** **of** **8'-Hydroxy-3',4'-dihydro-2'H-spiro[indoline-3,1'-isoquinolin]-2-one (1a)**


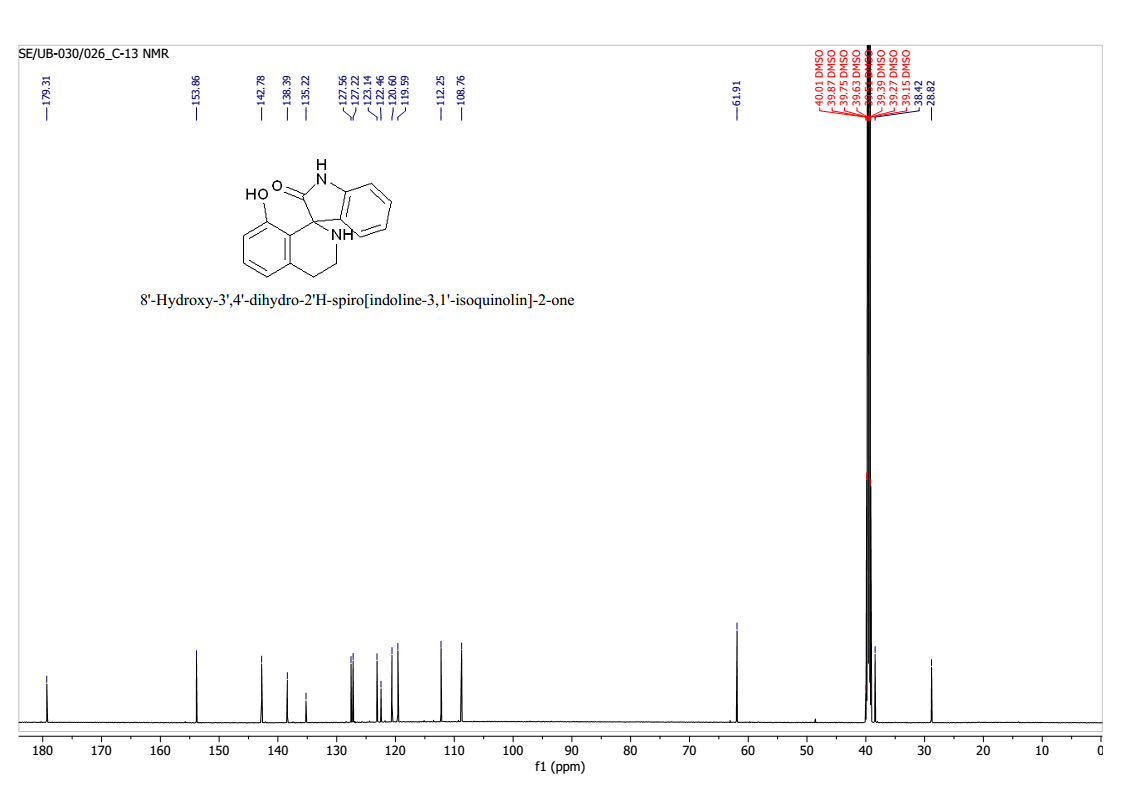
**^13^C NMR of 8'-Hydroxy-3',4'-dihydro-2'H-spiro[indoline-3,1'-isoquinolin]-2-one (1a)**


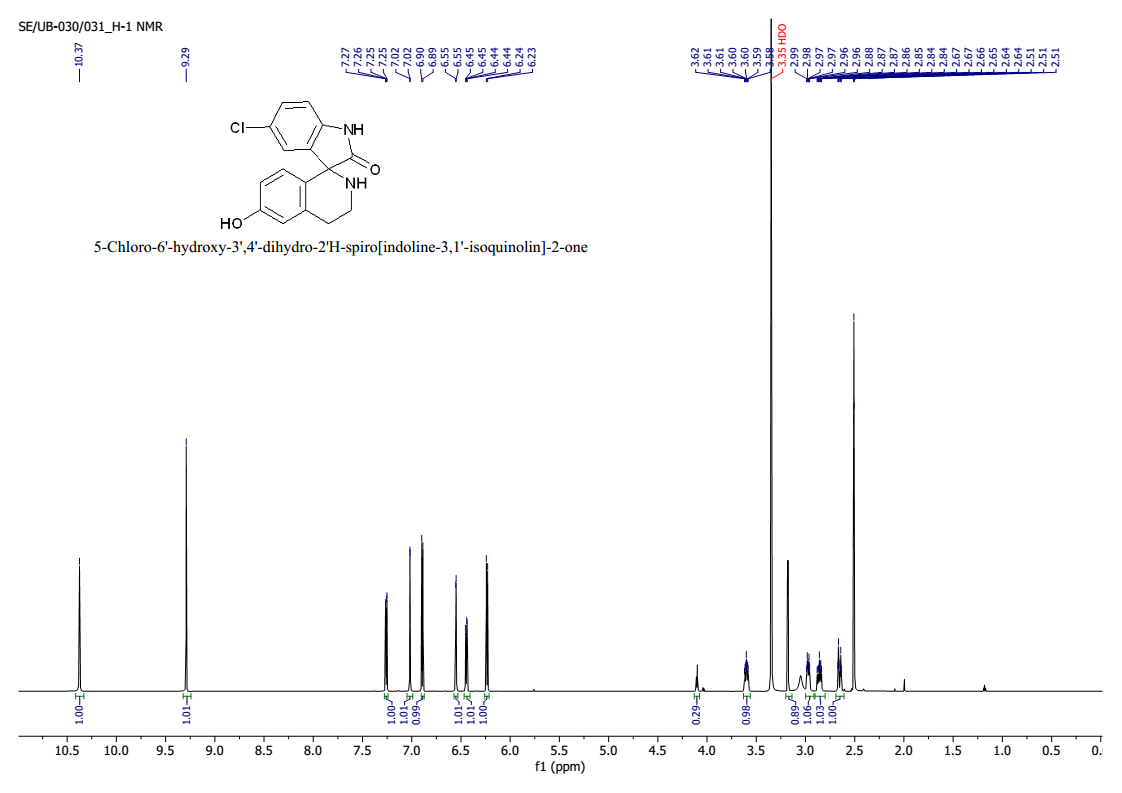
**^1^H NMR** **of** **5-Chloro-6'-hydroxy-3',4'-dihydro-2'H-spiro[indoline-3,1'-isoquinolin]-2-one (2b)**


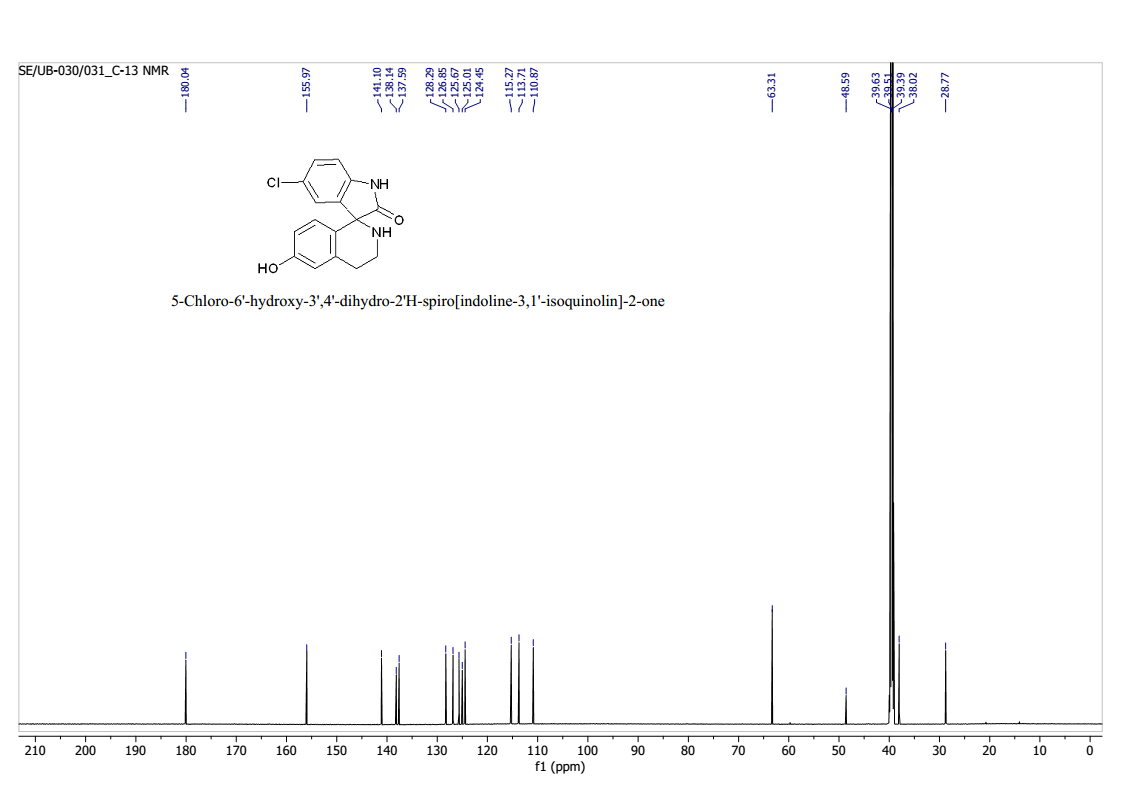
**^13^C NMR of 5-Chloro-6'-hydroxy-3',4'-dihydro-2'H-spiro[indoline-3,1'-isoquinolin]-2-one (2b)**


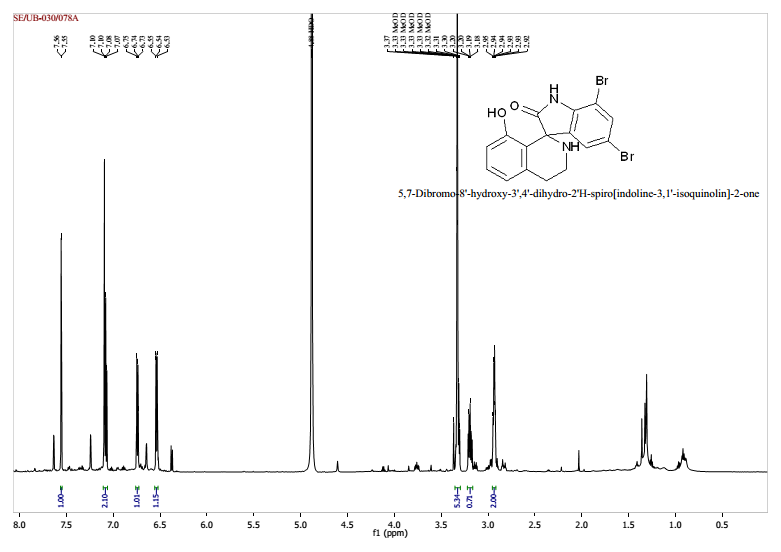
**^1^H NMR** **of**  **5,7-** **Dibromo-8'-hydroxy-3',4'-dihydro-2'H-spiro[indoline-3,1'-isoquinolin]-2-one (1c)**


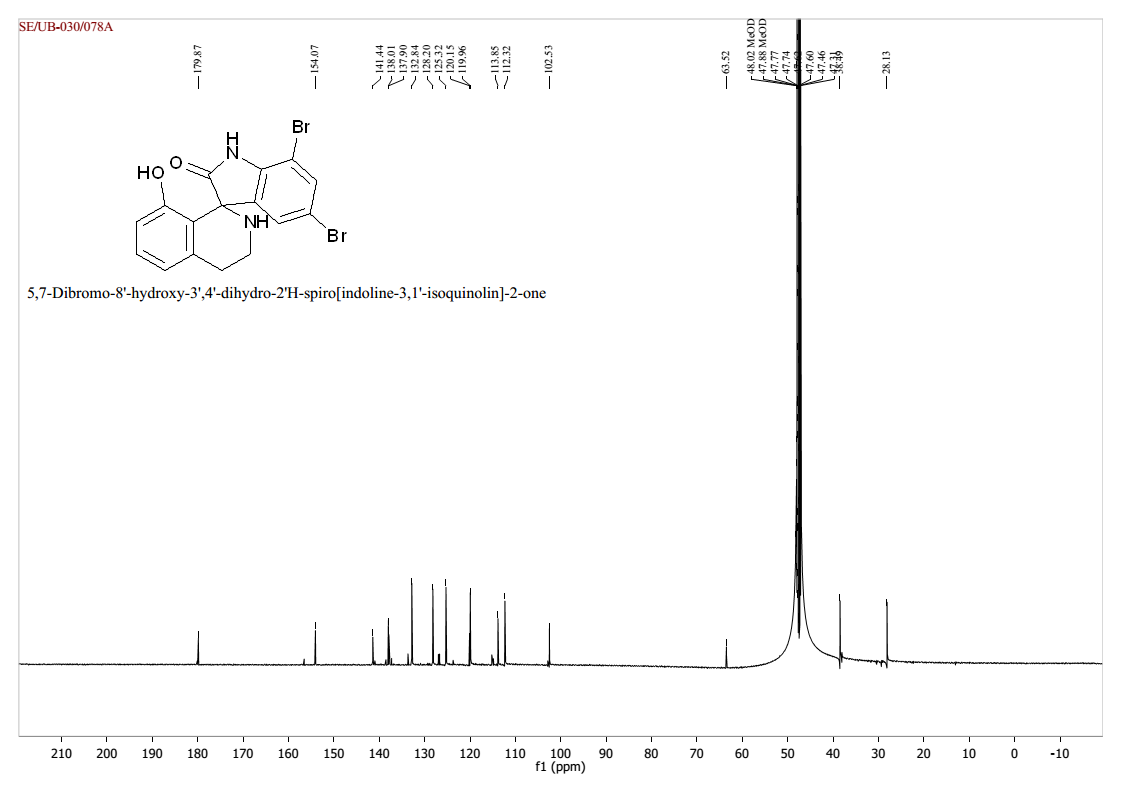
**^13^C NMR of 5,7-Dibromo-8'-hydroxy-3',4'-dihydro-2'H-spiro[indoline-3,1'-isoquinolin]-2-one (1c)**

**
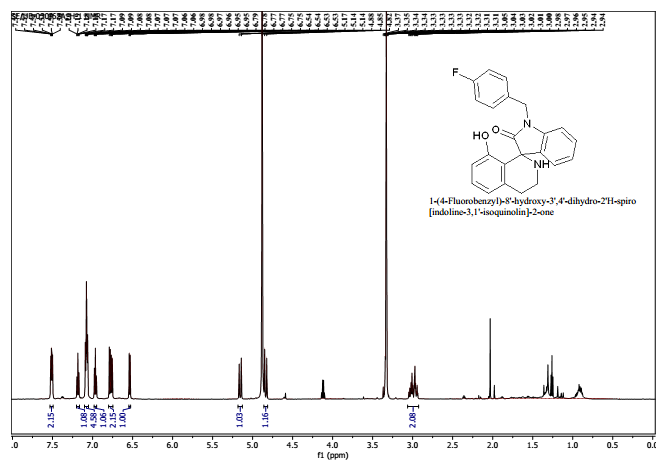
^1^H NMR** **of** **1-(4-Fluorobenzyl)-8'-hydroxy-3',4'-dihydro-2'H-spiro[indoline-3,1'-isoquinolin]-2-one (1d)**

**
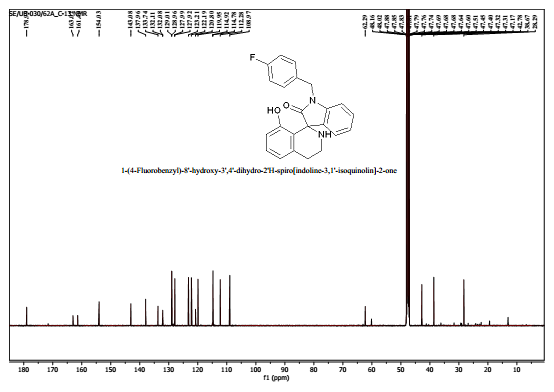
1-^13^C NMR of (4-Fluorobenzyl)-8'-hydroxy-3',4'-dihydro-2'H-spiro[indoline-3,1'-isoquinolin]-2-one (1d)**

**
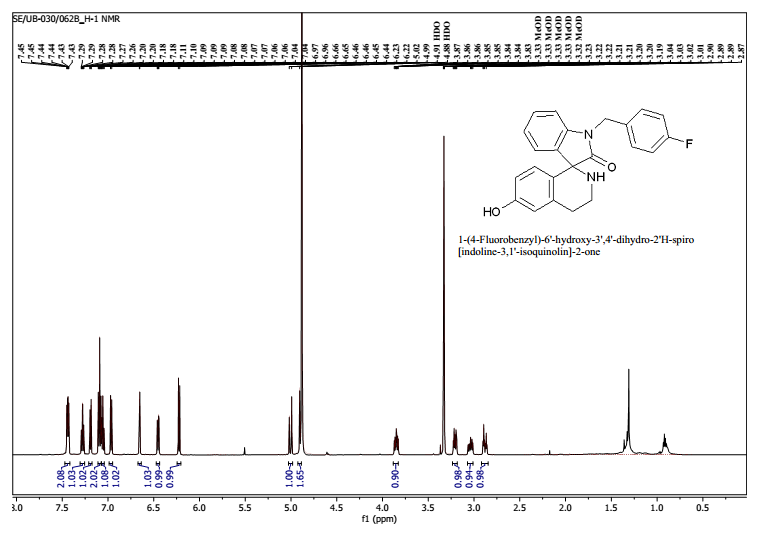
^1^H NMR** **of** **1-(4-Fluorobenzyl)-6'-hydroxy-3',4'-dihydro-2'H-spiro[indoline-3,1'-isoquinolin]-2-one (2d)**


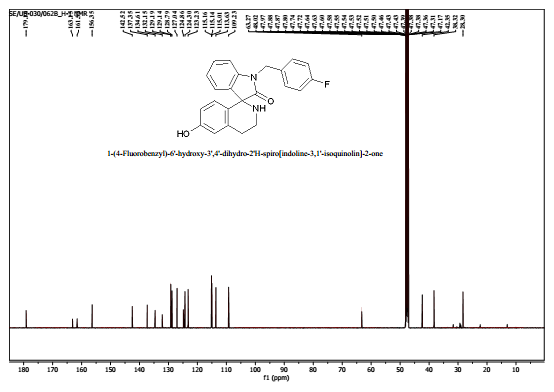
**1-^13^C NMR of (4-Fluorobenzyl)-6'-hydroxy-3',4'-dihydro-2'H-spiro[indoline-3,1'-isoquinolin]-2-one (2d)**

**
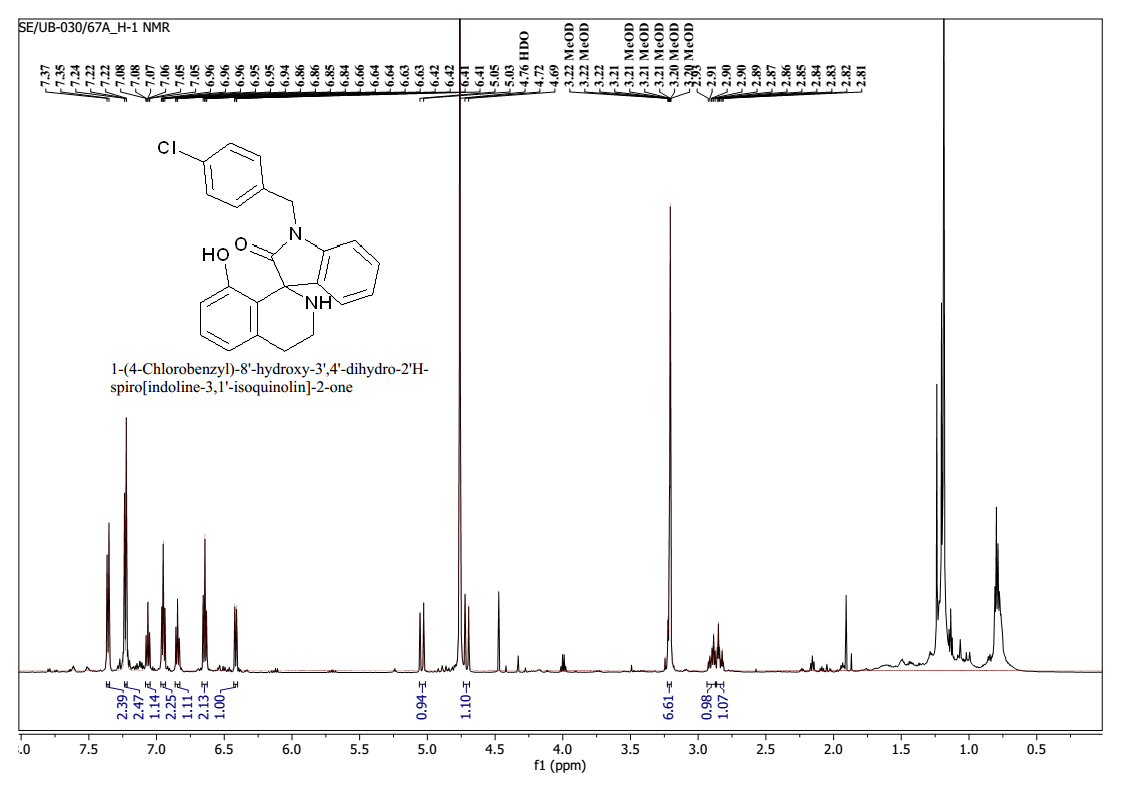
^1^H NMR** **of** **1-(4-Chlorobenzyl)-8'-hydroxy-3',4'-dihydro-2'H-spiro[indoline-3,1'-isoquinolin]-2-one (1e)**

**
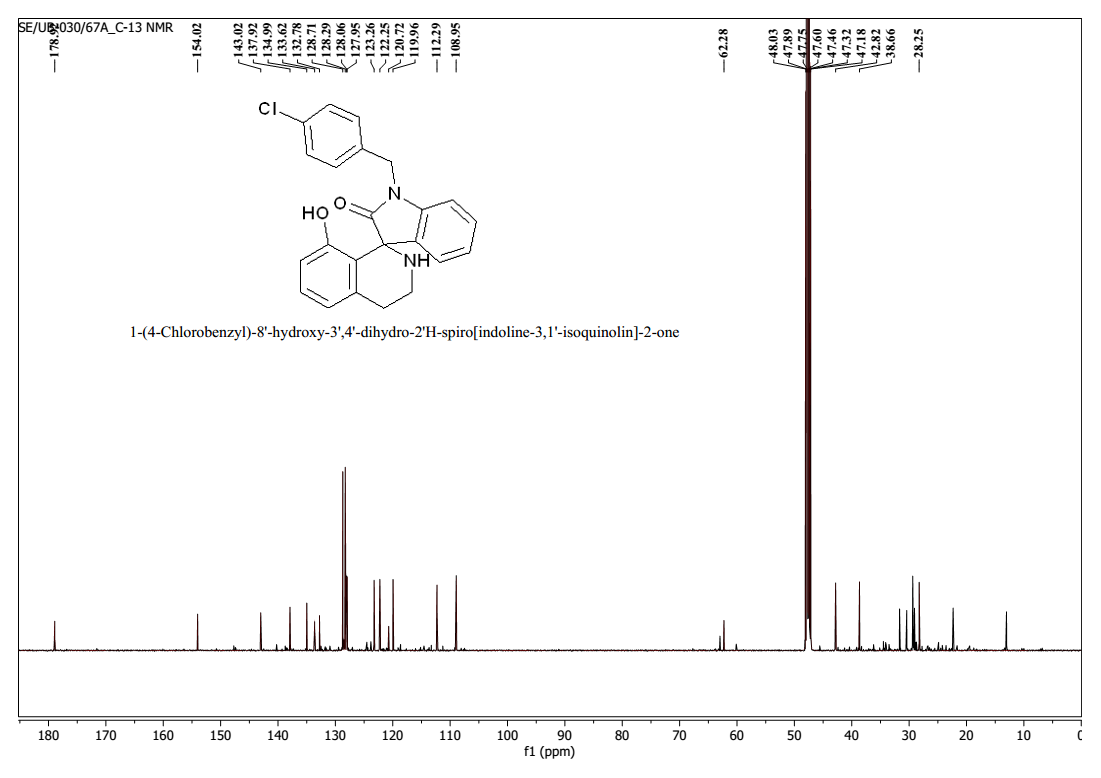
^13^C NMR of 1-(4-Chlorobenzyl)-8'-hydroxy-3',4'-dihydro-2'H-spiro[indoline-3,1'-isoquinolin]-2-one (1e)**

**
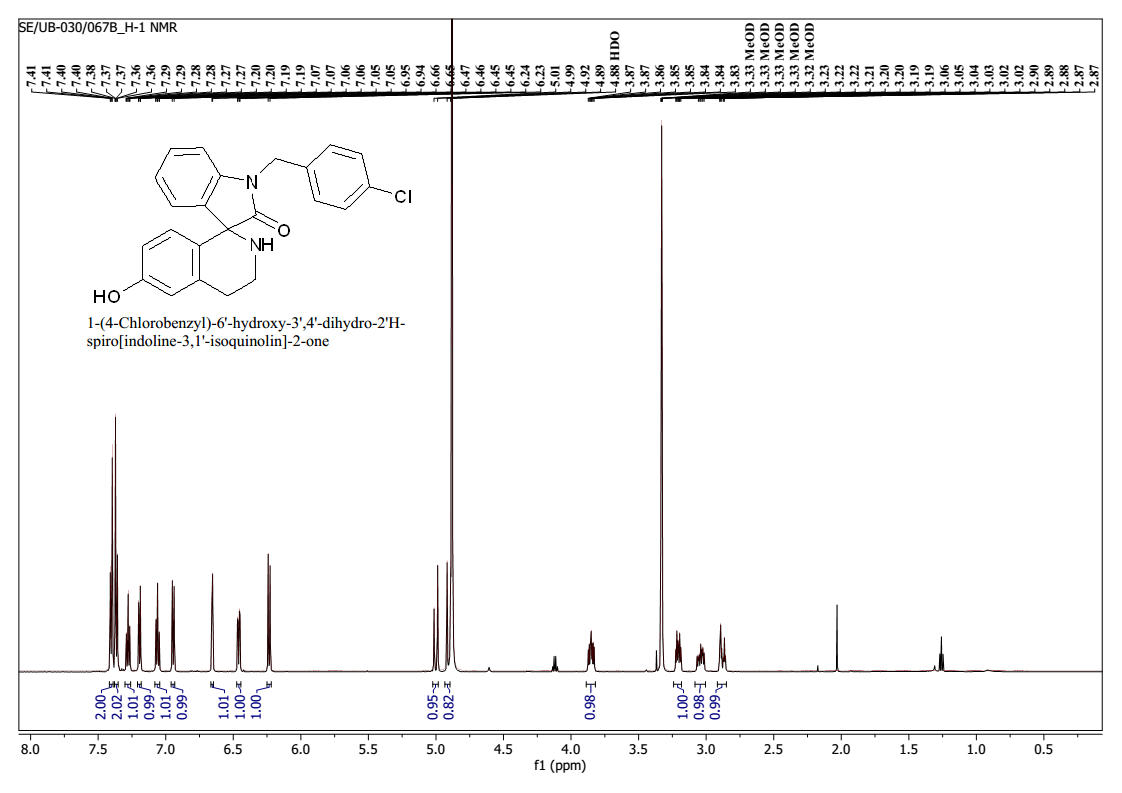
^1^H NMR** **of** **1-(4-Chlorobenzyl)-6'-hydroxy-3',4'-dihydro-2'H-spiro[indoline-3,1'-isoquinolin]-2-one (2e)**

**
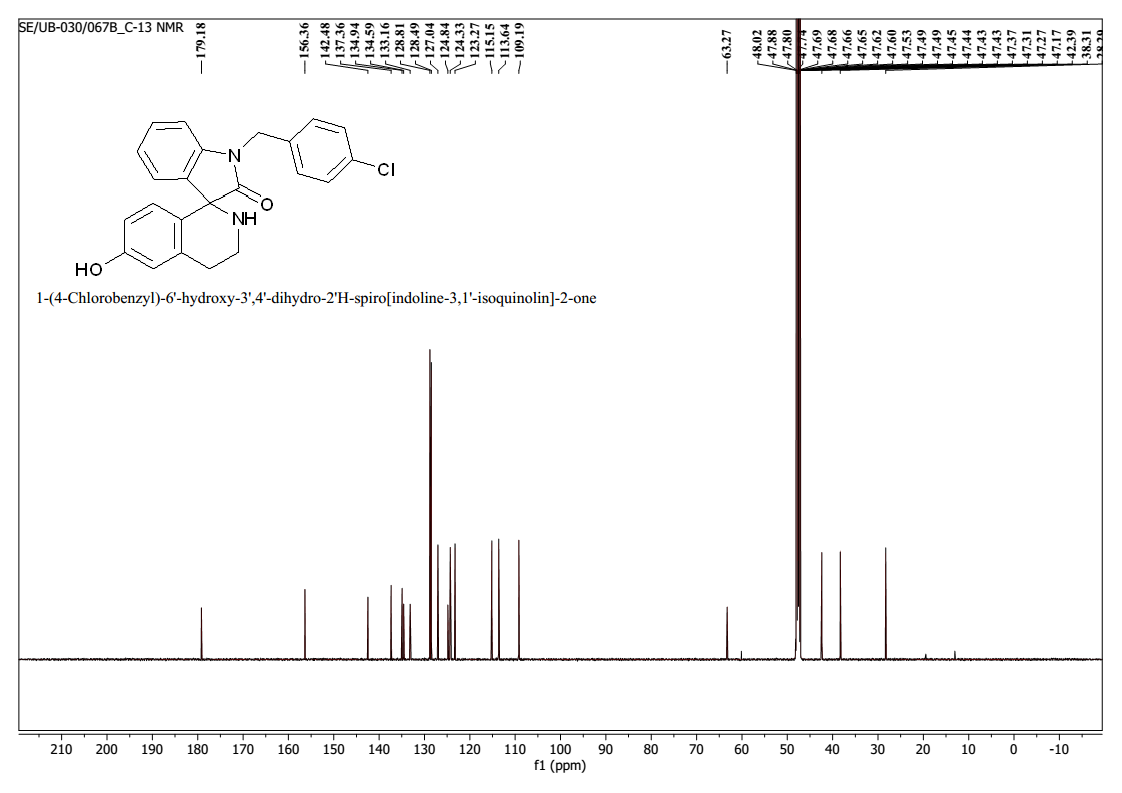
^13^C NMR of 1-(4-Chlorobenzyl)-6'-hydroxy-3',4'-dihydro-2'H-spiro[indoline-3,1'-isoquinolin]-2-one (2e)**

**
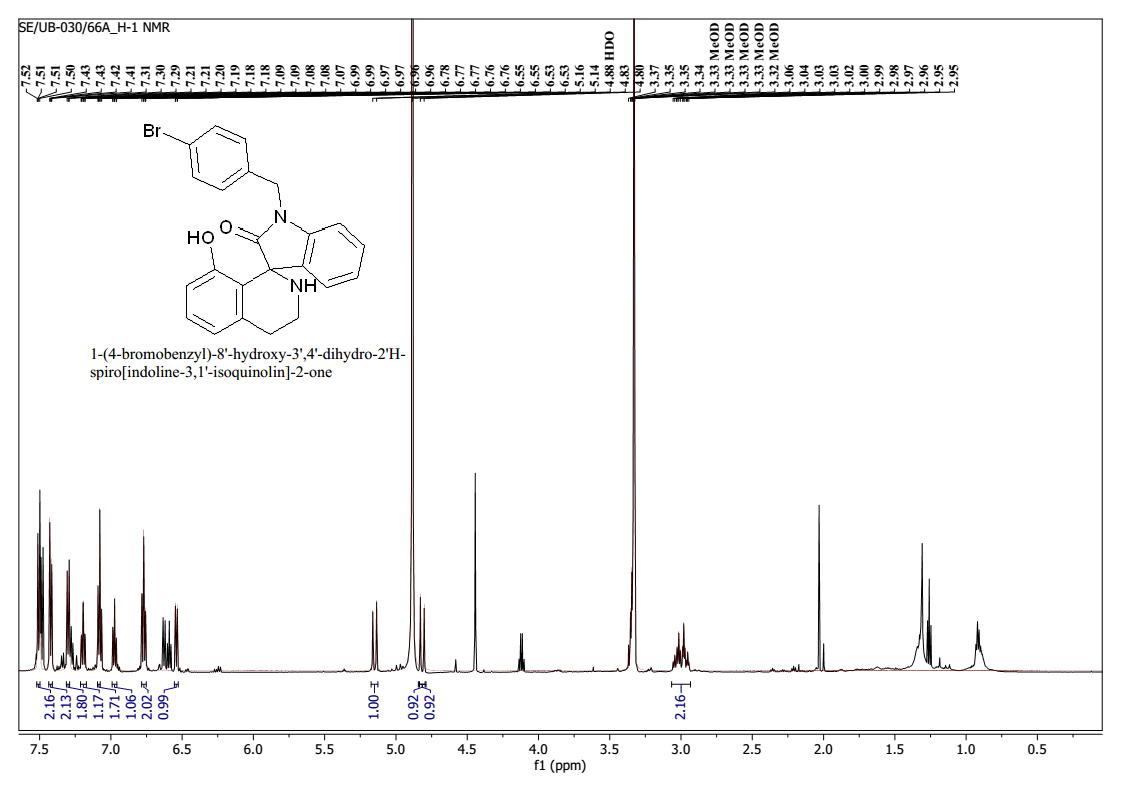
^1^H NMR** **of** **1-(4-bromobenzyl)-8'-hydroxy-3',4'-dihydro-2'H-spiro[indoline-3,1'-isoquinolin]-2-one (1f)**

**
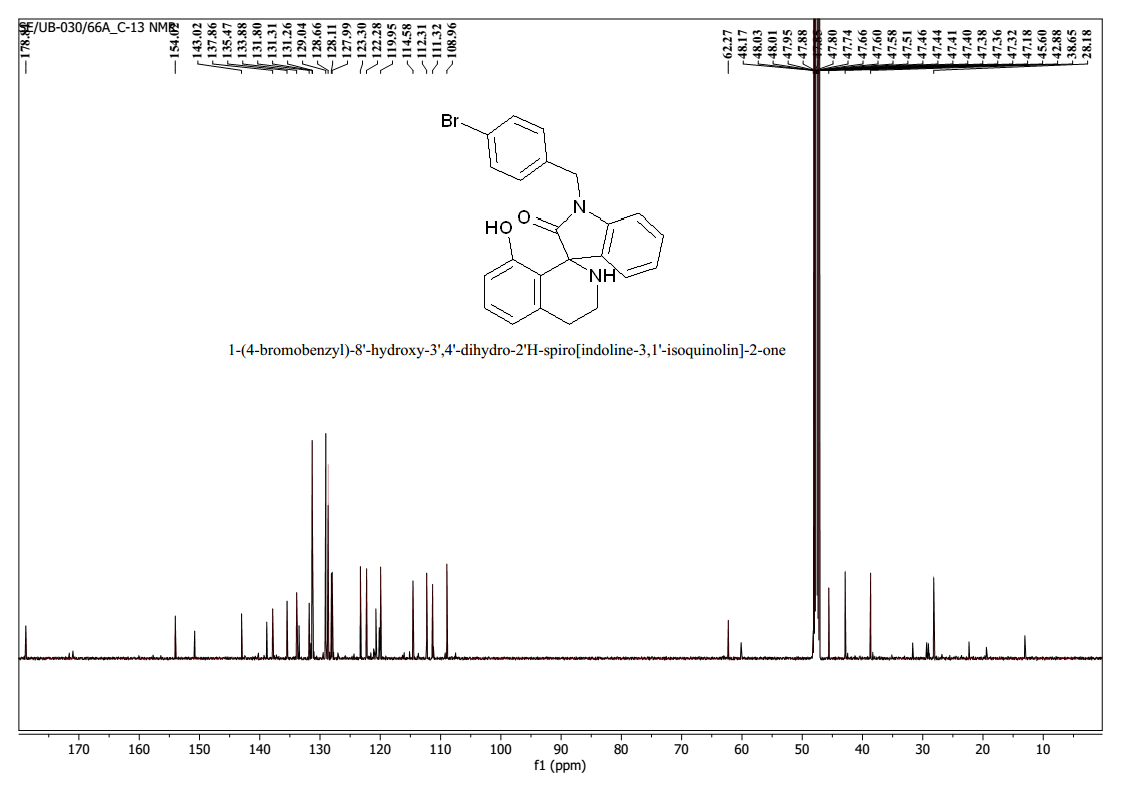
^13^C NMR of 1-(4-bromobenzyl)-8'-hydroxy-3',4'-dihydro-2'H-spiro[indoline-3,1'-isoquinolin]-2-one (1f)**

**
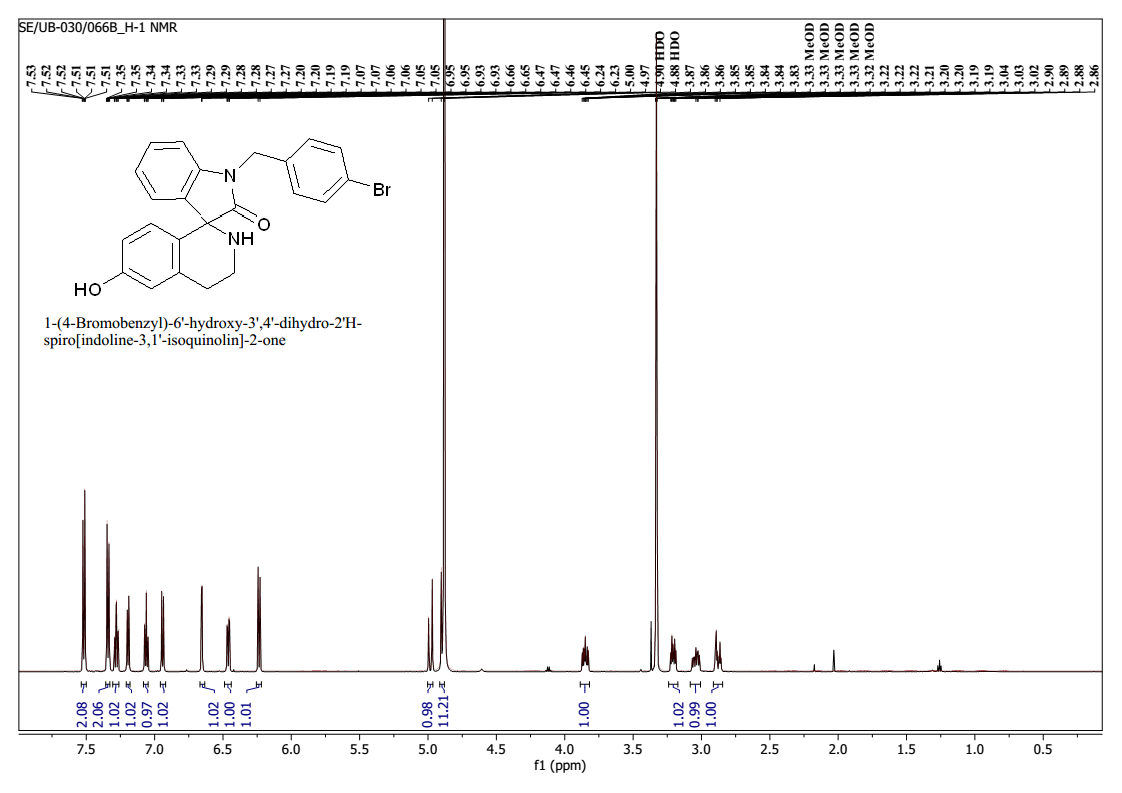
^1^H NMR** **of** **1-(4-Bromobenzyl)-6'-hydroxy-3',4'-dihydro-2'H-spiro[indoline-3,1'-isoquinolin]-2-one (2f)**

**
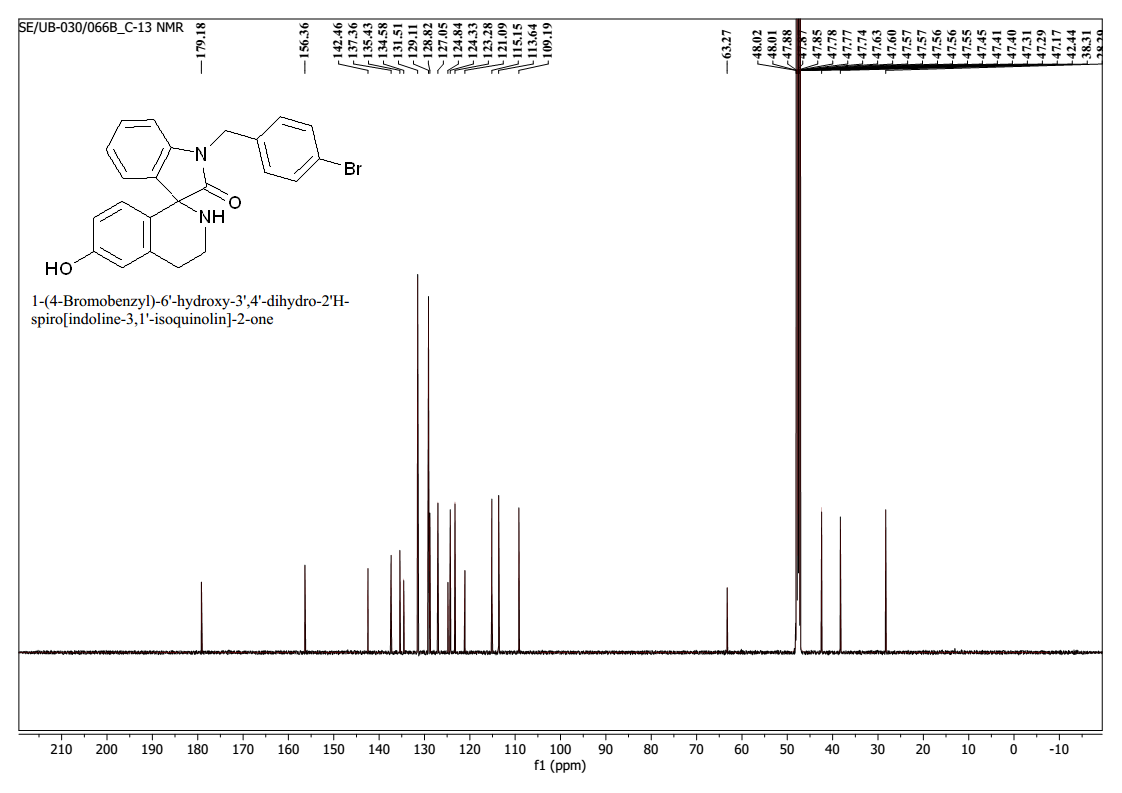
^13^C NMR of 1-(4-Bromobenzyl)-6'-hydroxy-3',4'-dihydro-2'H-spiro[indoline-3,1'-isoquinolin]-2-one (2f)**

**
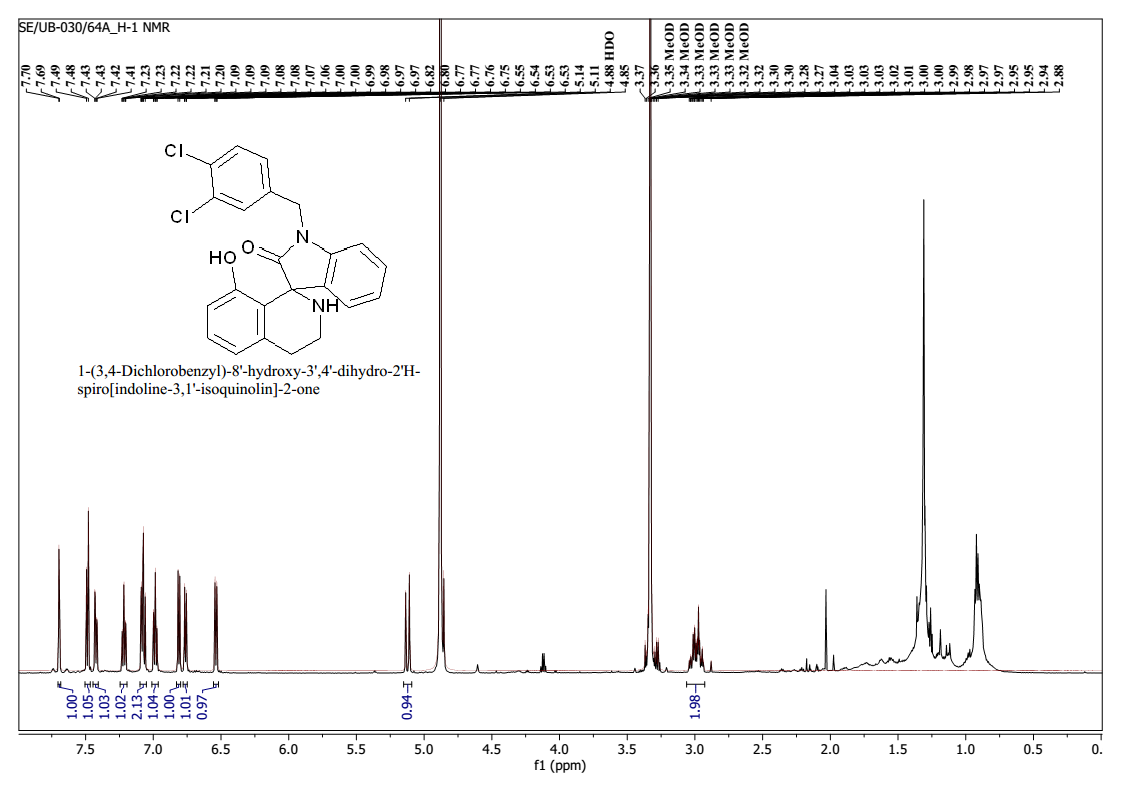
^1^H NMR** **of** **1-(3,4-Dichlorobenzyl)-8'-hydroxy-3',4'-dihydro-2'H-spiro[indoline-3,1'-isoquinolin]-2-one (1g)**

**
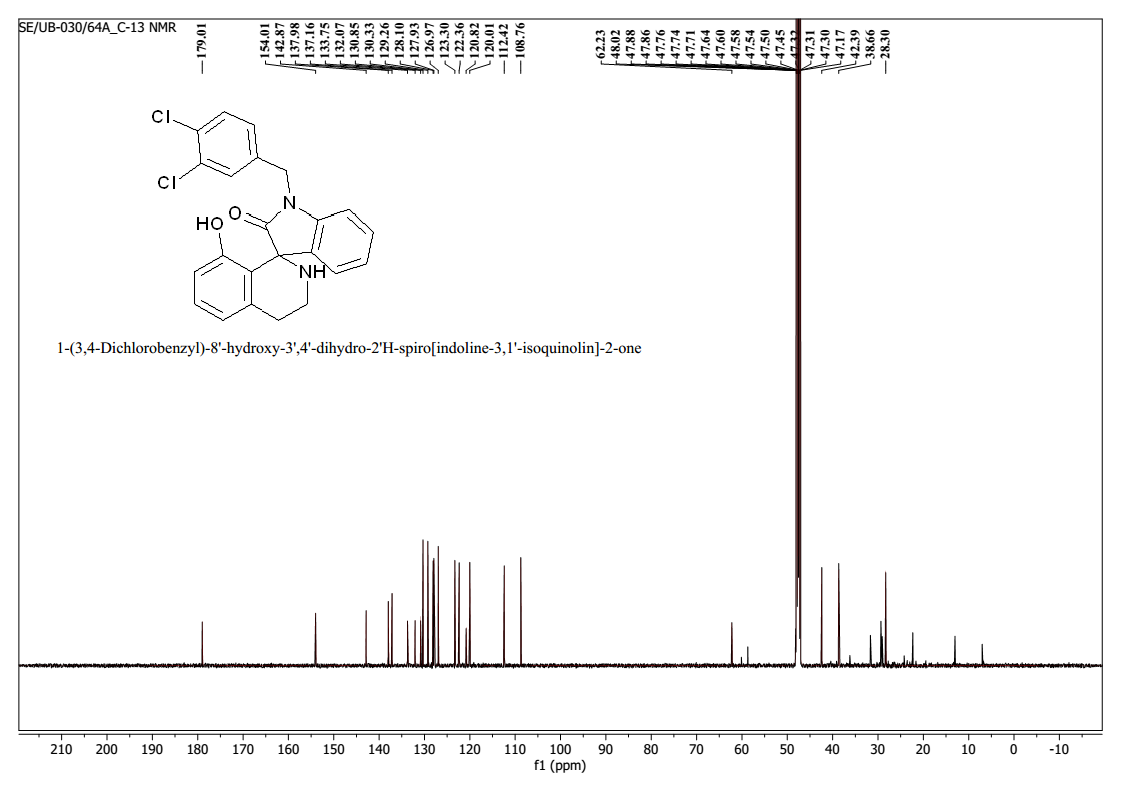
^13^C NMR of 1-(3,4-Dichlorobenzyl)-8'-hydroxy-3',4'-dihydro-2'H-spiro[indoline-3,1'-isoquinolin]-2-one (1g)**

**
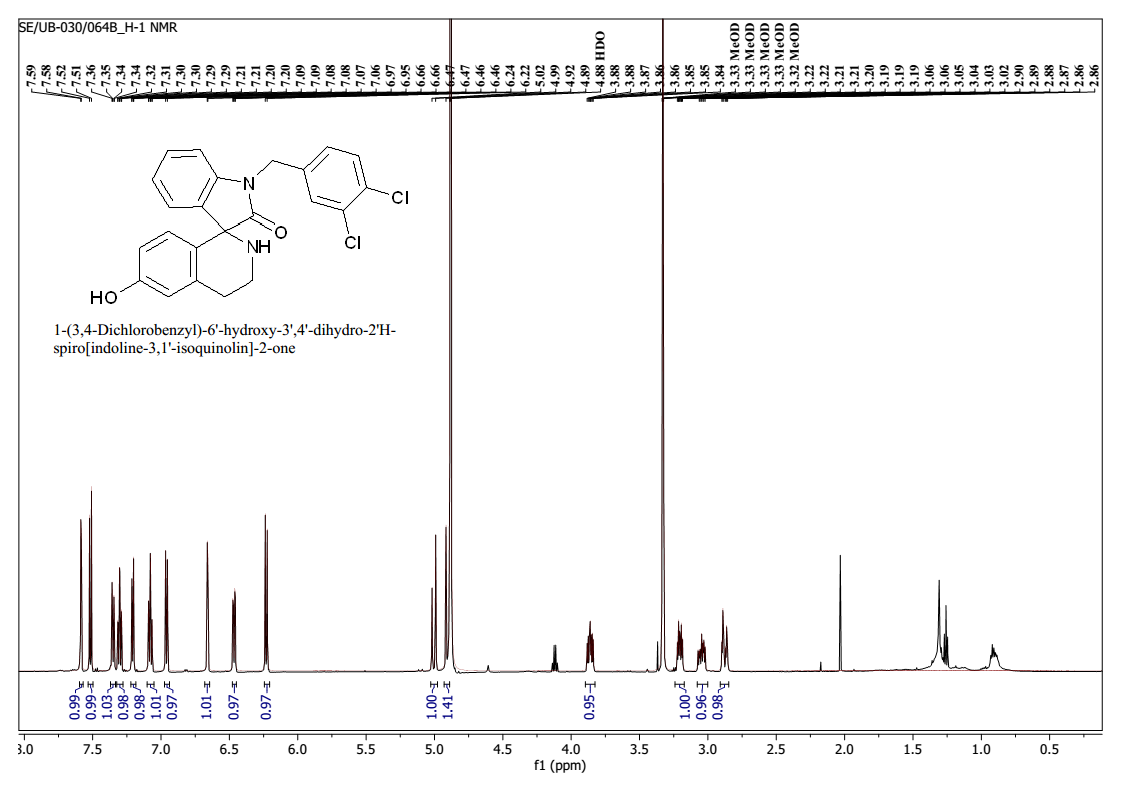
^1^H NMR** **of** **1-(3,4-Dichlorobenzyl)-6'-hydroxy-3',4'-dihydro-2'H-spiro[indoline-3,1'-isoquinolin]-2-one (2g)**

**
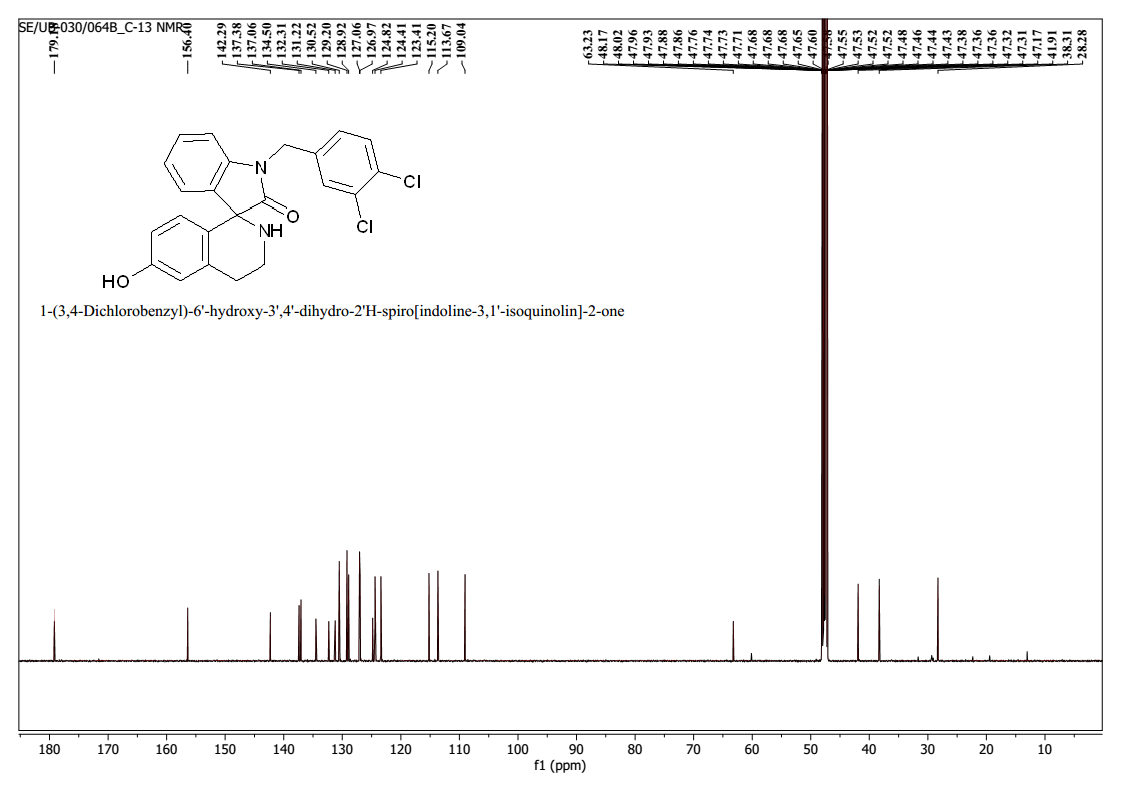
^13^C NMR of 1-(3,4-Dichlorobenzyl)-6'-hydroxy-3',4'-dihydro-2'H-spiro[indoline-3,1'-isoquinolin]-2-one (2g)**

**
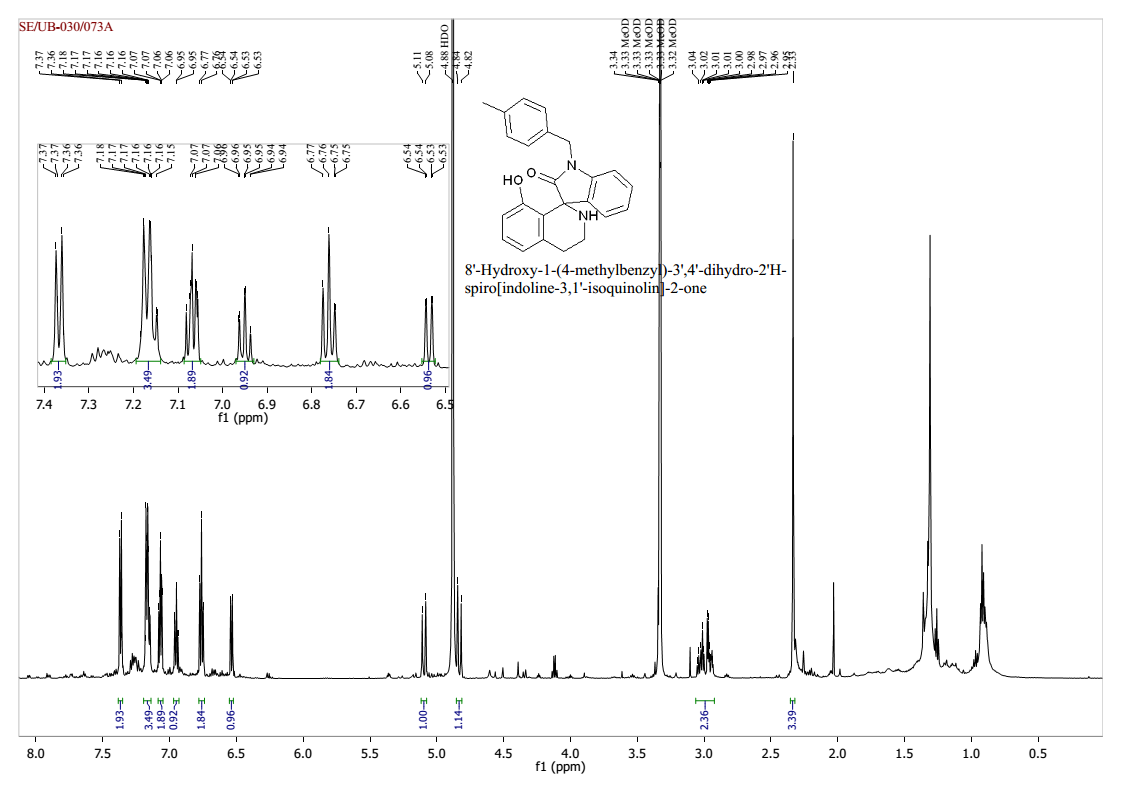
^1^H NMR** **of** **8'-Hydroxy-1-(4-methylbenzyl)-3',4'-dihydro-2'H-spiro[indoline-3,1'-isoquinolin]-2-one (1h)**

**
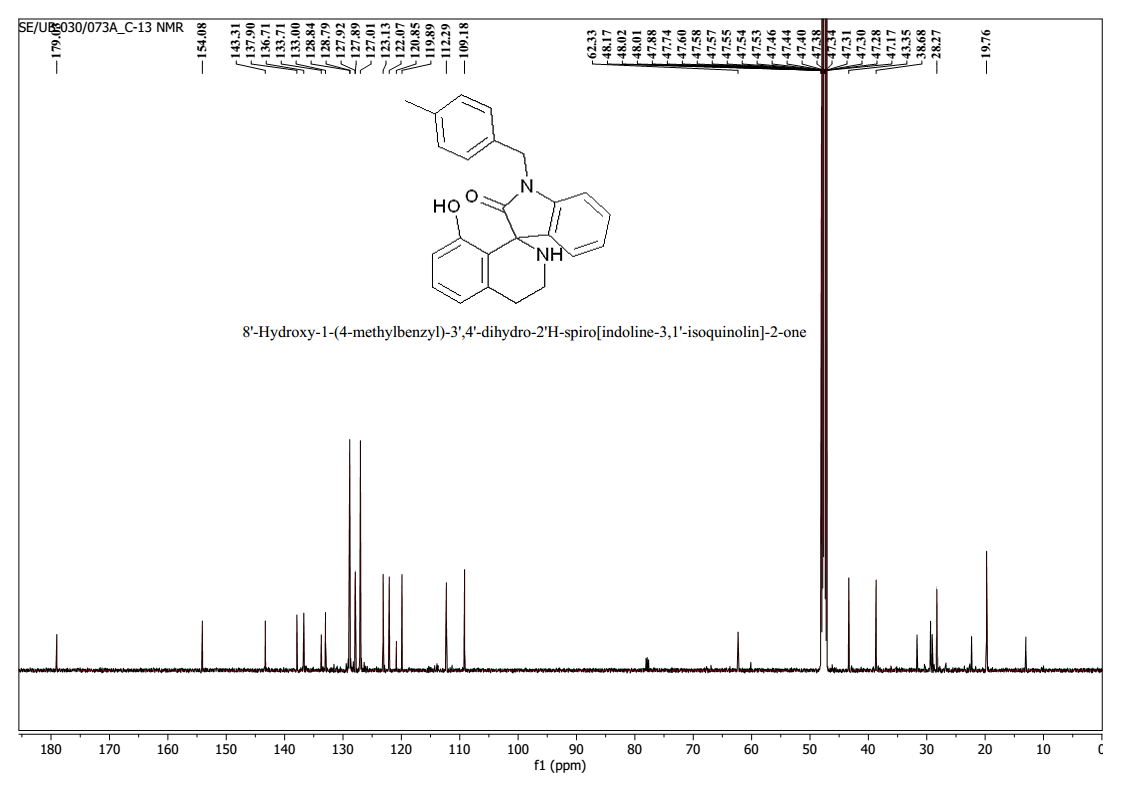
^13^C NMR of 8'-Hydroxy-1-(4-methylbenzyl)-3',4'-dihydro-2'H-spiro[indoline-3,1'-isoquinolin]-2-one (1h)**

**
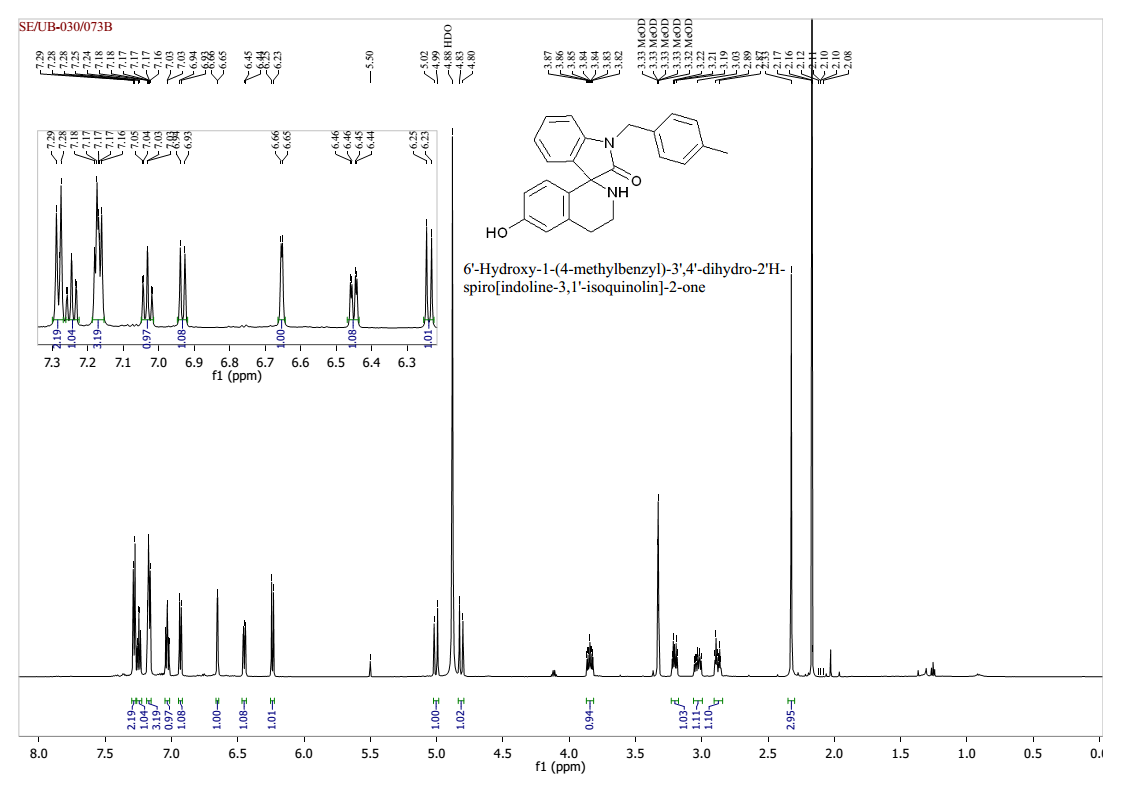
^1^H NMR** **of** **6'-Hydroxy-1-(4-methylbenzyl)-3',4'-dihydro-2'H-spiro[indoline-3,1'-isoquinolin]-2-one (2h)**

**
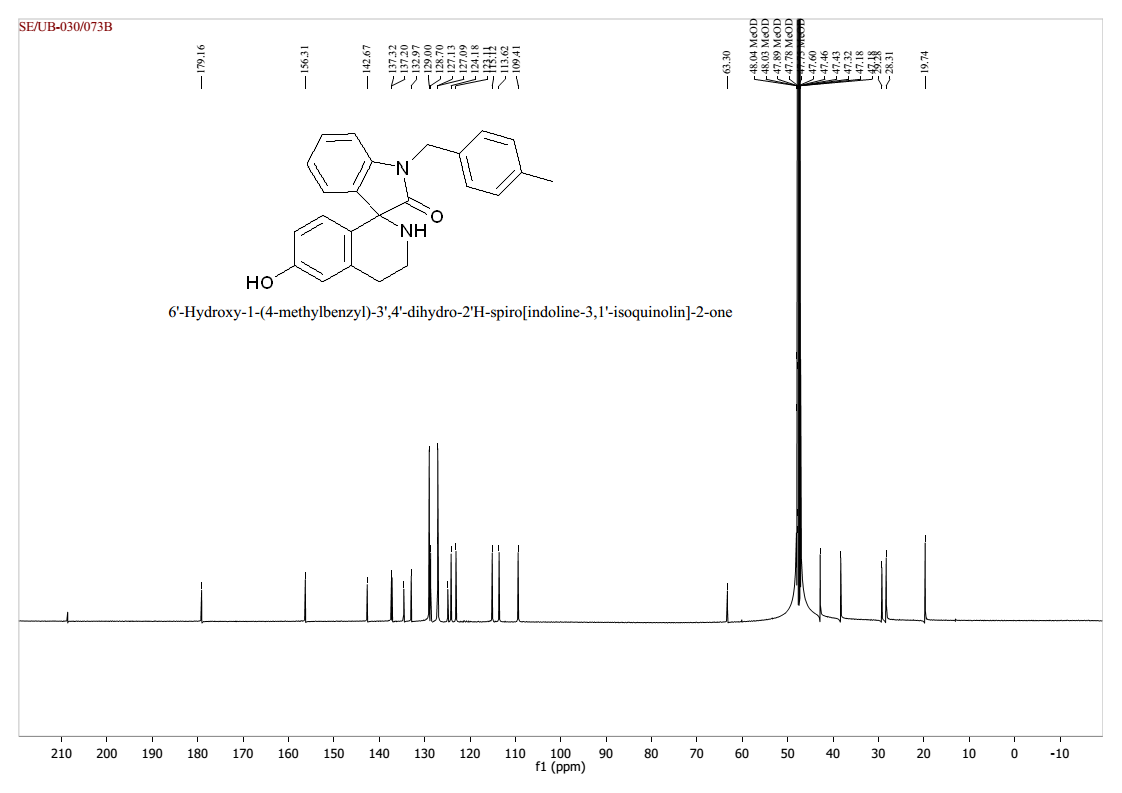
^13^C NMR of 6'-Hydroxy-1-(4-methylbenzyl)-3',4'-dihydro-2'H-spiro[indoline-3,1'-isoquinolin]-2-one (2h)**

**
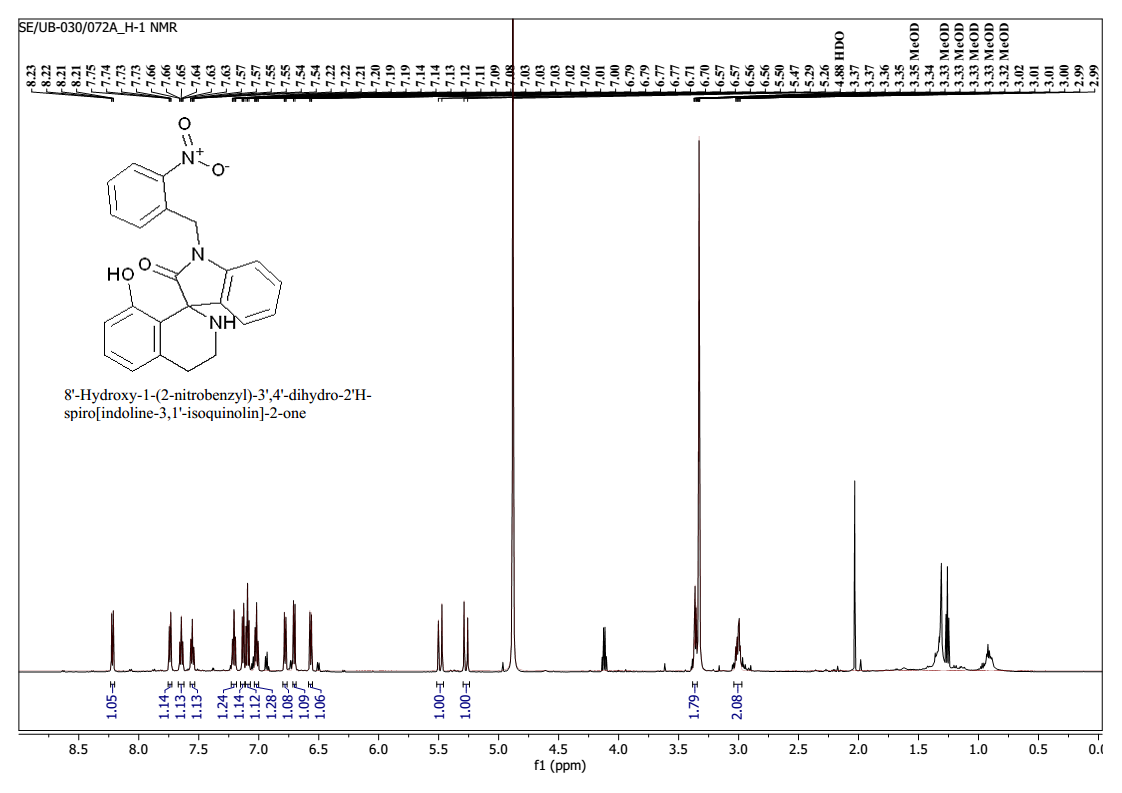
^1^H NMR** **of** **8'-Hydroxy-1-(2-nitrobenzyl)-3',4'-dihydro-2'H-spiro[indoline-3,1'-isoquinolin]-2-one (1i)**

**
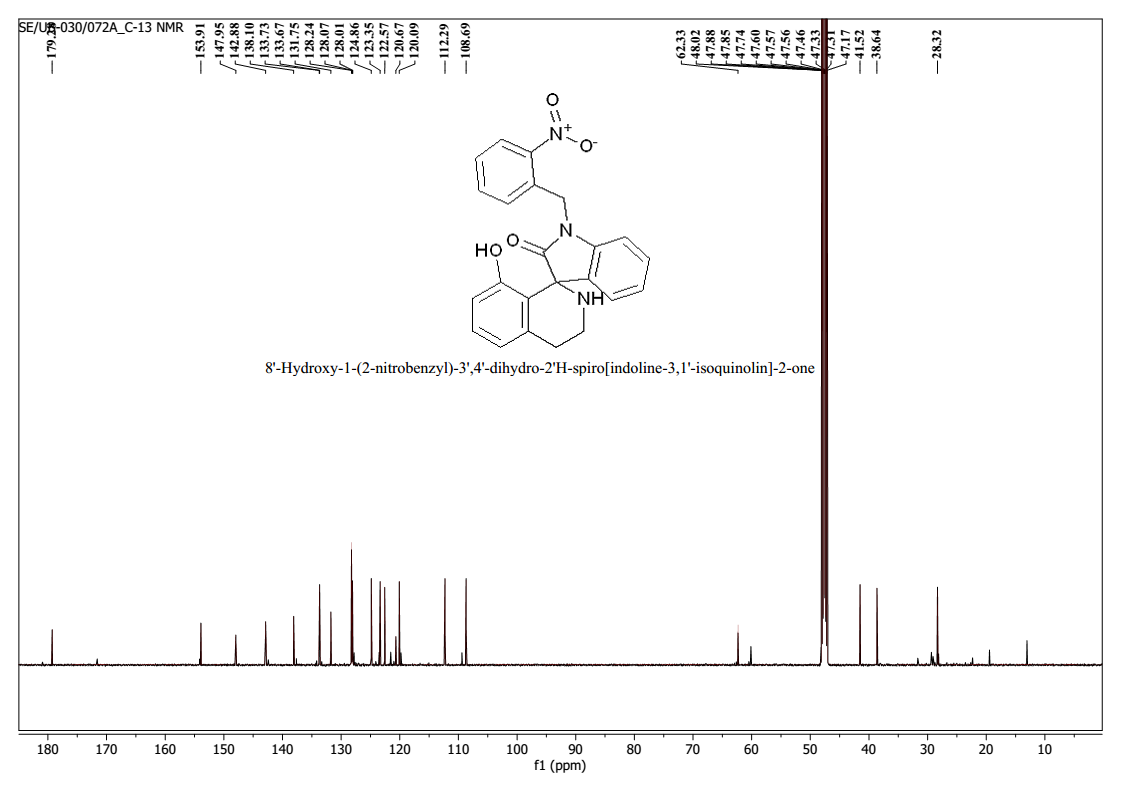
^13^C NMR of 8'-Hydroxy-1-(2-nitrobenzyl)-3',4'-dihydro-2'H-spiro[indoline-3,1'-isoquinolin]-2-one (1i)**

**
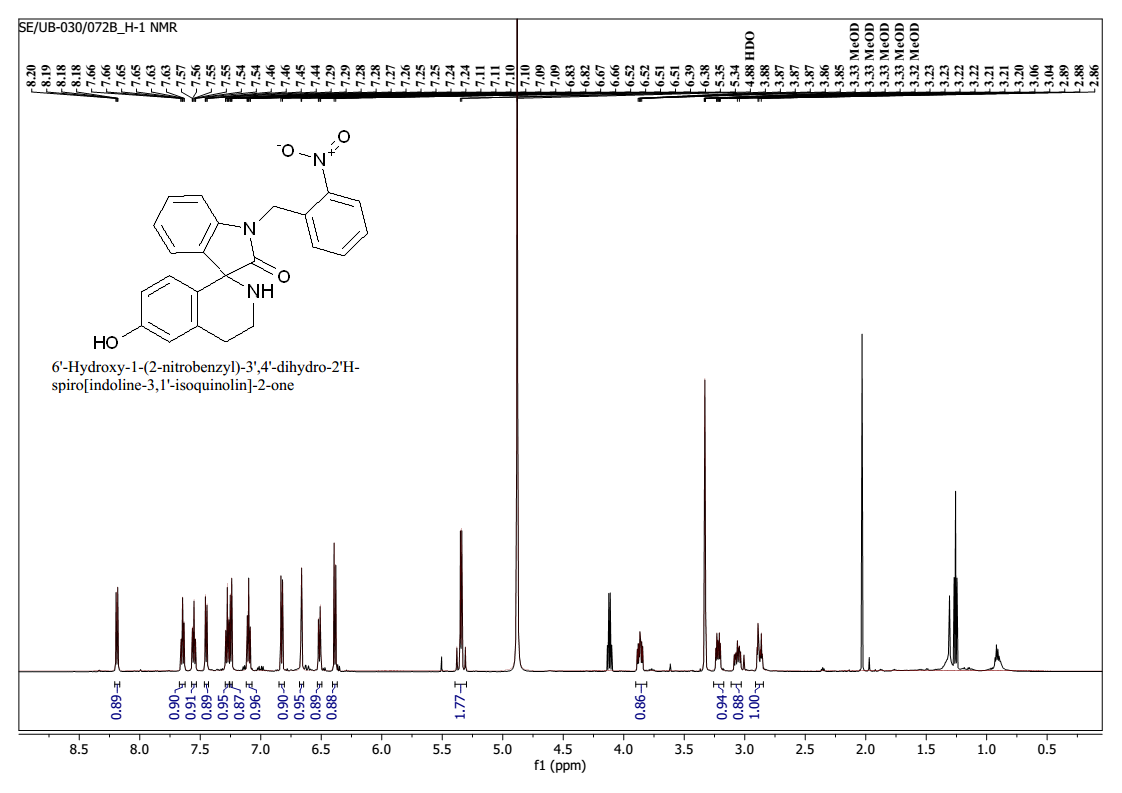
^1^H NMR** **of** **6'-Hydroxy-1-(2-nitrobenzyl)-3',4'-dihydro-2'H-spiro[indoline-3,1'-isoquinolin]-2-one (2i)**

**
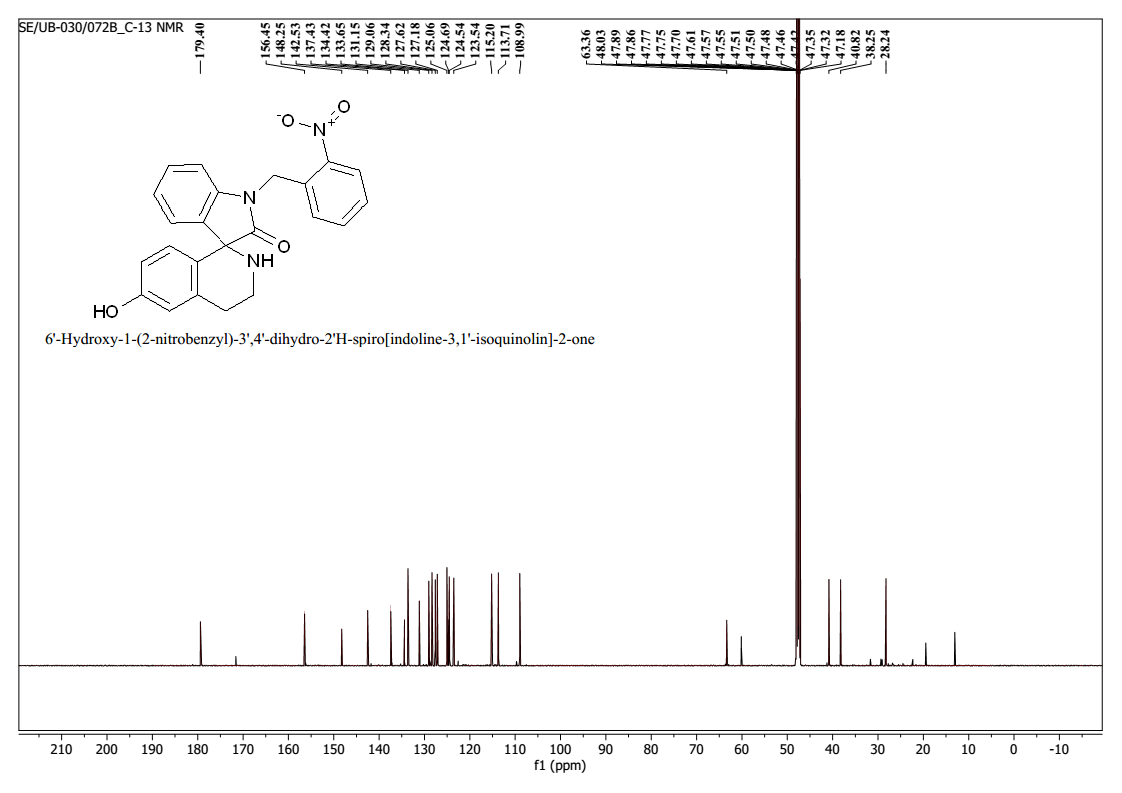
^13^C NMR of 6'-Hydroxy-1-(2-nitrobenzyl)-3',4'-dihydro-2'H-spiro[indoline-3,1'-isoquinolin]-2-one (2i)**

**
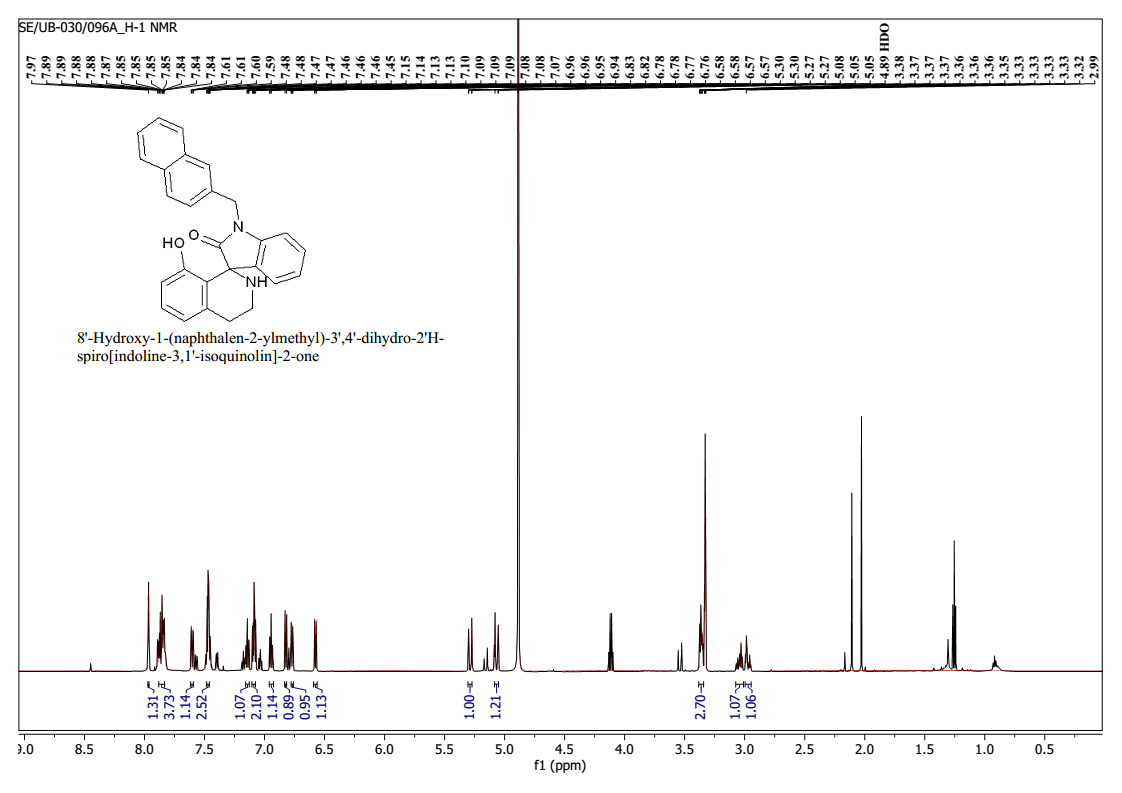
^1^H NMR** **of** **8’-Hydroxy-1-(naphthalen-2-ylmethyl)-3',4'-dihydro-2'H-spiro[indoline-3,1'-isoquinolin]-2-one (1j)**

**
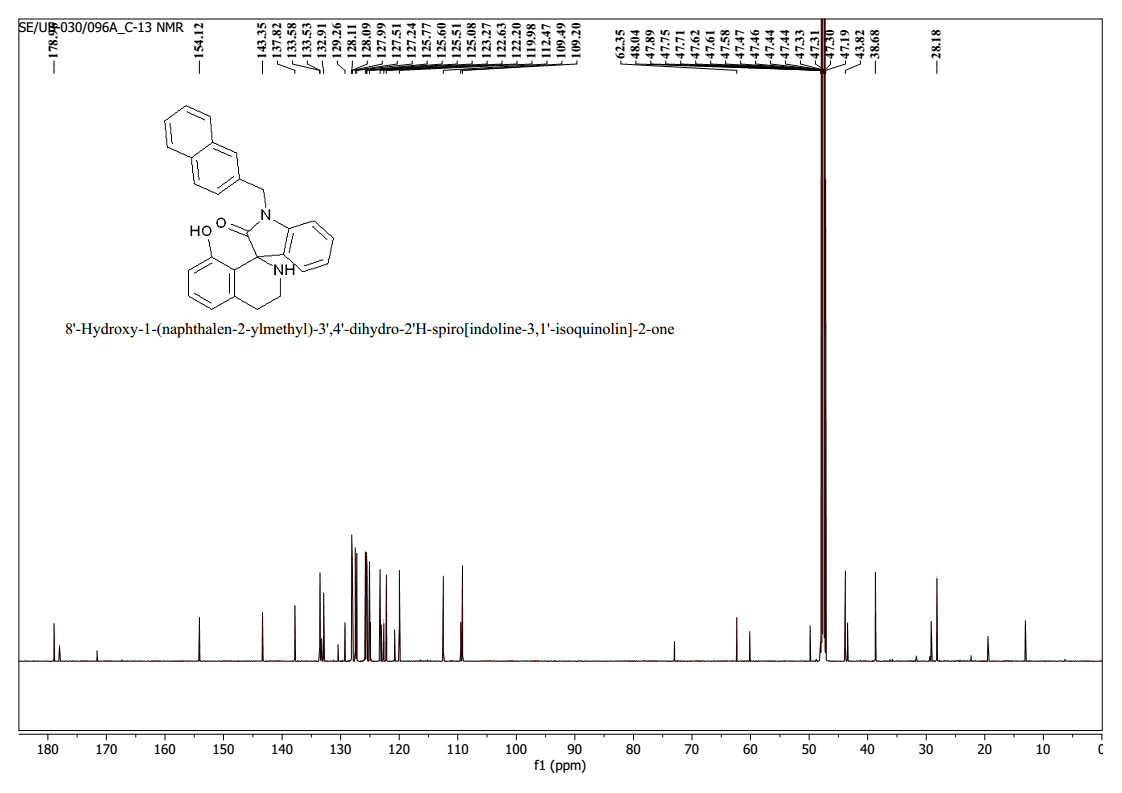
^13^C NMR of 8’-Hydroxy-1-(naphthalen-2-ylmethyl)-3',4'-dihydro-2'H-spiro[indoline-3,1'-isoquinolin]-2-one (1j)**

**
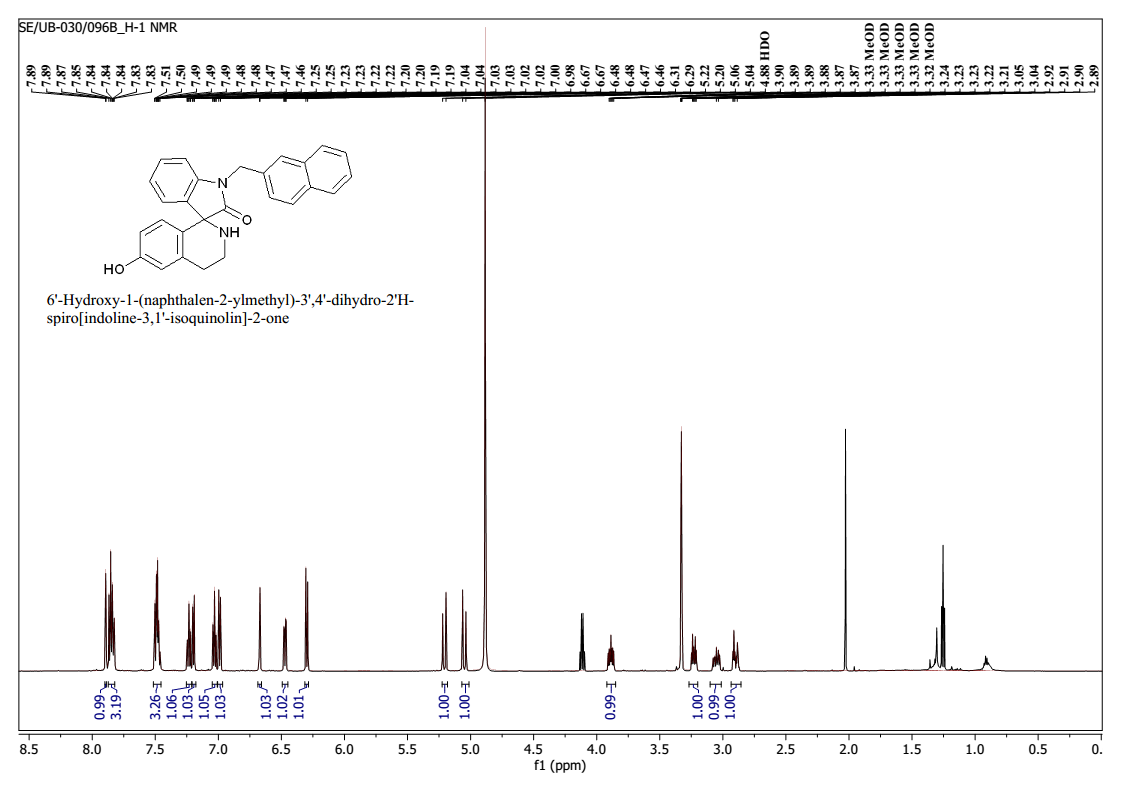
^1^H NMR** **of** **6’-Hydroxy-1-(naphthalen-2-ylmethyl)-3',4'-dihydro-2'H-spiro[indoline-3,1'-isoquinolin]-2-one (2j)**

**
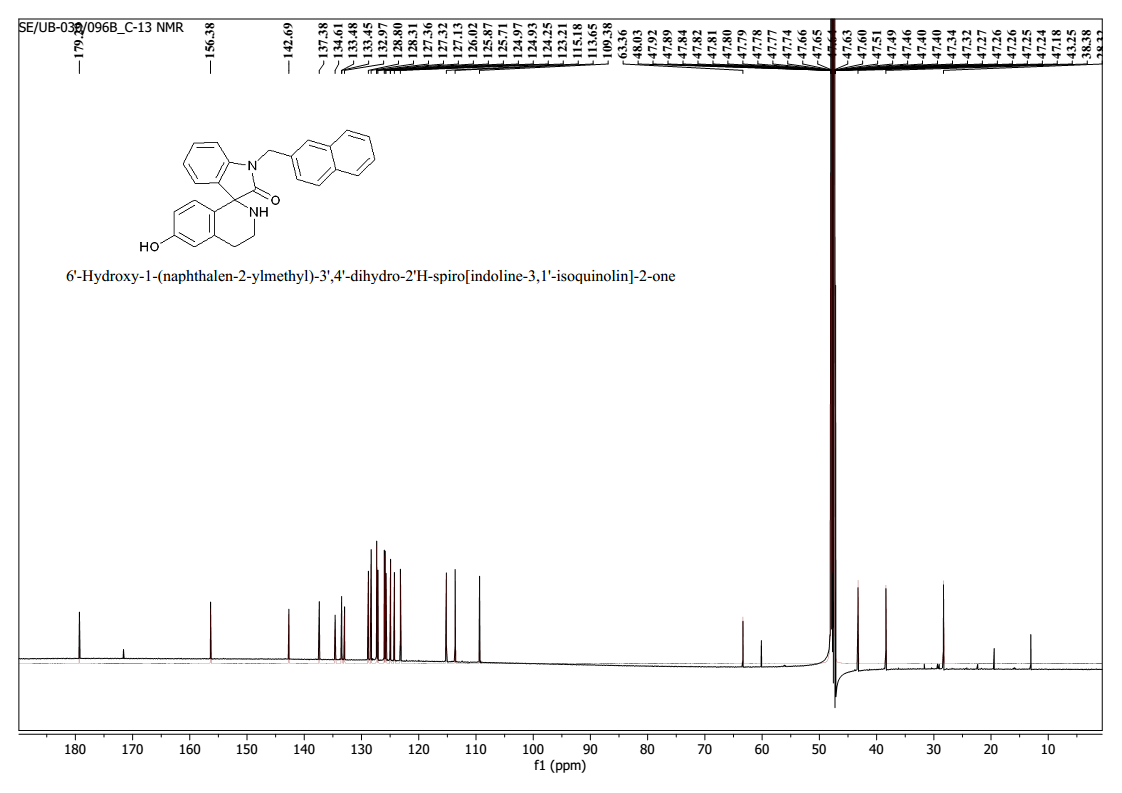
^13^C NMR of 6’-Hydroxy-1-(naphthalen-2-ylmethyl)-3',4'-dihydro-2'H-spiro[indoline-3,1'-isoquinolin]-2-one (2j)**

**
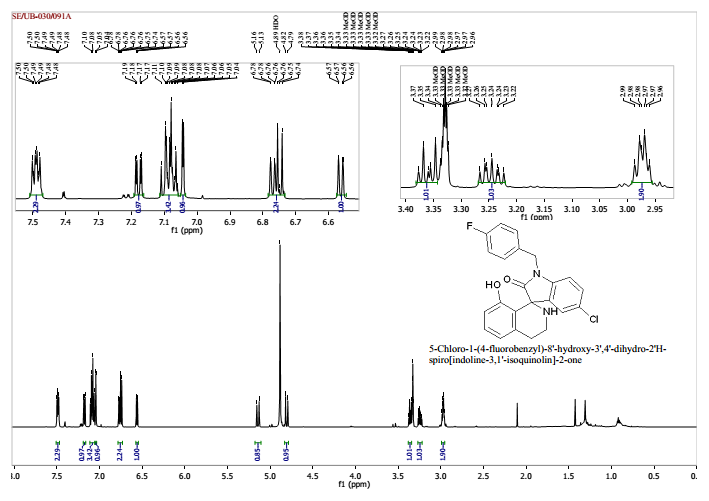
^1^H NMR** **of** **5-Chloro-1-(4-fluorobenzyl)-8'-hydroxy-3',4'-dihydro-2'H-spiro[indoline-3,1'-isoquinolin]-2-one (1k)**

**
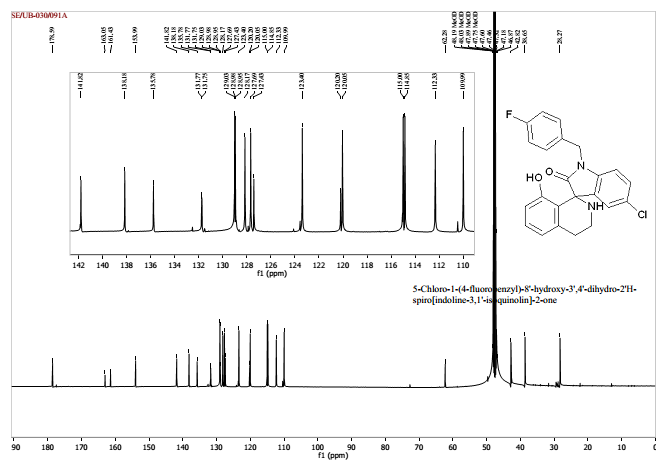
^13^C NMR of 5-Chloro-1-(4-fluorobenzyl)-8'-hydroxy-3',4'-dihydro-2'H-spiro[indoline-3,1'-isoquinolin]-2-one (1k)**

**
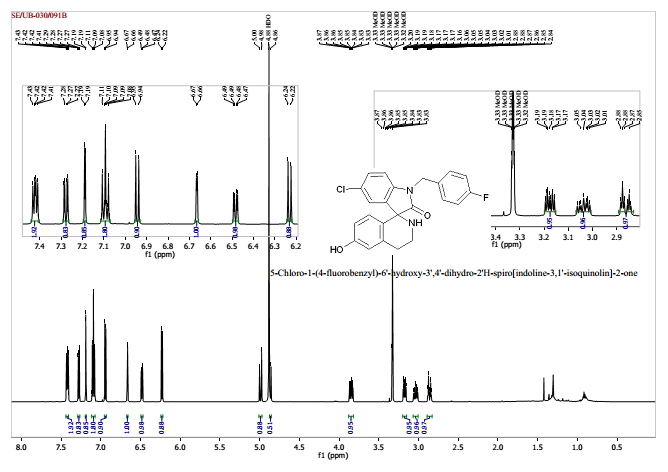
^1^H NMR** **of** **5-Chloro-1-(4-fluorobenzyl)-6'-hydroxy-3',4'-dihydro-2'H-spiro[indoline-3,1'-isoquinolin]-2-one (2k)**

**
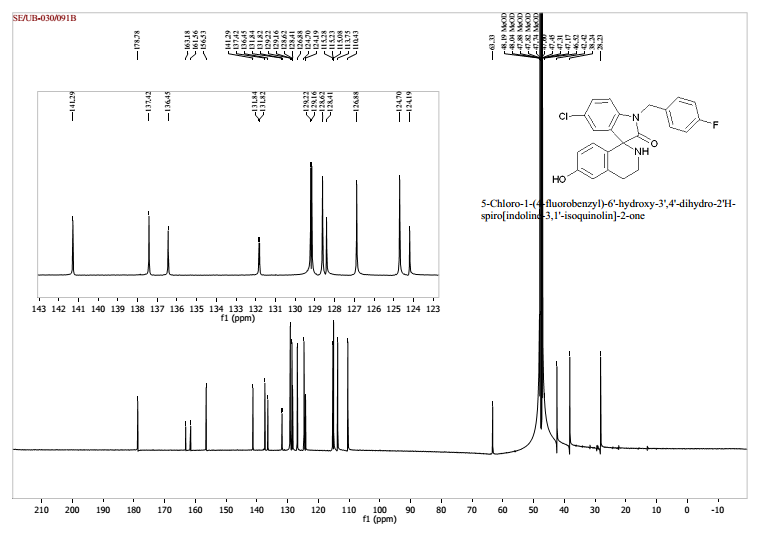
^13^C NMR of 5-Chloro-1-(4-fluorobenzyl)-6'-hydroxy-3',4'-dihydro-2'H-spiro[indoline-3,1'-isoquinolin]-2-one (2k)**

**
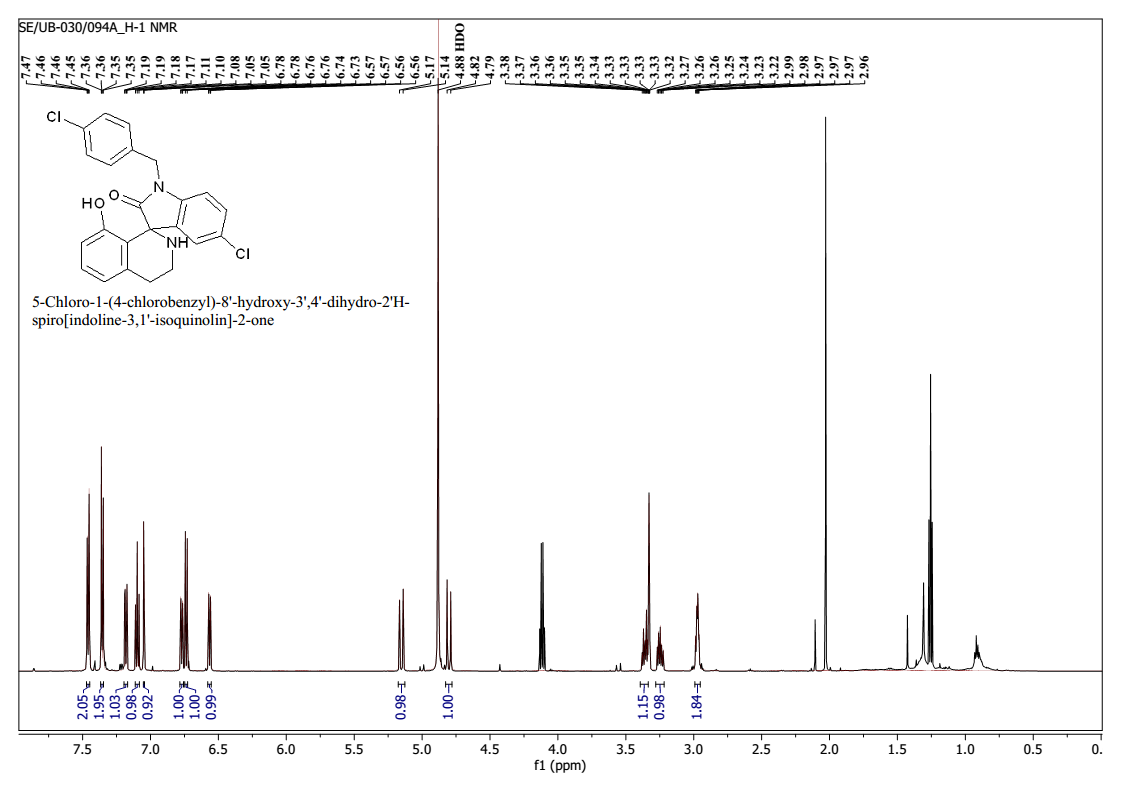
^1^H NMR** **of** **5-Chloro-1-(4-chlorobenzyl)-8'-hydroxy-3',4'-dihydro-2'H-spiro[indoline-3,1'-isoquinolin]-2-one (1l)**

**
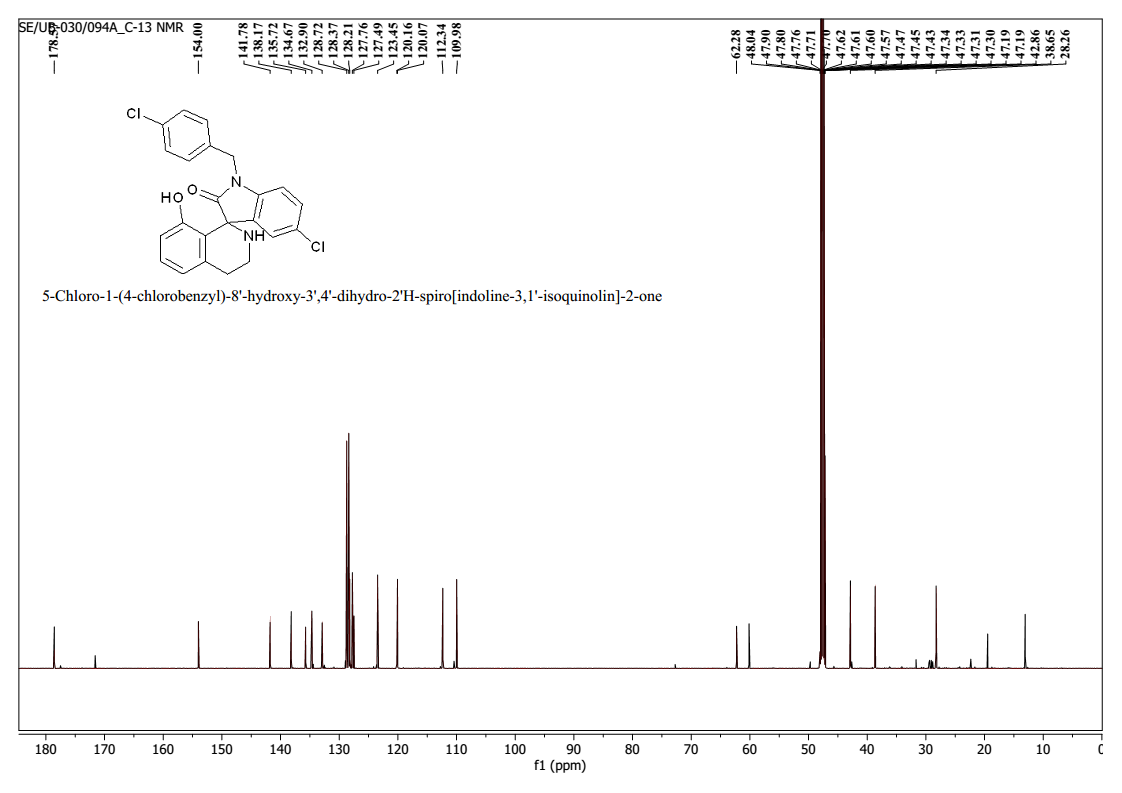
^13^C NMR of 5-Chloro-1-(4-chlorobenzyl)-8'-hydroxy-3',4'-dihydro-2'H-spiro[indoline-3,1'-isoquinolin]-2-one (1l)**

**
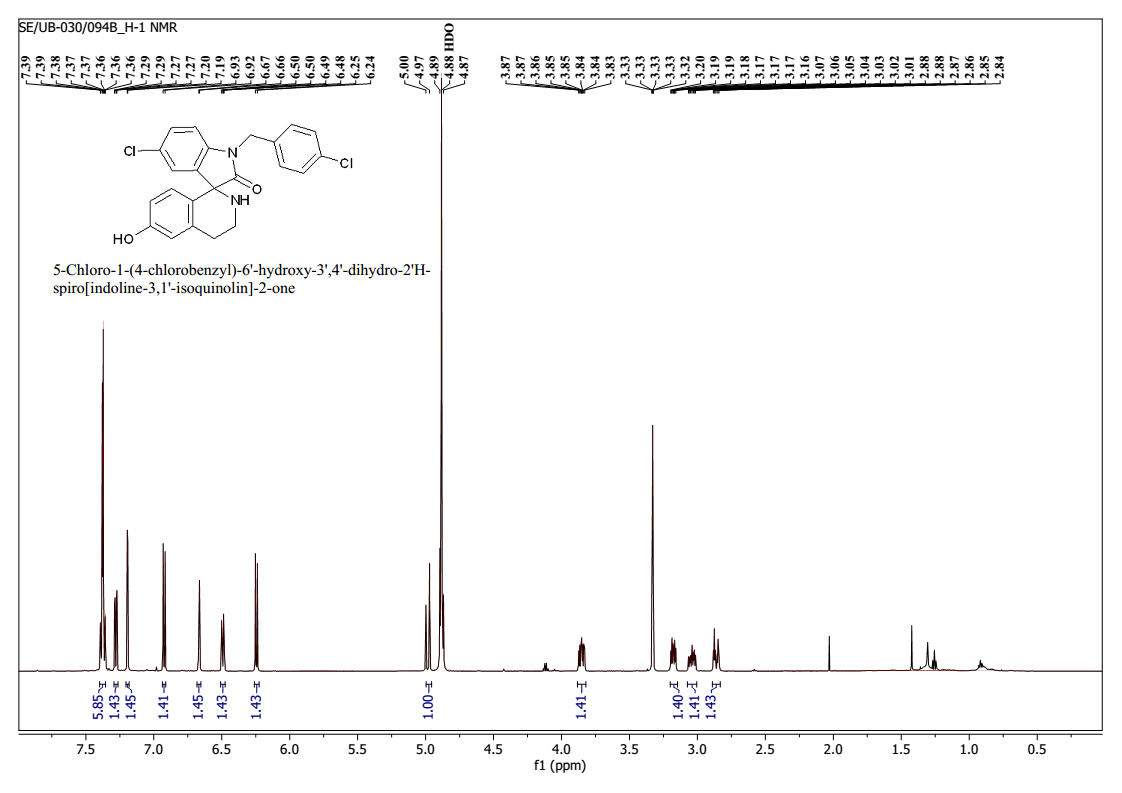
^1^H NMR** **of** **5-Chloro-1-(4-chlorobenzyl)-6'-hydroxy-3',4'-dihydro-2'H-spiro[indoline-3,1'-isoquinolin]-2-one (2l)**

**
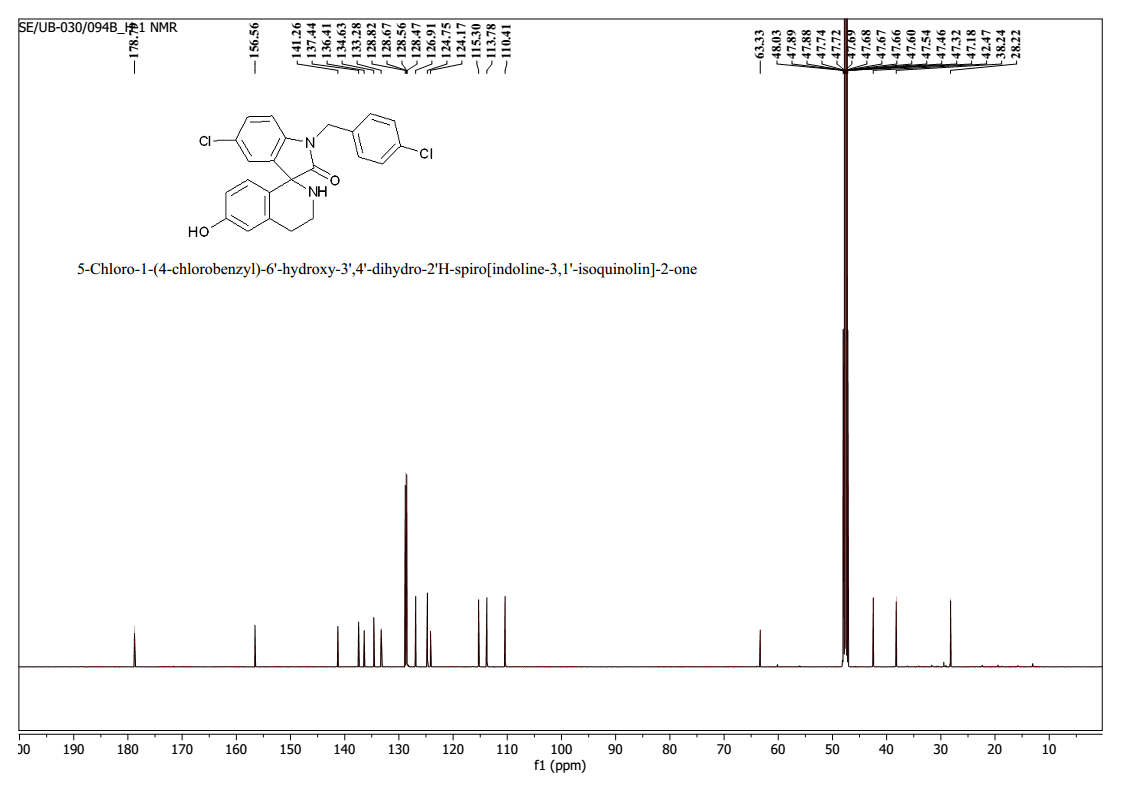
^13^C NMR of 5-Chloro-1-(4-chlorobenzyl)-6'-hydroxy-3',4'-dihydro-2'H-spiro[indoline-3,1'-isoquinolin]-2-one (2l)**

**
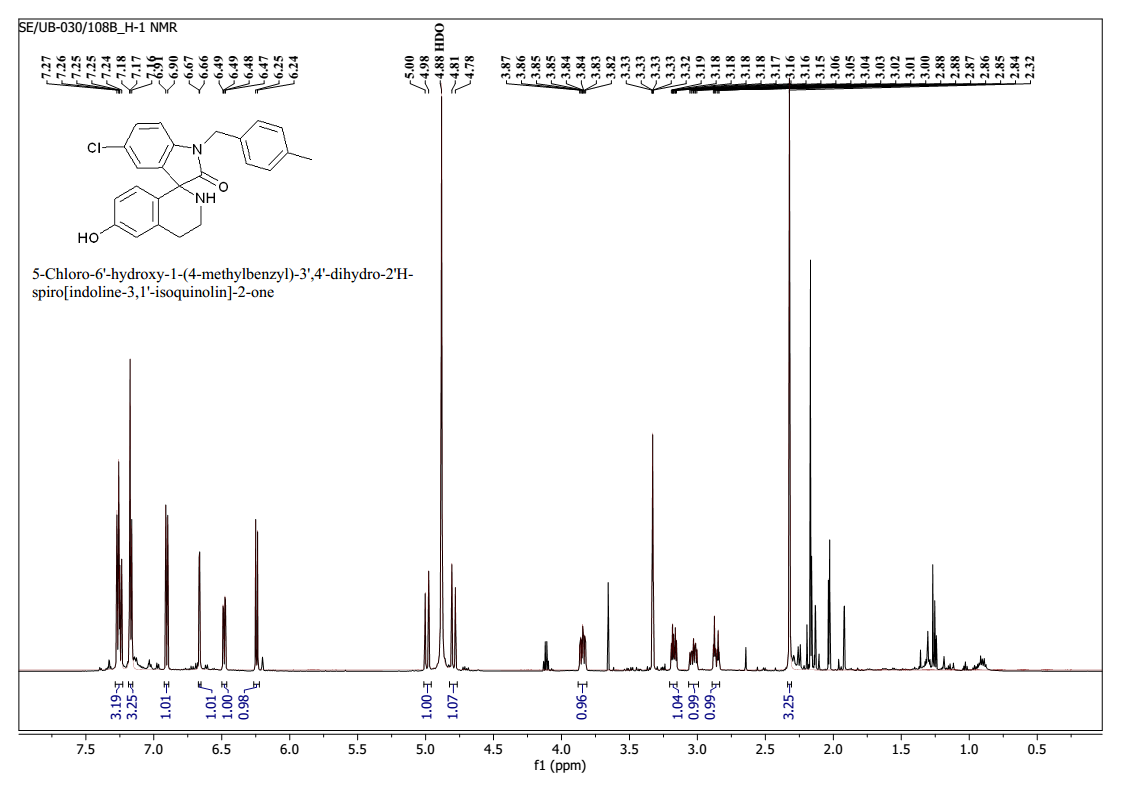
^1^H NMR** **of** **5-Chloro-6'-hydroxy-1-(4-methylbenzyl)-3',4'-dihydro-2'H-spiro[indoline-3,1'-isoquinolin]-2-one (2o)**

**
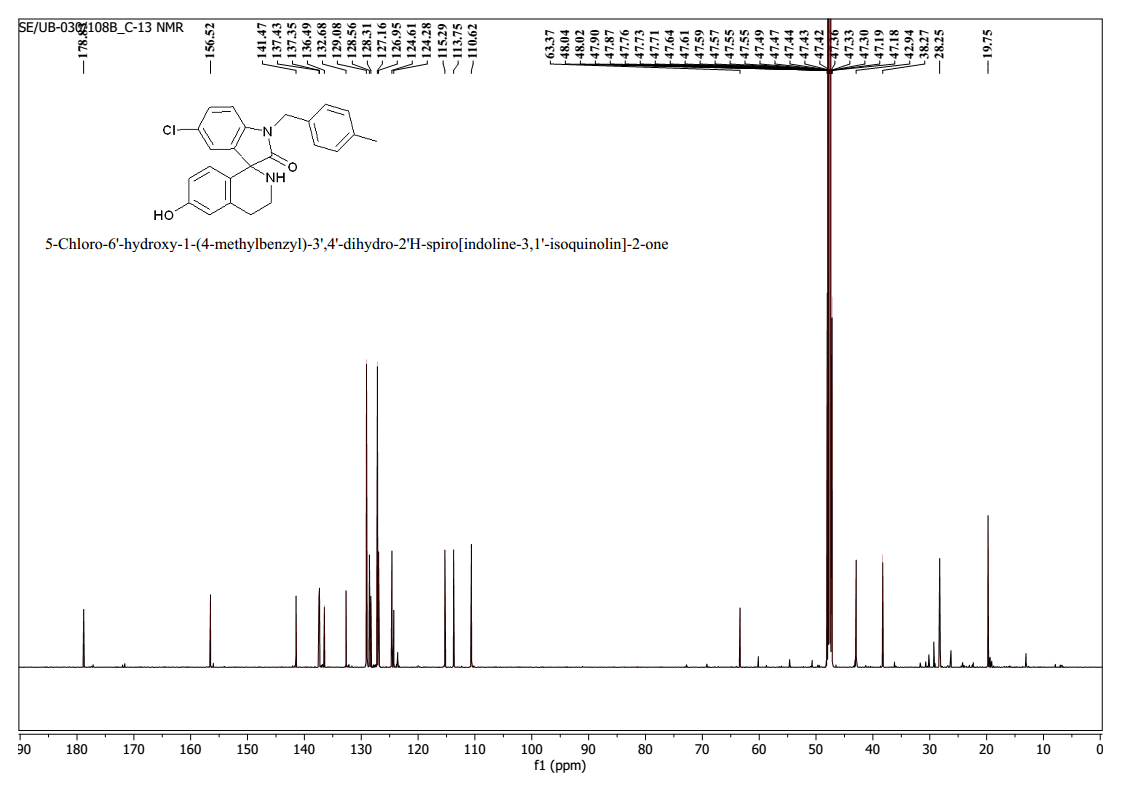
^13^C NMR of 5-Chloro-6'-hydroxy-1-(4-methylbenzyl)-3',4'-dihydro-2'H-spiro[indoline-3,1'-isoquinolin]-2-one (2o)**

**
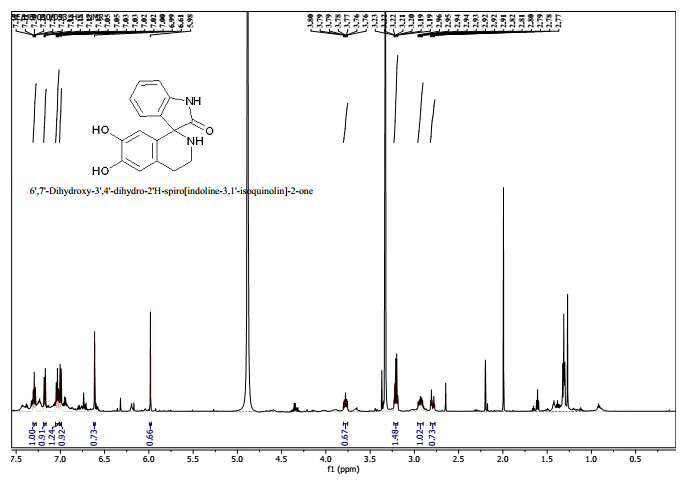
^1^H NMR** **of** **6',7'-Dihydroxy-3',4'-dihydro-2'H-spiro[indoline-3,1'-isoquinolin]-2-one (3a)**

**
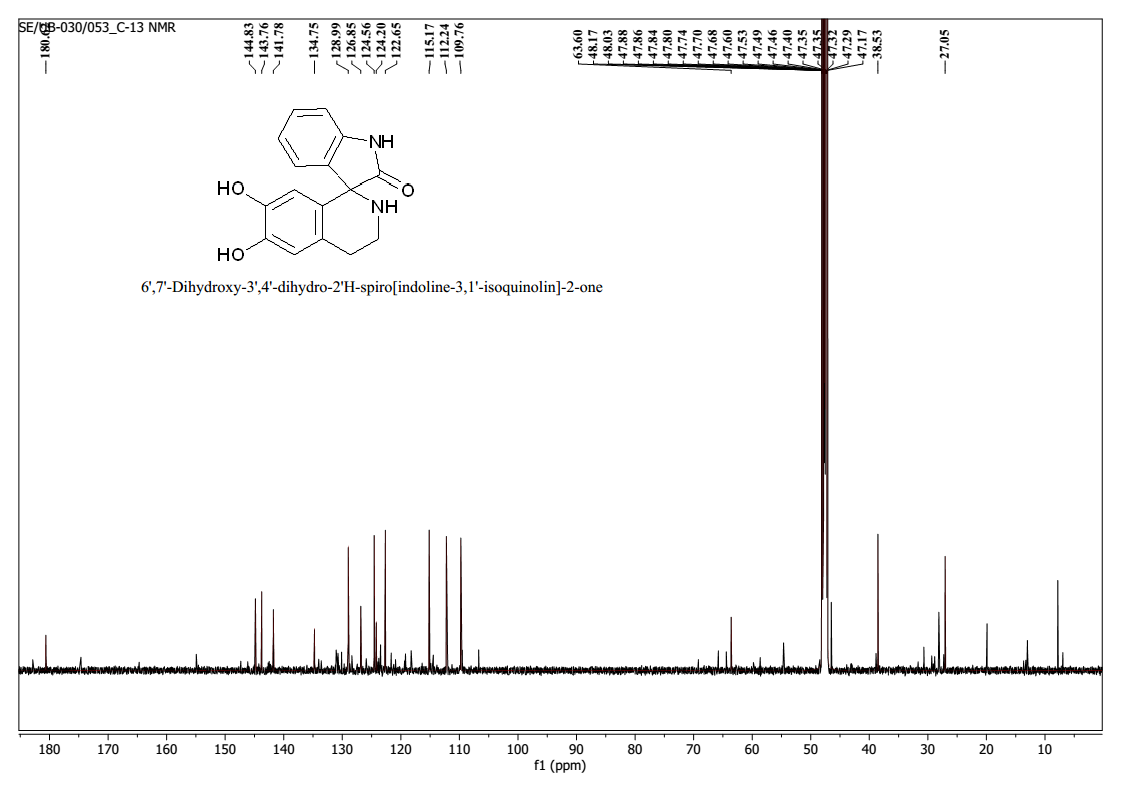
^13^C NMR of 6',7'-Dihydroxy-3',4'-dihydro-2'H-spiro[indoline-3,1'-isoquinolin]-2-one (3a)**

**
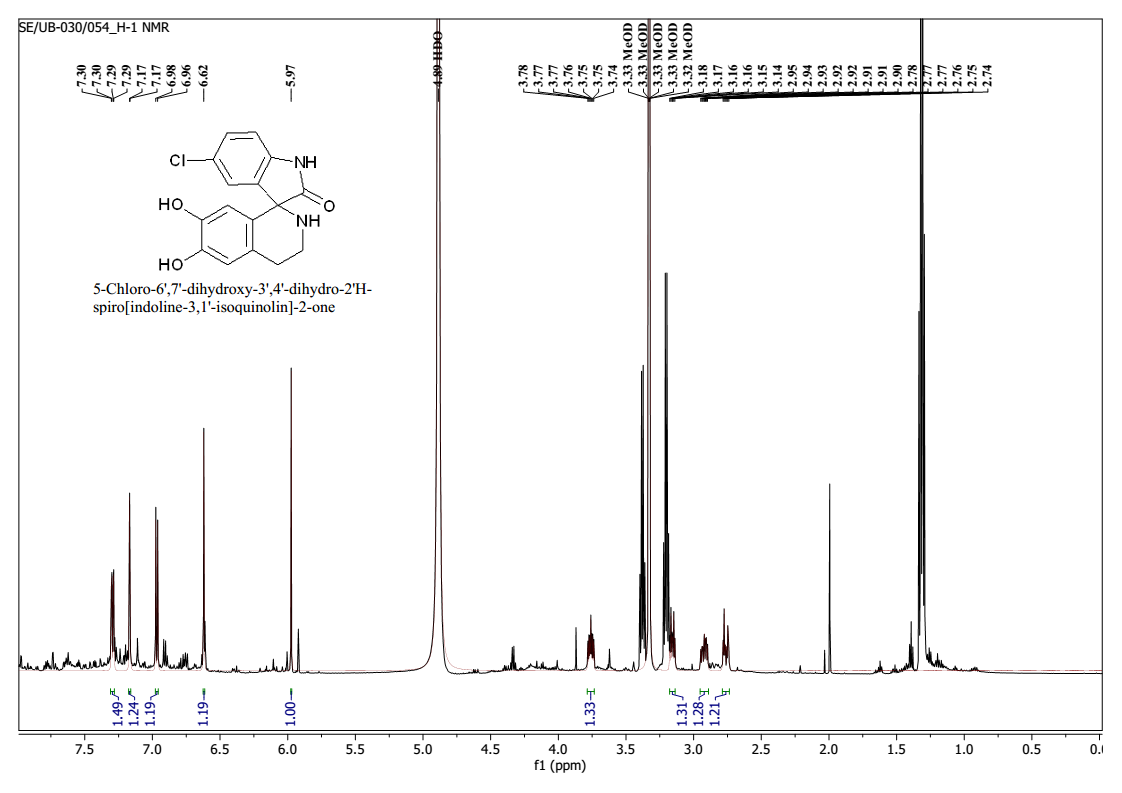
^1^H NMR** **of** **5-Chloro-6',7'-dihydroxy-3',4'-dihydro-2'H-spiro[indoline-3,1'-isoquinolin]-2-one (3b)**

**
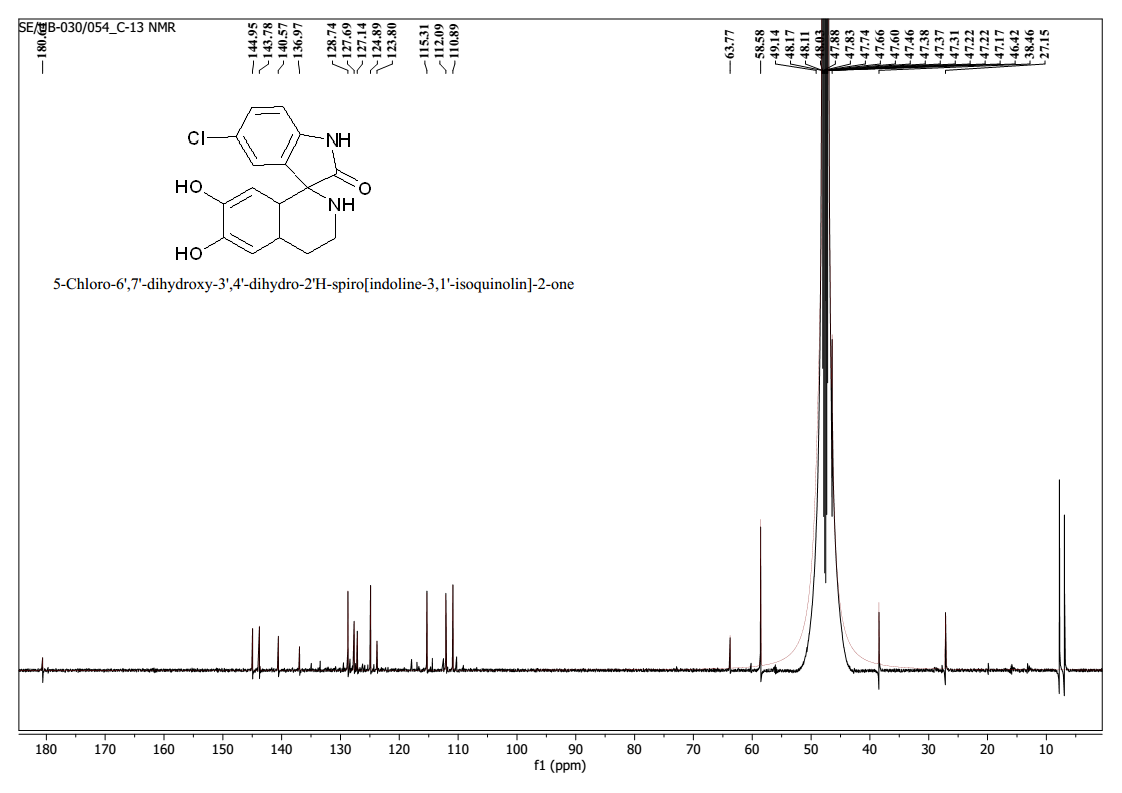
^13^C NMR of 5-Chloro-6',7'-dihydroxy-3',4'-dihydro-2'H-spiro[indoline-3,1'-isoquinolin]-2-one (3b)**

**
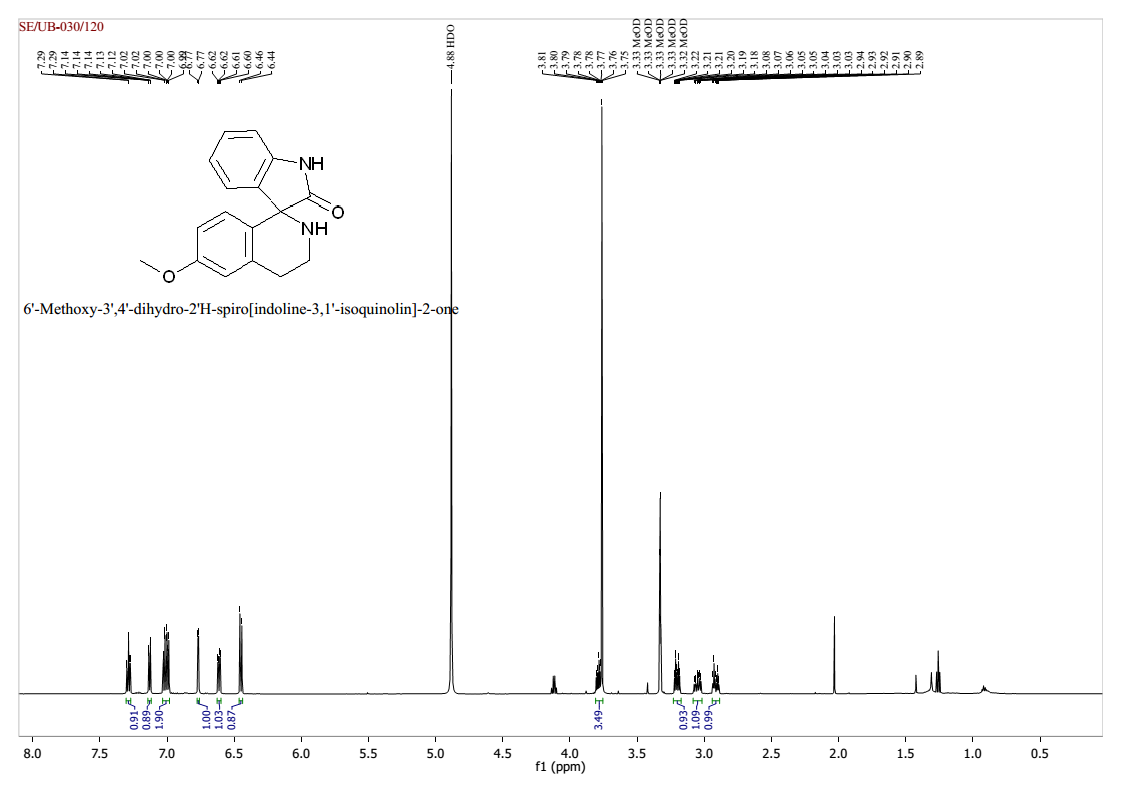
^1^H NMR** **of** **6'-Methoxy-3',4'-dihydro-2'H-spiro[indoline-3,1'-isoquinolin]-2-one (4a)**

**
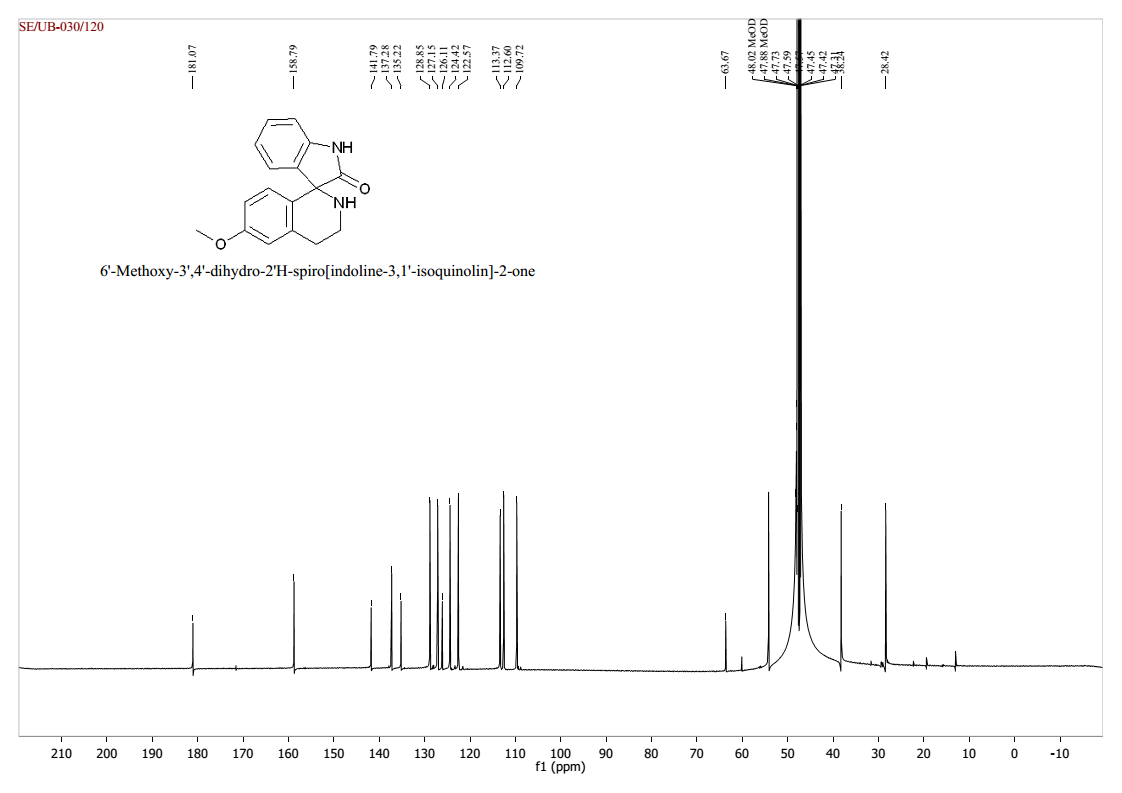
^13^C NMR of 6'-Methoxy-3',4'-dihydro-2'H-spiro[indoline-3,1'-isoquinolin]-2-one (4a)**

**
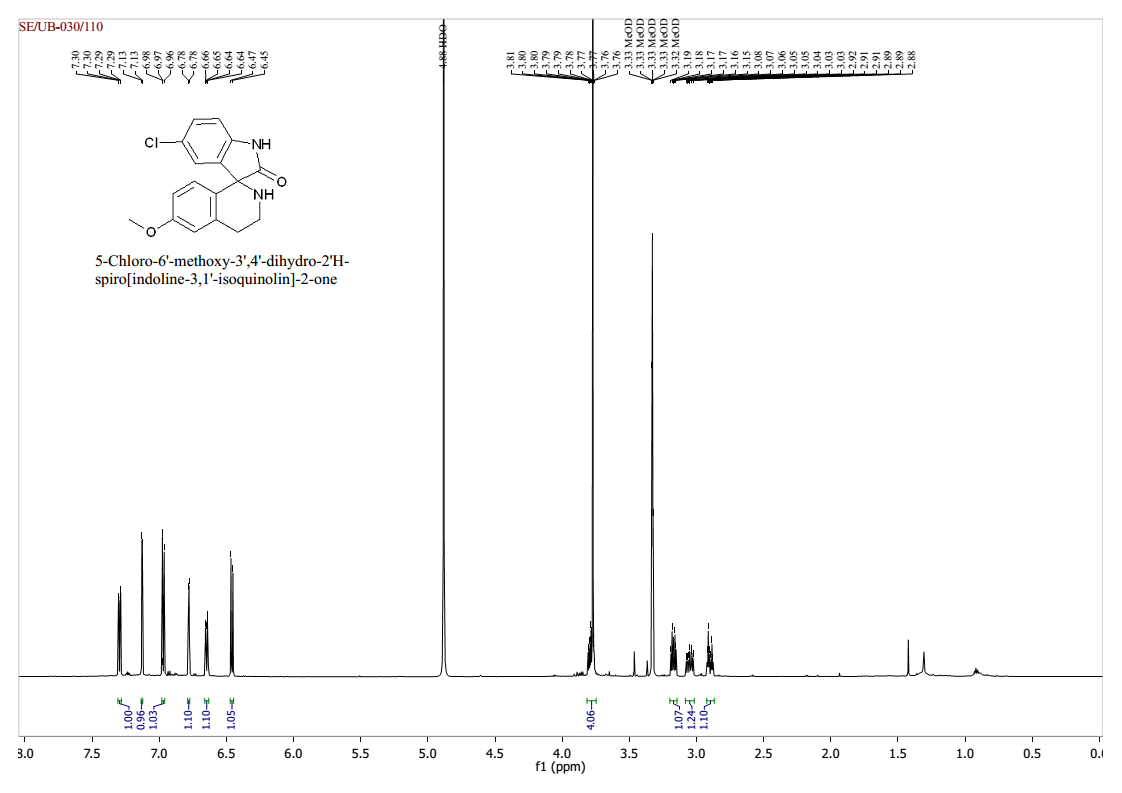
^1^H NMR** **of** **5-Chloro-6'-methoxy-3',4'-dihydro-2'H-spiro[indoline-3,1'-isoquinolin]-2-one (4b)**

**
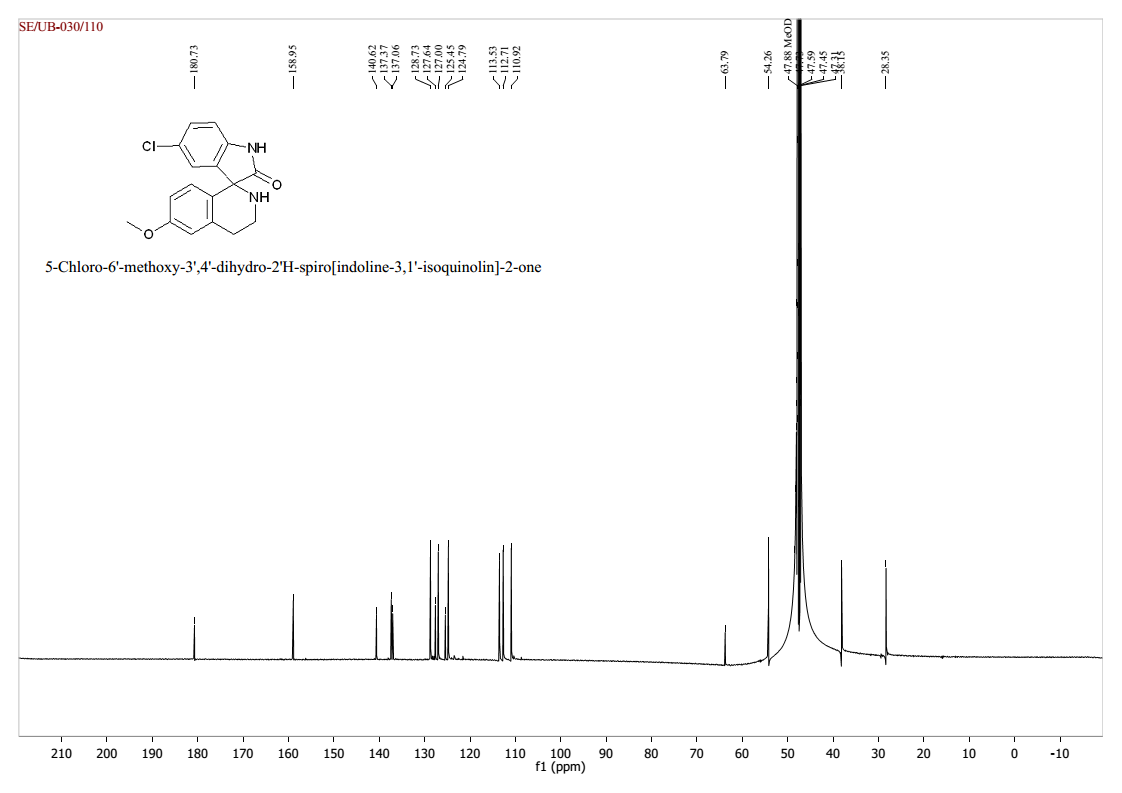
^13^C NMR of 5-Chloro-6'-methoxy-3',4'-dihydro-2'H-spiro[indoline-3,1'-isoquinolin]-2-one (4b)**

**
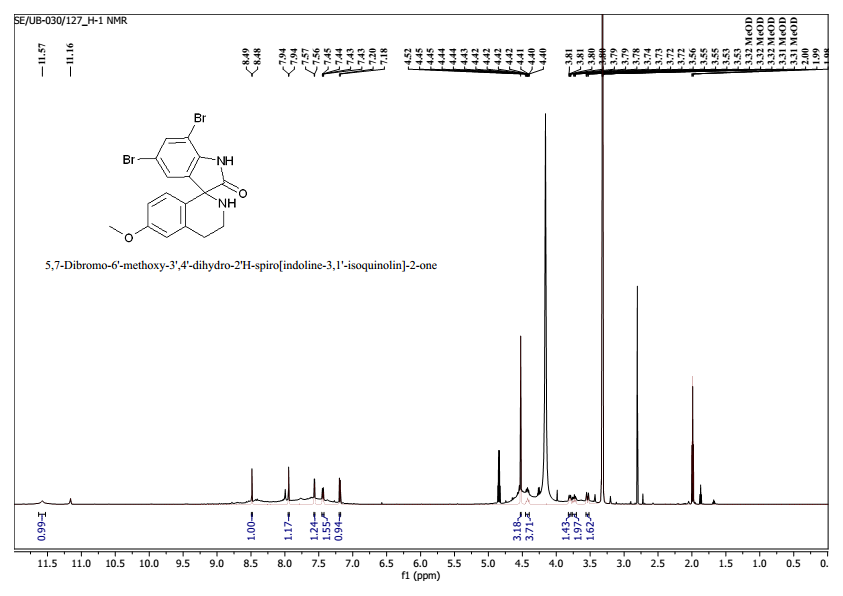
^1^H NMR of 5,7-Dibromo-6'-methoxy-3',4'-dihydro-2'H-spiro[indoline-3,1'-isoquinolin]-2-one (4c)**

**
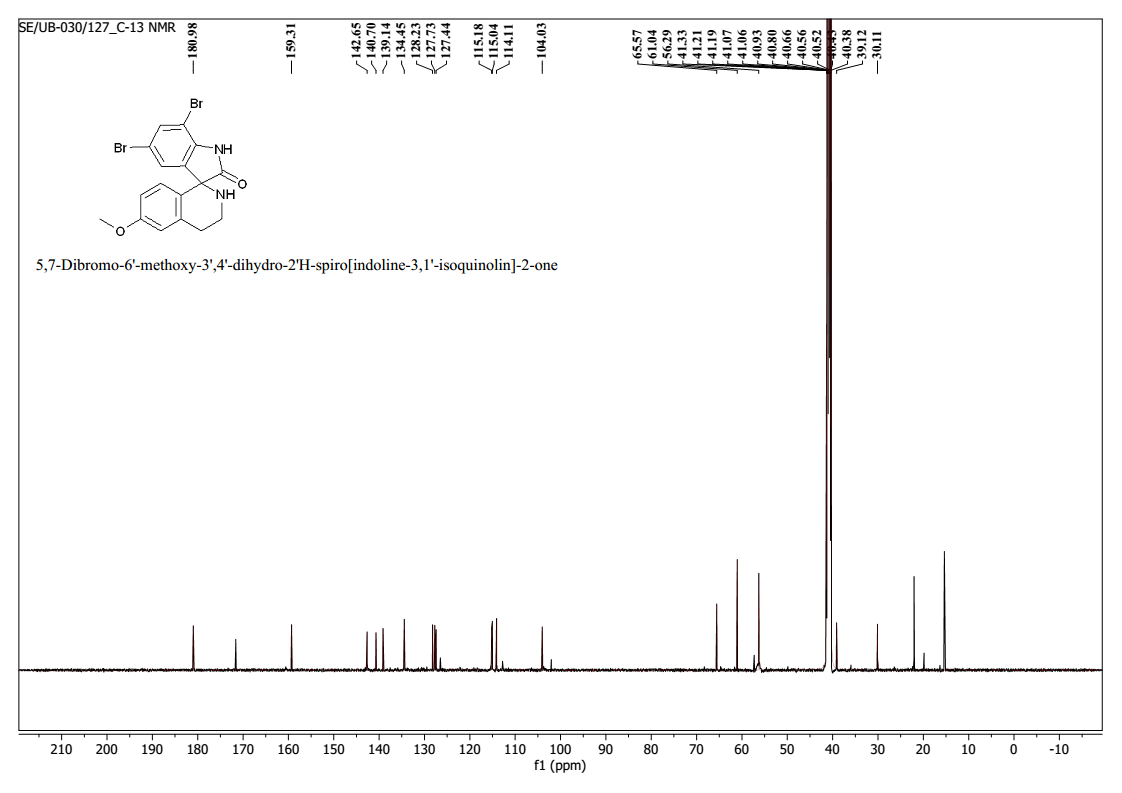
^13^C NMR of 5,7-Dibromo-6'-methoxy-3',4'-dihydro-2'H-spiro[indoline-3,1'-isoquinolin]-2-one (4c)**

**
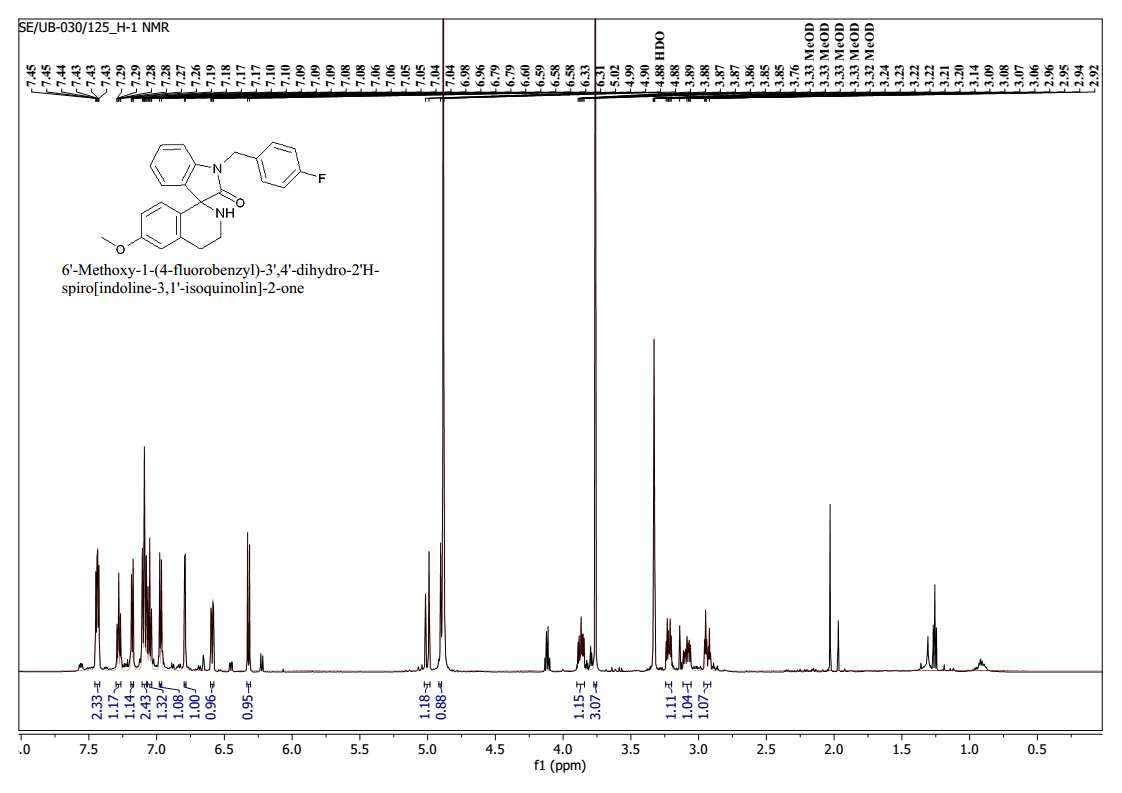
^1^H NMR** **of** **6'-Methoxy-1-(4-fluorobenzyl)-3',4'-dihydro-2'H-spiro[indoline-3,1'-isoquinolin]-2-one (4d)**

**
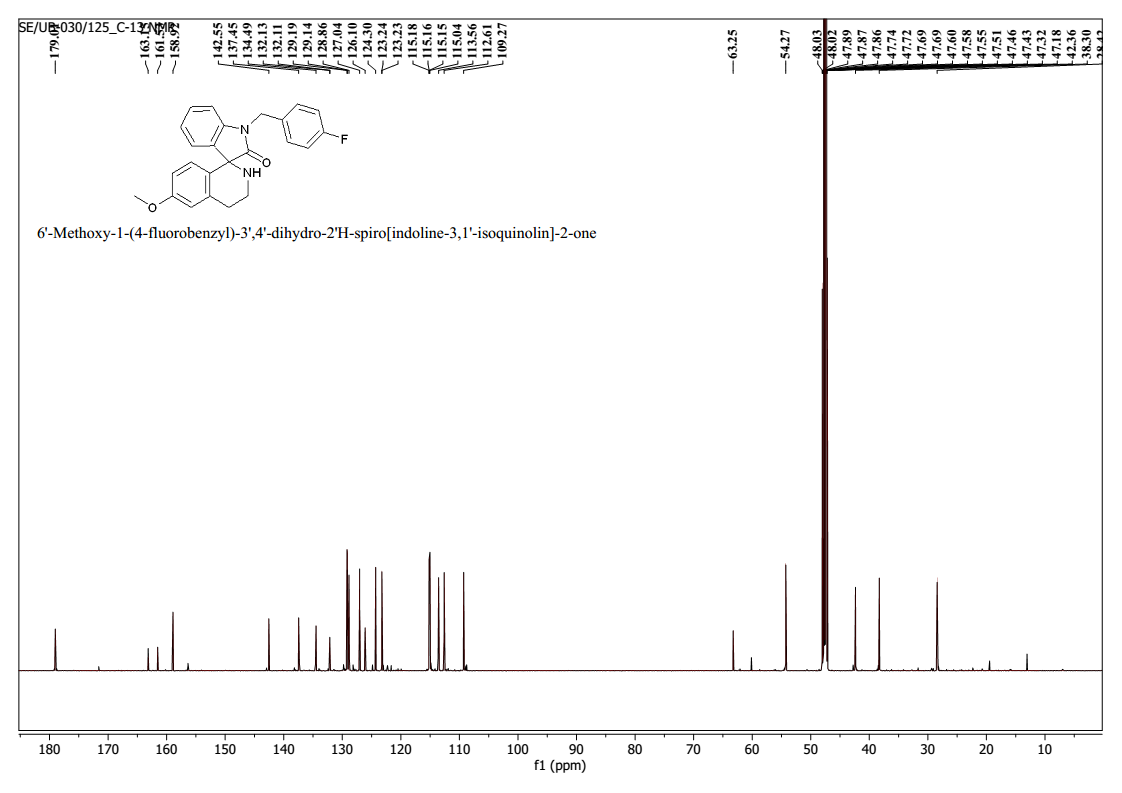
^13^C NMR of 6'-Methoxy-1-(4-fluorobenzyl)-3',4'-dihydro-2'H-spiro[indoline-3,1'-isoquinolin]-2-one (4d)**

**
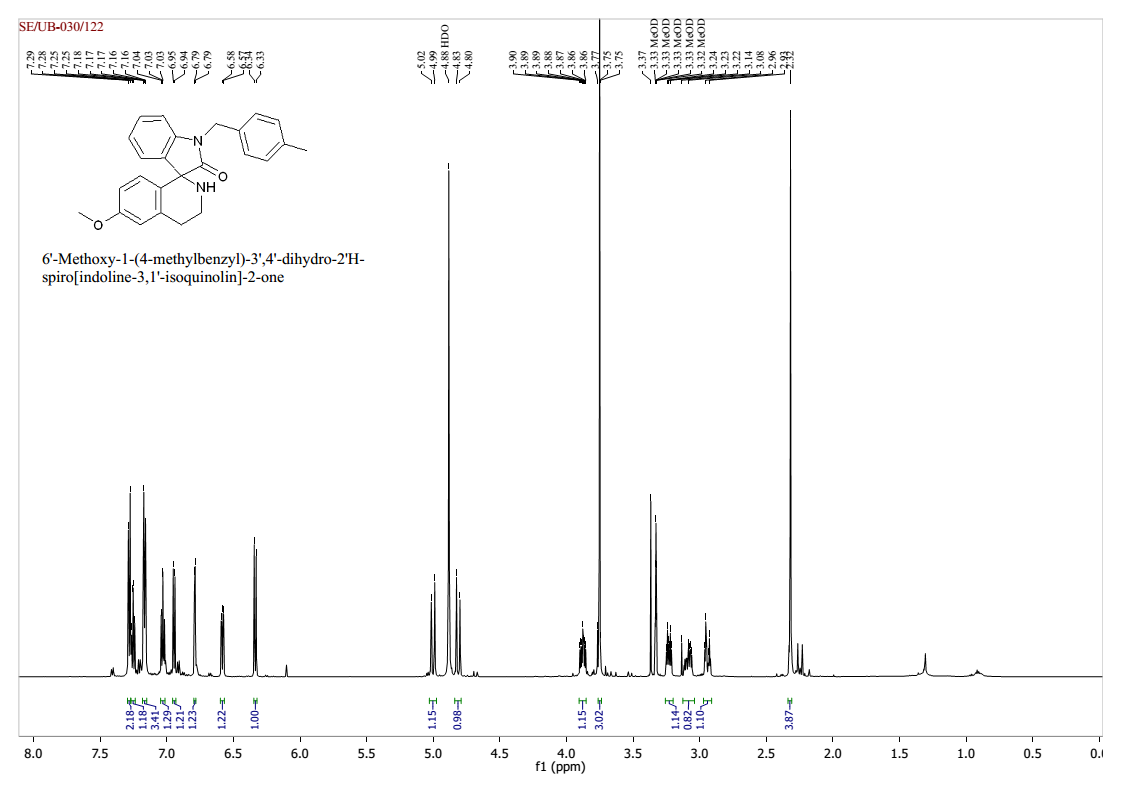
^1^H NMR** **of** **6'-Methoxy-1-(4-methylbenzyl)-3',4'-dihydro-2'H-spiro[indoline-3,1'-isoquinolin]-2-one (4e)**

**
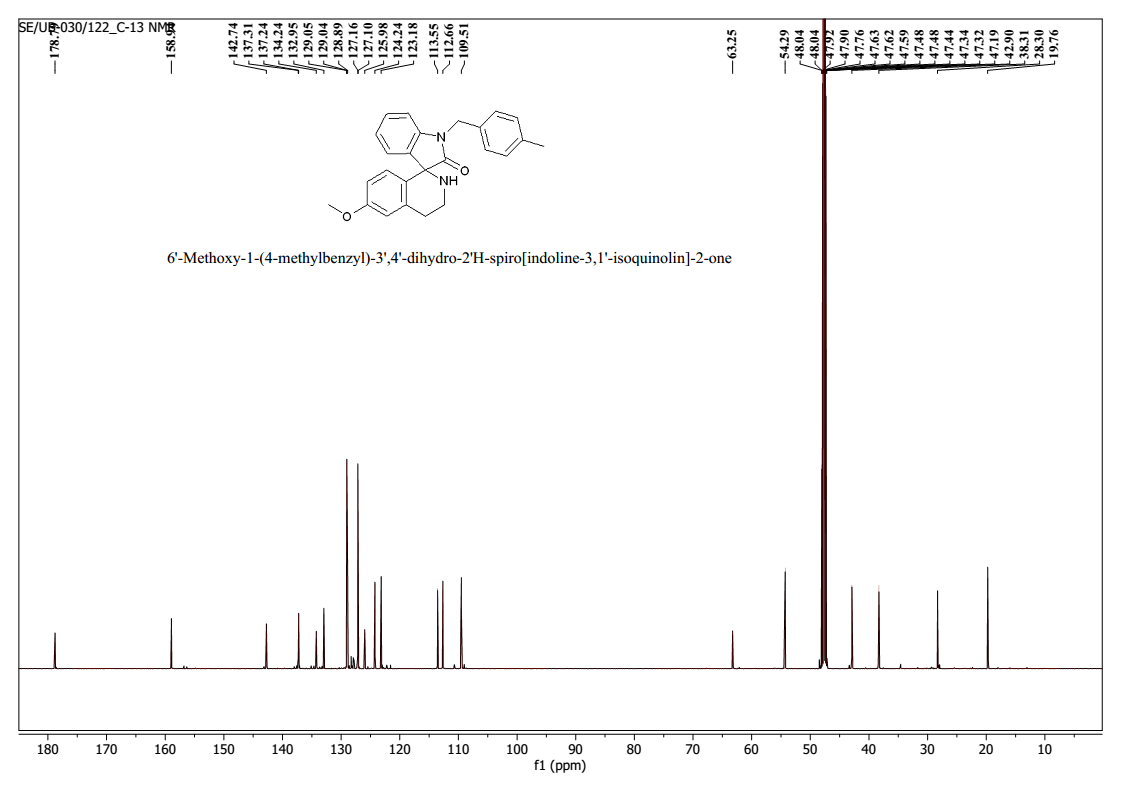
^13^C NMR of 6'-Methoxy-1-(4-methylbenzyl)-3',4'-dihydro-2'H-spiro[indoline-3,1'-isoquinolin]-2-one (4e)**

**
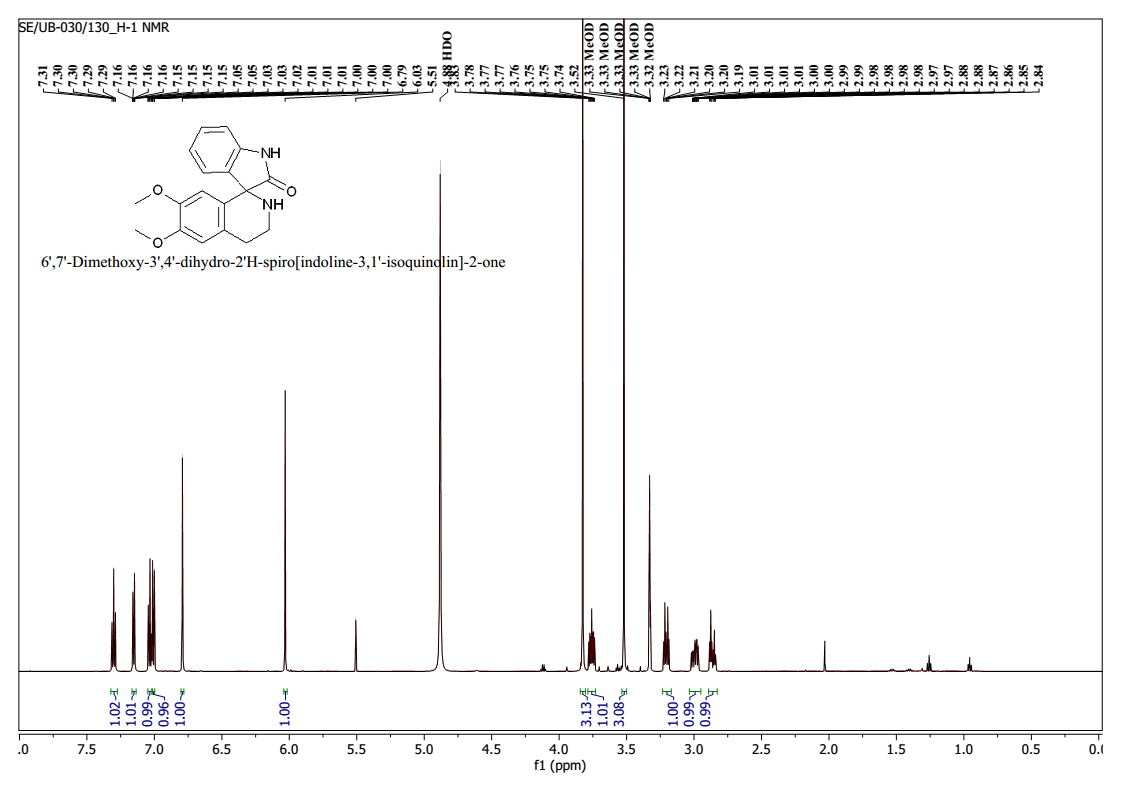
^1^H NMR** **of** **6',7'-Dimethoxy-3',4'-dihydro-2'H-spiro[indoline-3,1'-isoquinolin]-2-one (5a)**

**
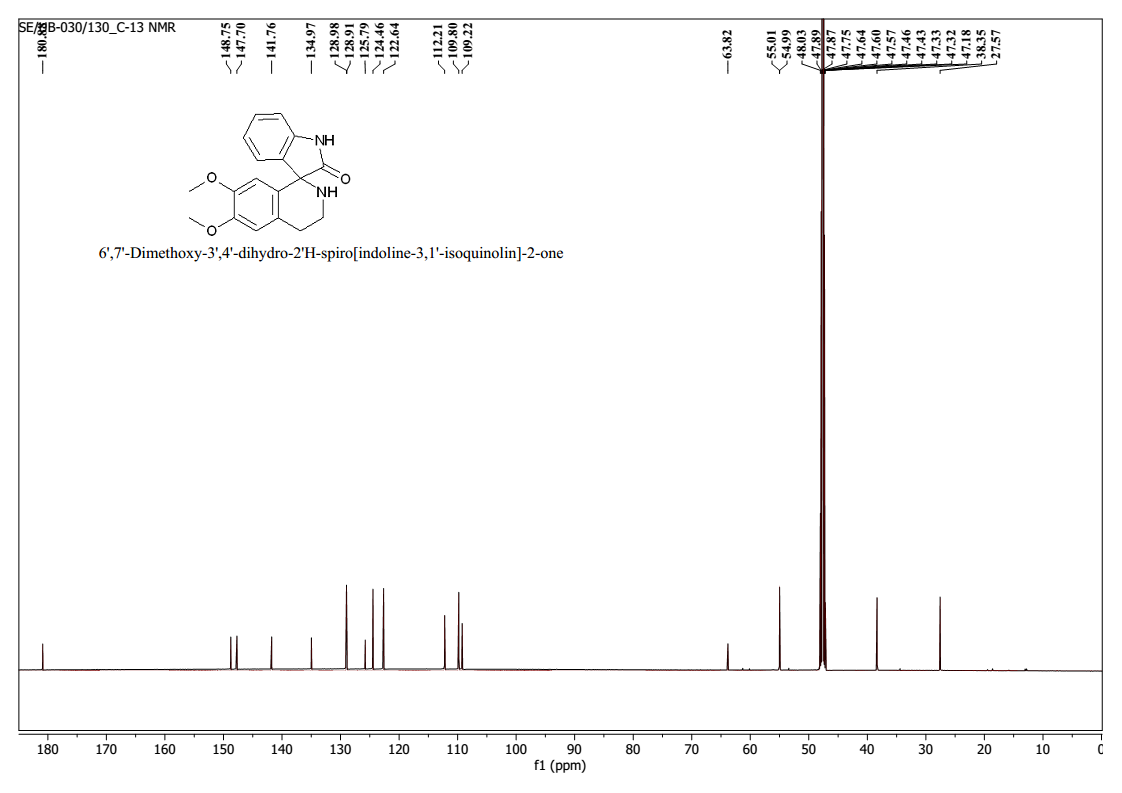
^13^C NMR of 6',7'-Dimethoxy-3',4'-dihydro-2'H-spiro[indoline-3,1'-isoquinolin]-2-one (5a)**

**
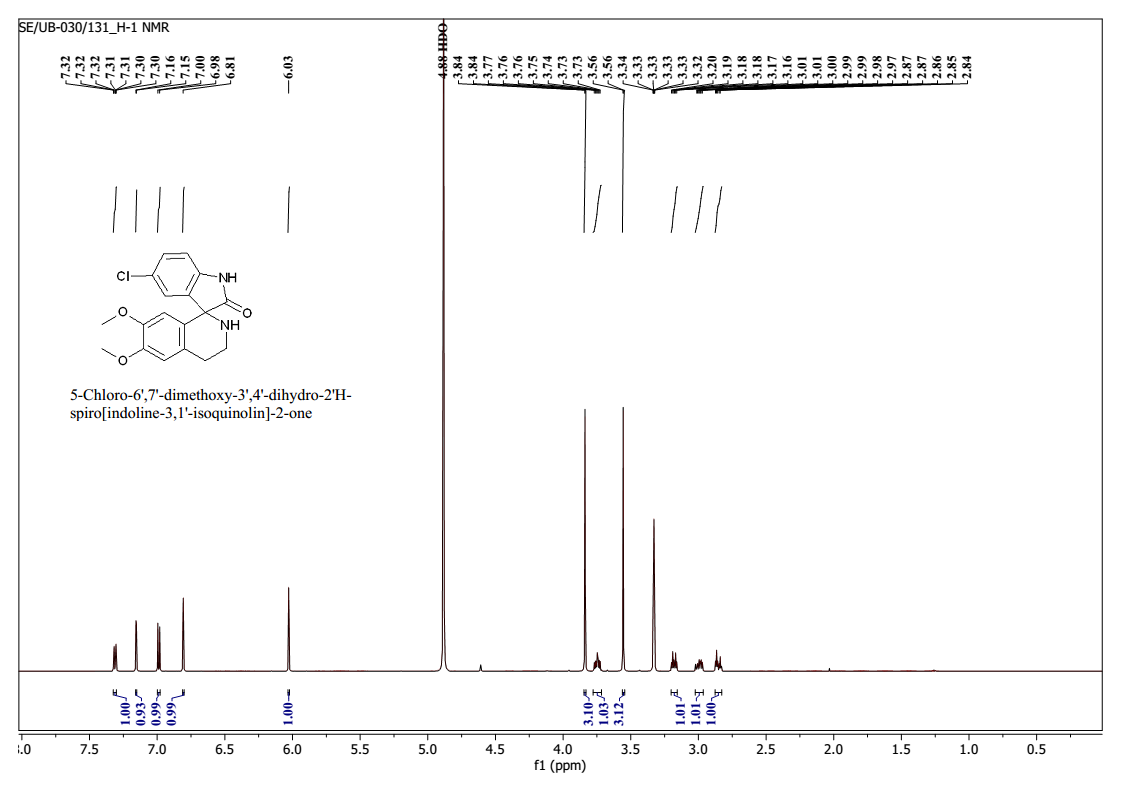
^1^H NMR** **of** **5-Chloro-6',7'-dimethoxy-3',4'-dihydro-2'H-spiro[indoline-3,1'-isoquinolin]-2-one (5b)**

**
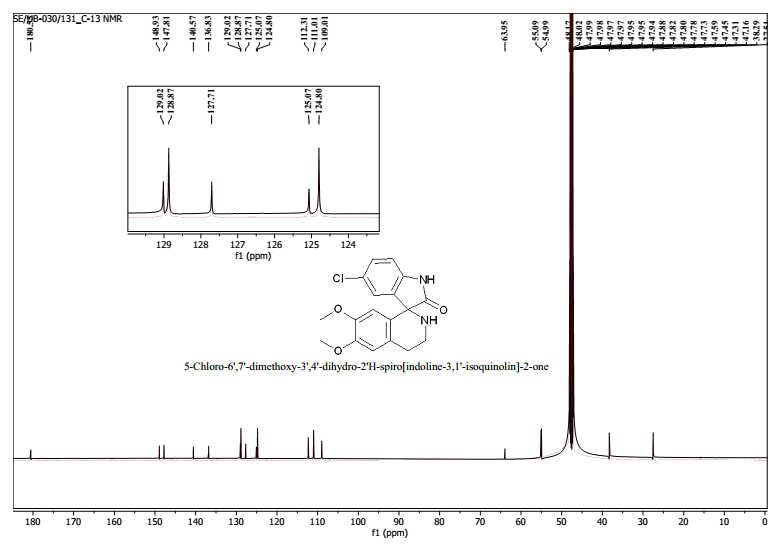
^13^C NMR of 5-Chloro-6',7'-dimethoxy-3',4'-dihydro-2'H-spiro[indoline-3,1'-isoquinolin]-2-one (5b)**

**
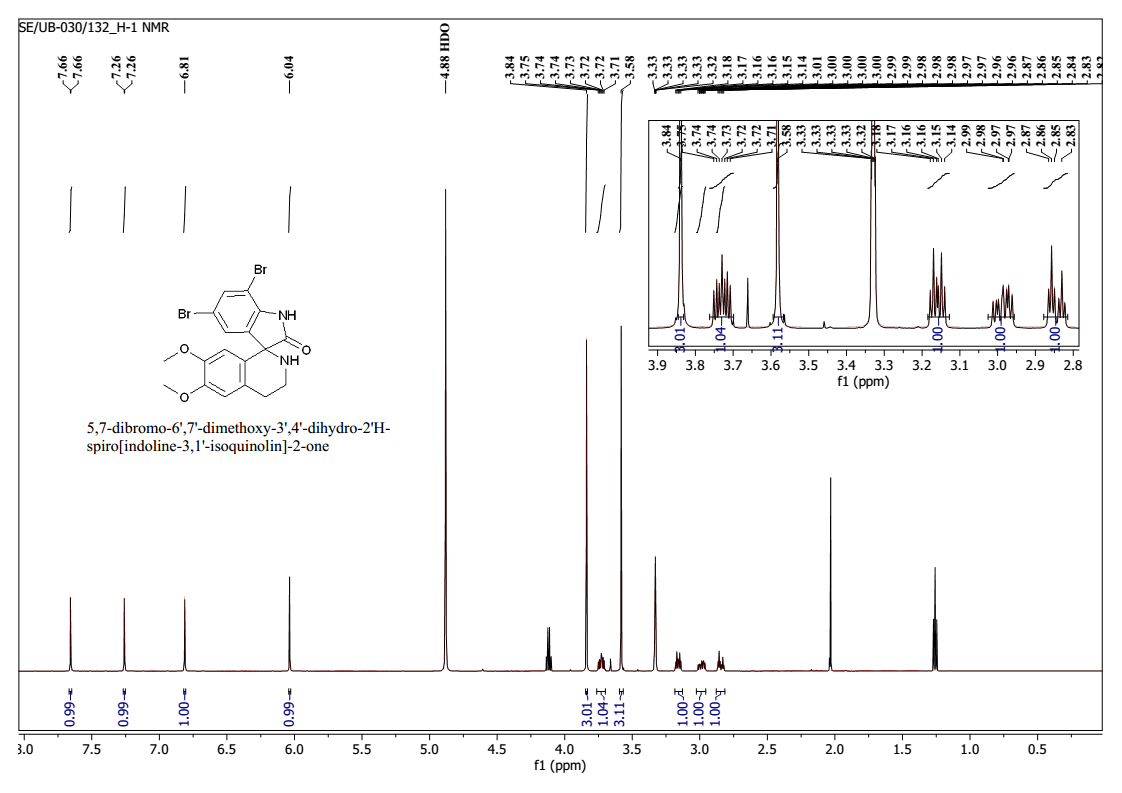
^1^H NMR** **of** **5,7-dibromo-6',7'-dimethoxy-3',4'-dihydro-2'H-spiro[indoline-3,1'-isoquinolin]-2-one (5c)**

**
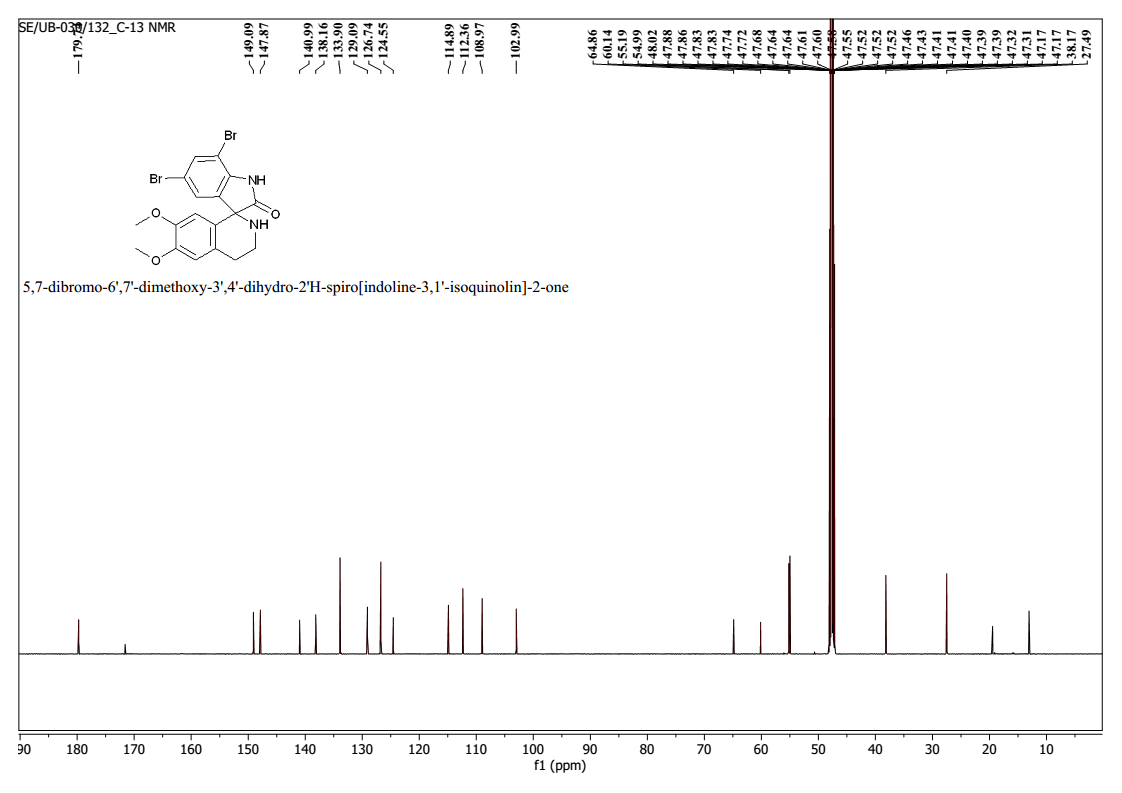
^13^C NMR of 5,7-dibromo-6',7'-dimethoxy-3',4'-dihydro-2'H-spiro[indoline-3,1'-isoquinolin]-2-one (5c)**

**
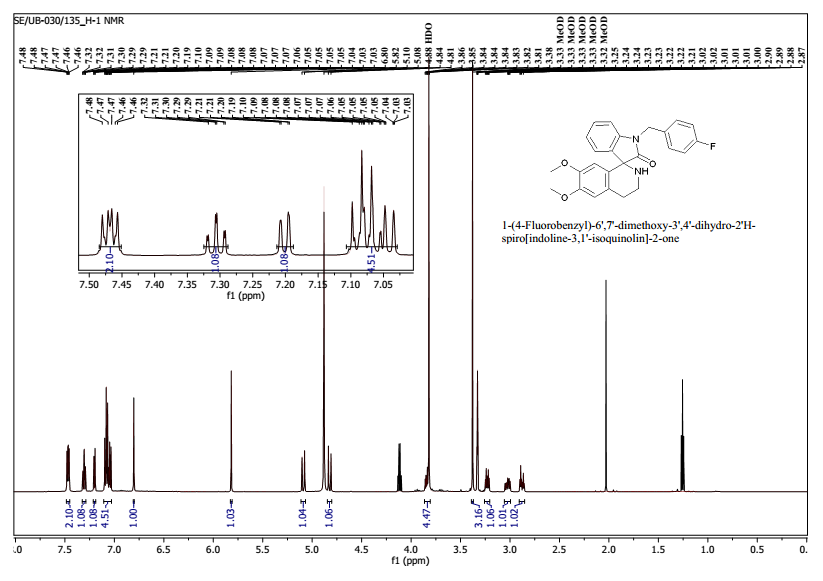
^1^H NMR** **of** **1-(4-Fluorobenzyl)-6',7'-dimethoxy-3',4'-dihydro-2'H-spiro[indoline-3,1'-isoquinolin]-2-one (5d)**

**
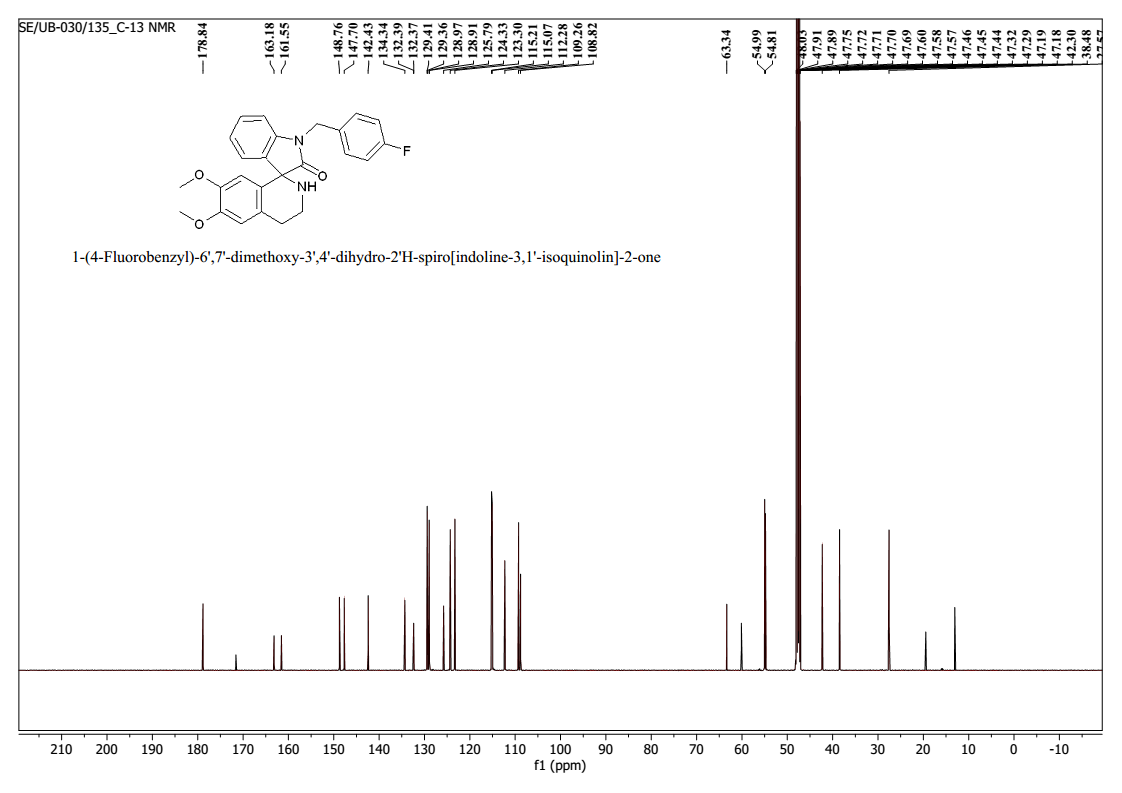
^13^C NMR of 1-(4-Fluorobenzyl)-6',7'-dimethoxy-3',4'-dihydro-2'H-spiro[indoline-3,1'-isoquinolin]-2-one (5d)**

**
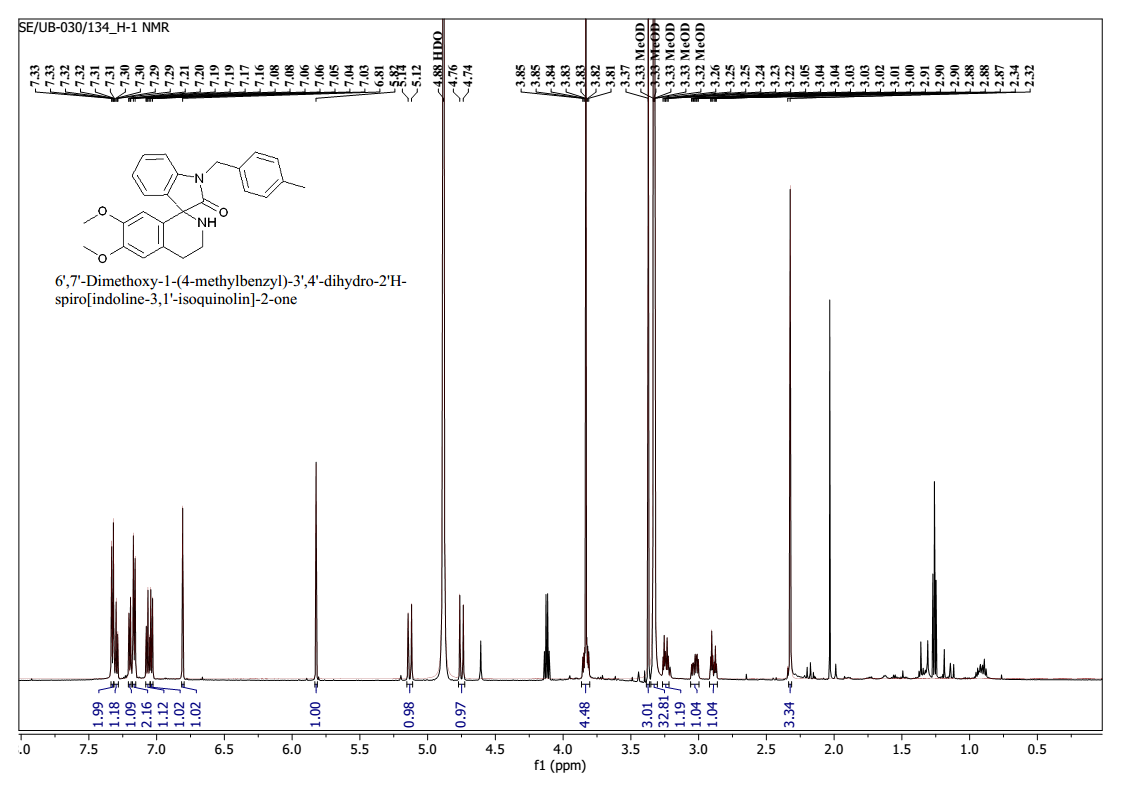
^1^H NMR** **of** **6',7'-Dimethoxy-1-(4-methylbenzyl)-3',4'-dihydro-2'H-spiro[indoline-3,1'-isoquinolin]-2-one (5e)**

**
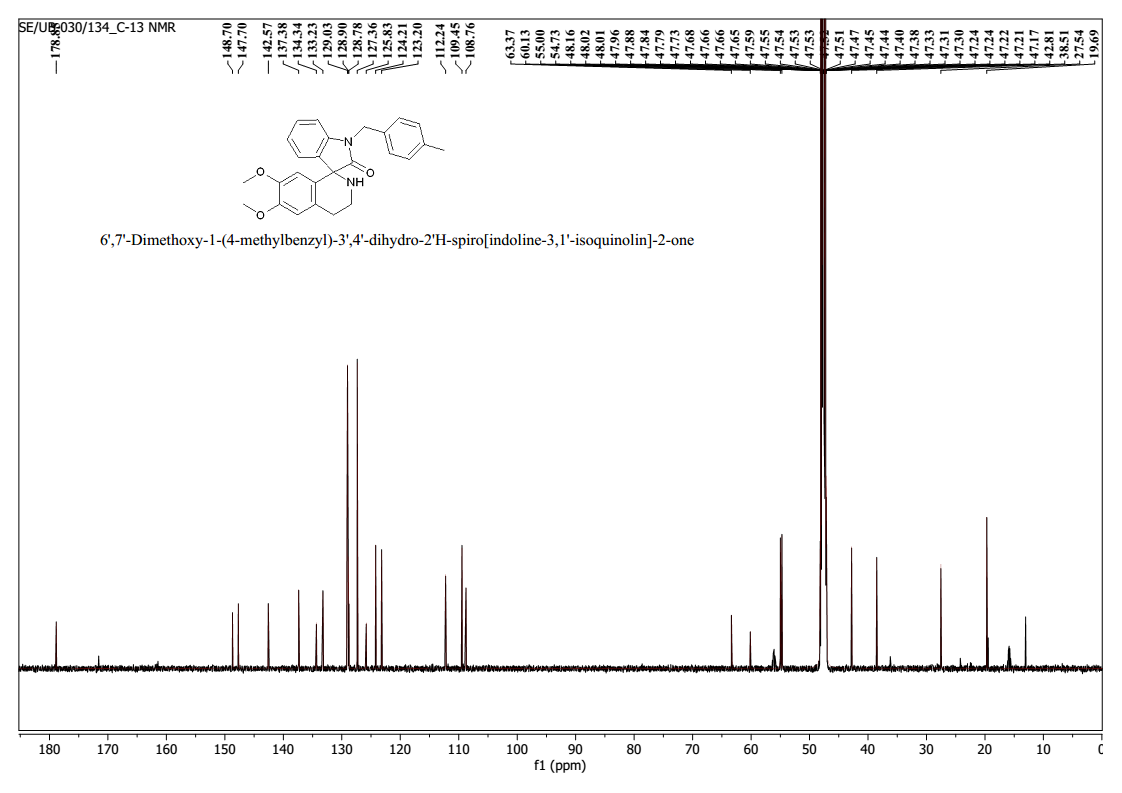
^13^C NMR of 6',7'-Dimethoxy-1-(4-methylbenzyl)-3',4'-dihydro-2'H-spiro[indoline-3,1'-isoquinolin]-2-one (5e)**

**
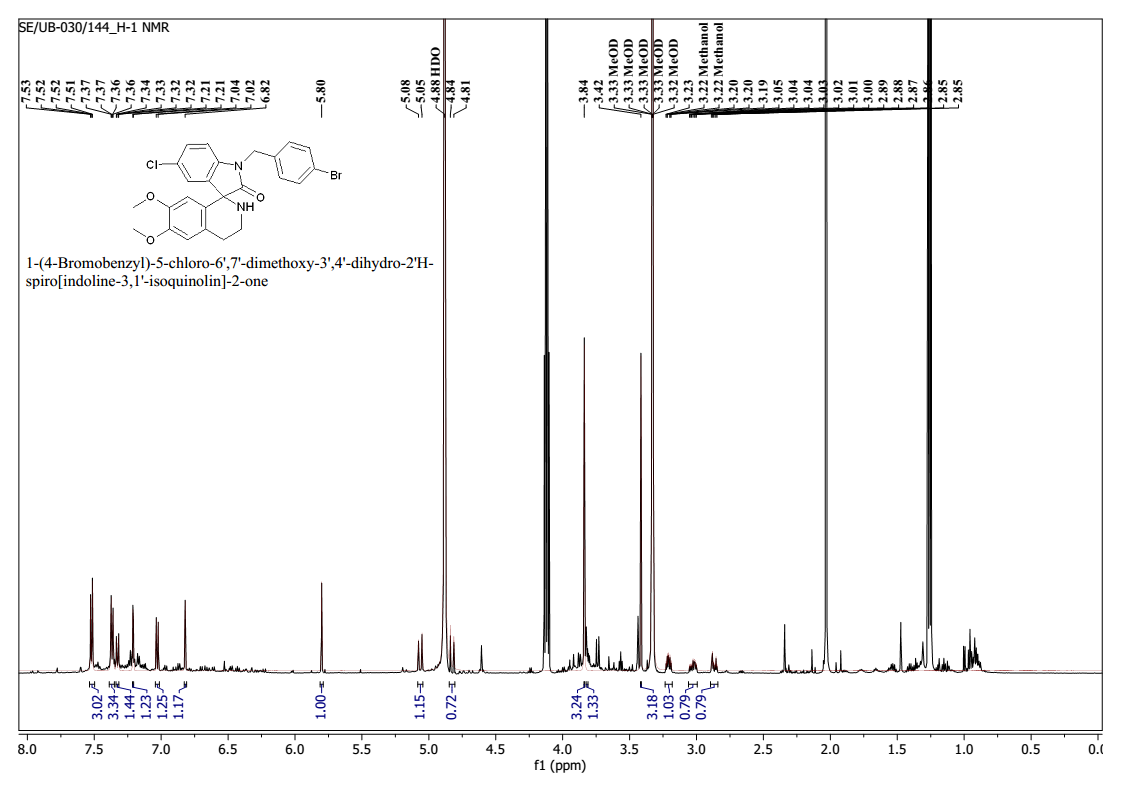
^1^H NMR** **of** **1-(4-Bromobenzyl)-5-chloro-6',7'-dimethoxy-3',4'-dihydro-2'H-spiro[indoline-3,1'-isoquinolin]-2-one (5f)**

**
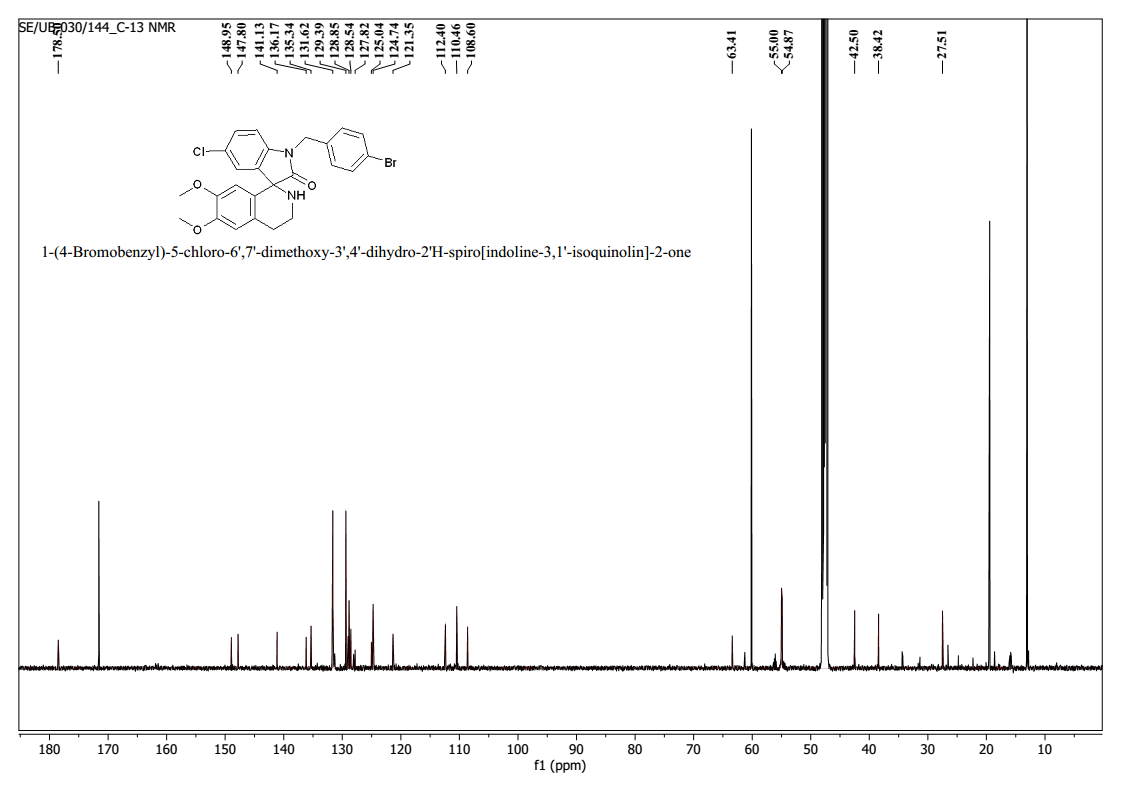
^13^C NMR of 1-(4-Bromobenzyl)-5-chloro-6',7'-dimethoxy-3',4'-dihydro-2'H-spiro[indoline-3,1'-isoquinolin]-2-one (5f)**

**
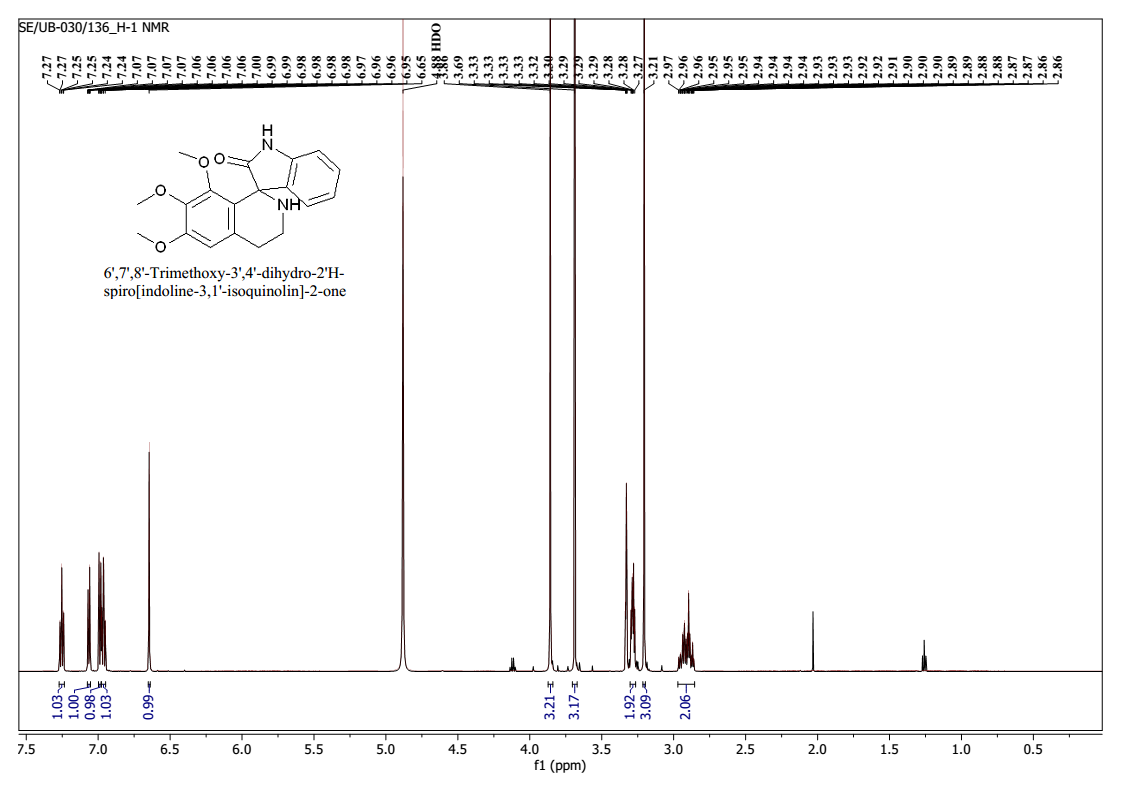
^1^H NMR** **of** **6',7',8'-Trimethoxy-3',4'-dihydro-2'H-spiro[indoline-3,1'-isoquinolin]-2-one (6a)**

**
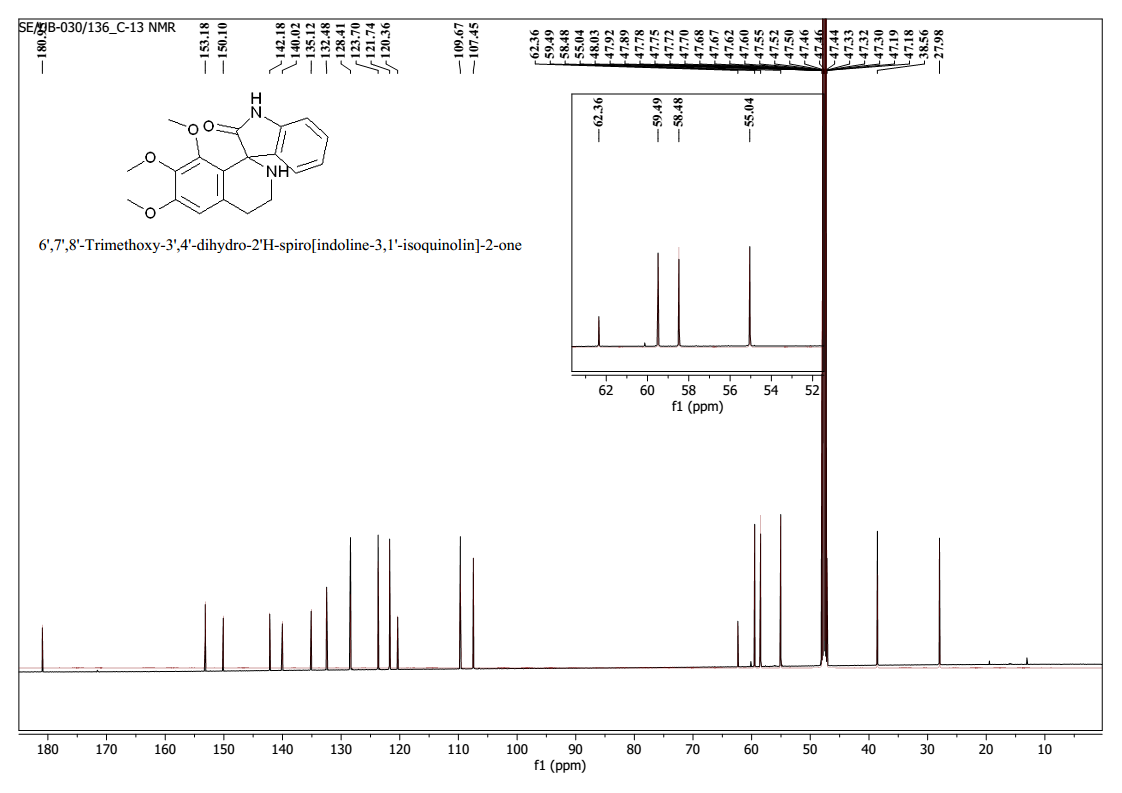
^13^C NMR of 6',7',8'-Trimethoxy-3',4'-dihydro-2'H-spiro[indoline-3,1'-isoquinolin]-2-one (6a)**

**
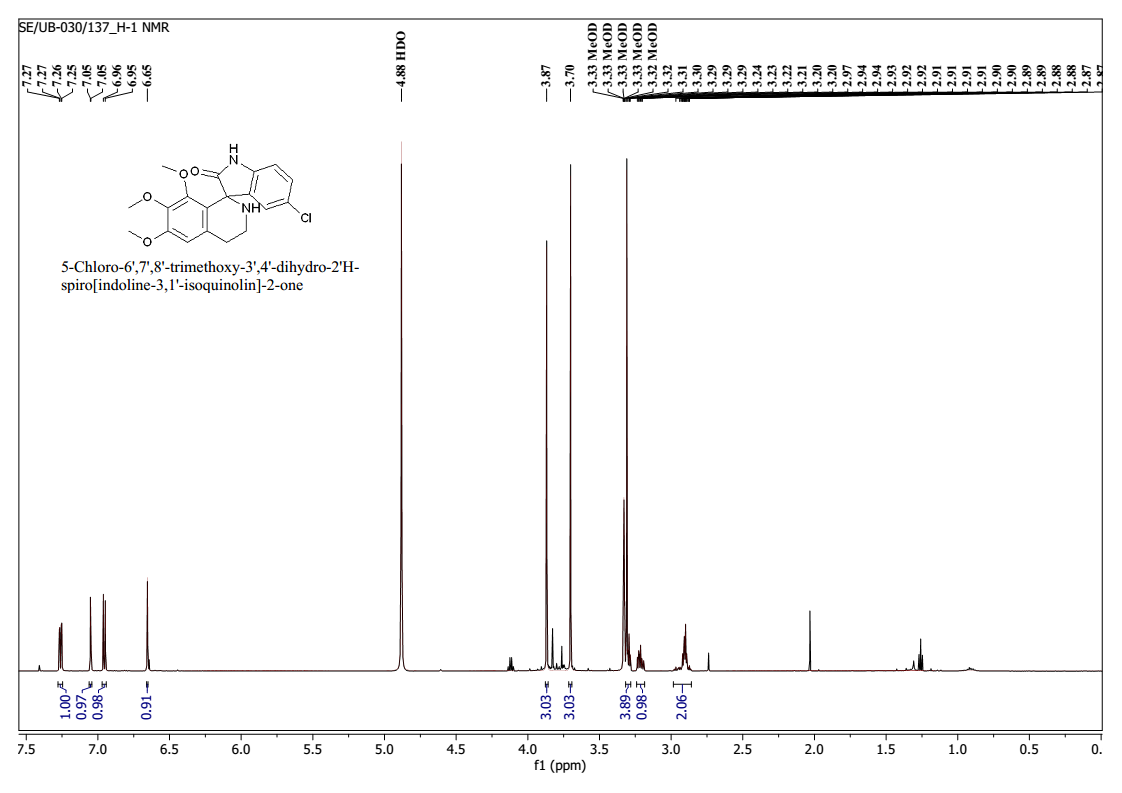
^1^H NMR** **of** **5-Chloro-6',7',8'-trimethoxy-3',4'-dihydro-2'H-spiro[indoline-3,1'-isoquinolin]-2-one (6b)**

**
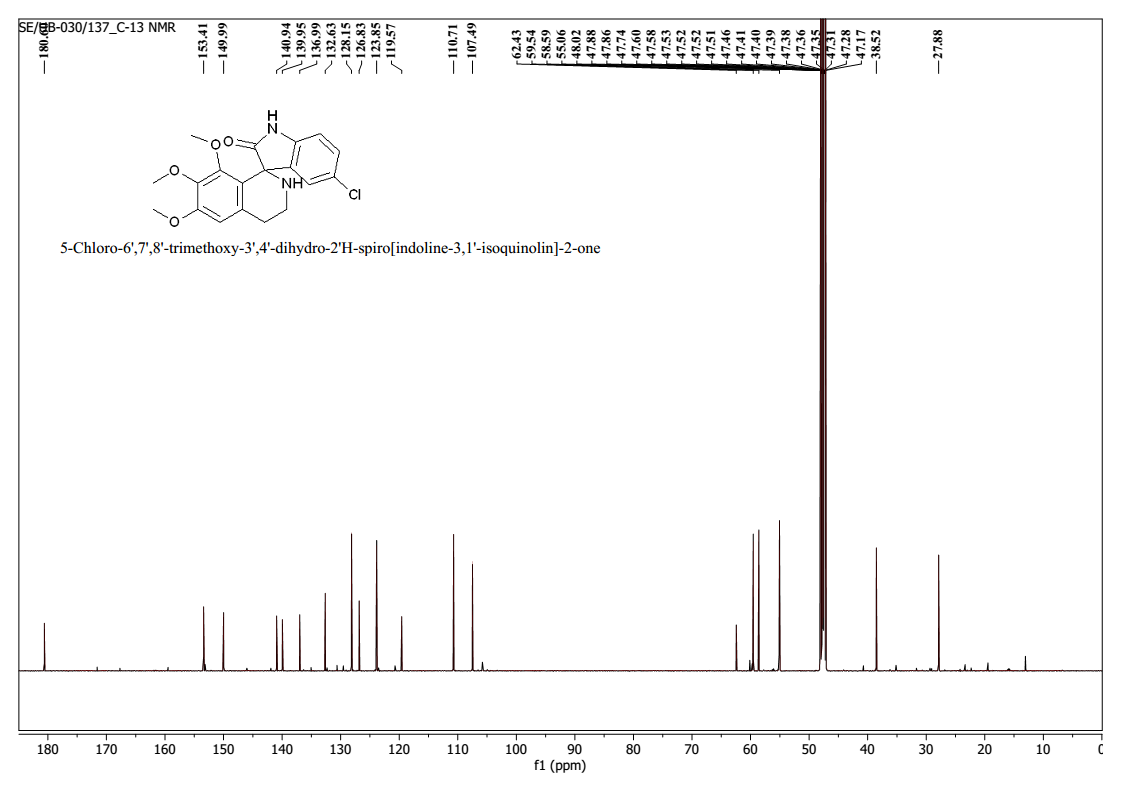
^13^C NMR of 5-Chloro-6',7',8'-trimethoxy-3',4'-dihydro-2'H-spiro[indoline-3,1'-isoquinolin]-2-one (6b)**

**
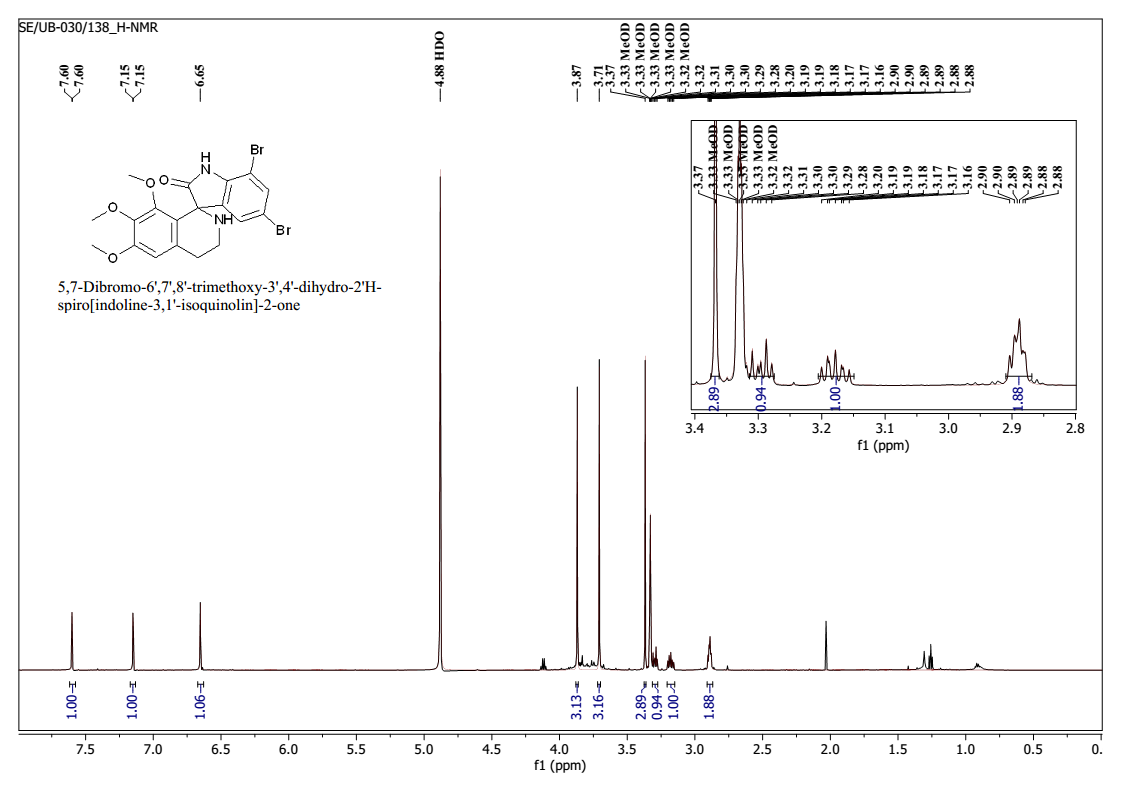
^1^H NMR** **of** **5,7-Dibromo-6',7',8'-trimethoxy-3',4'-dihydro-2'H-spiro[indoline-3,1'-isoquinolin]-2-one (6c)**

**
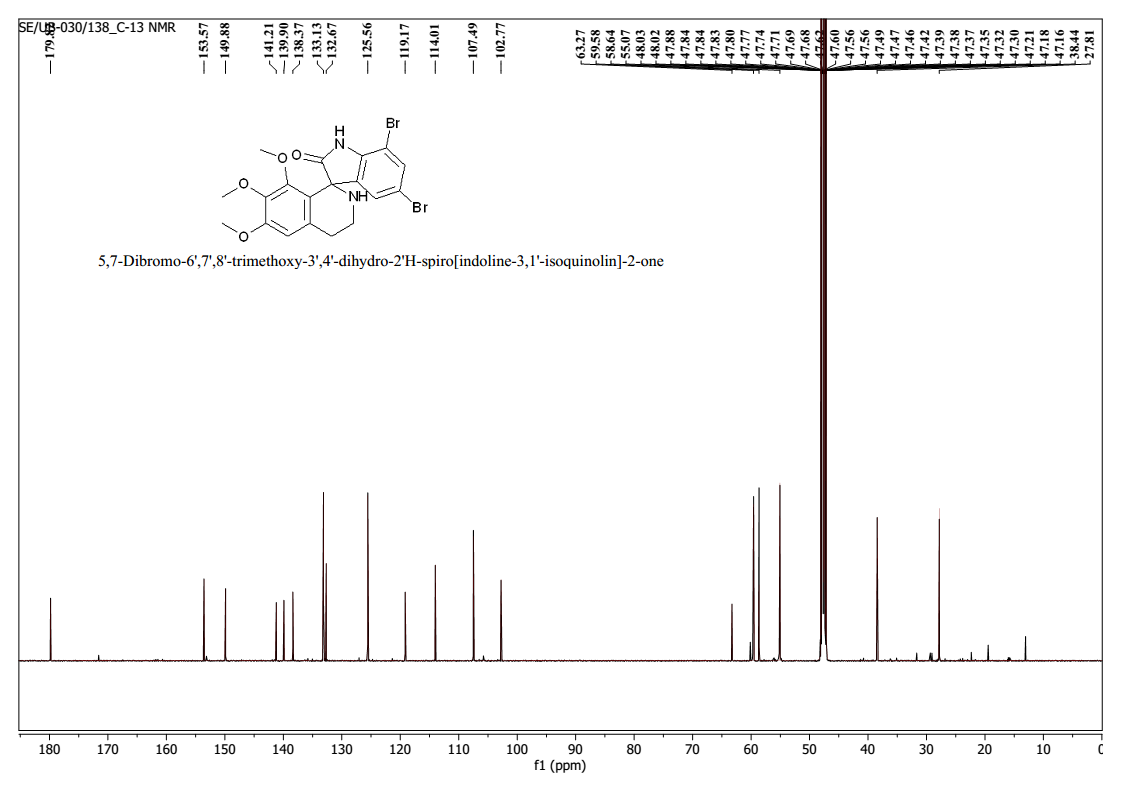
^13^C NMR of 5,7-Dibromo-6',7',8'-trimethoxy-3',4'-dihydro-2'H-spiro[indoline-3,1'-isoquinolin]-2-one (6c)**

**
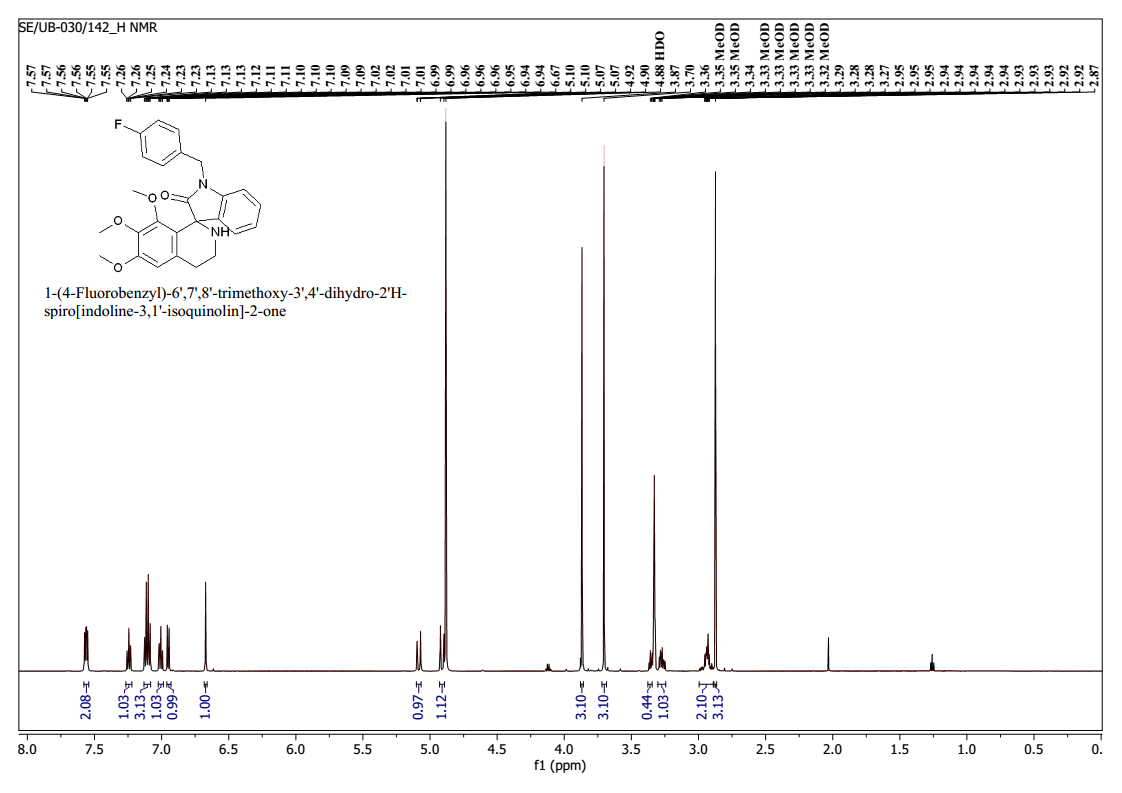
^1^H NMR** **of** **1-(4-Fluorobenzyl)-6',7',8'-trimethoxy-3',4'-dihydro-2'H-spiro[indoline-3,1'-isoquinolin]-2-one (6d)**

**
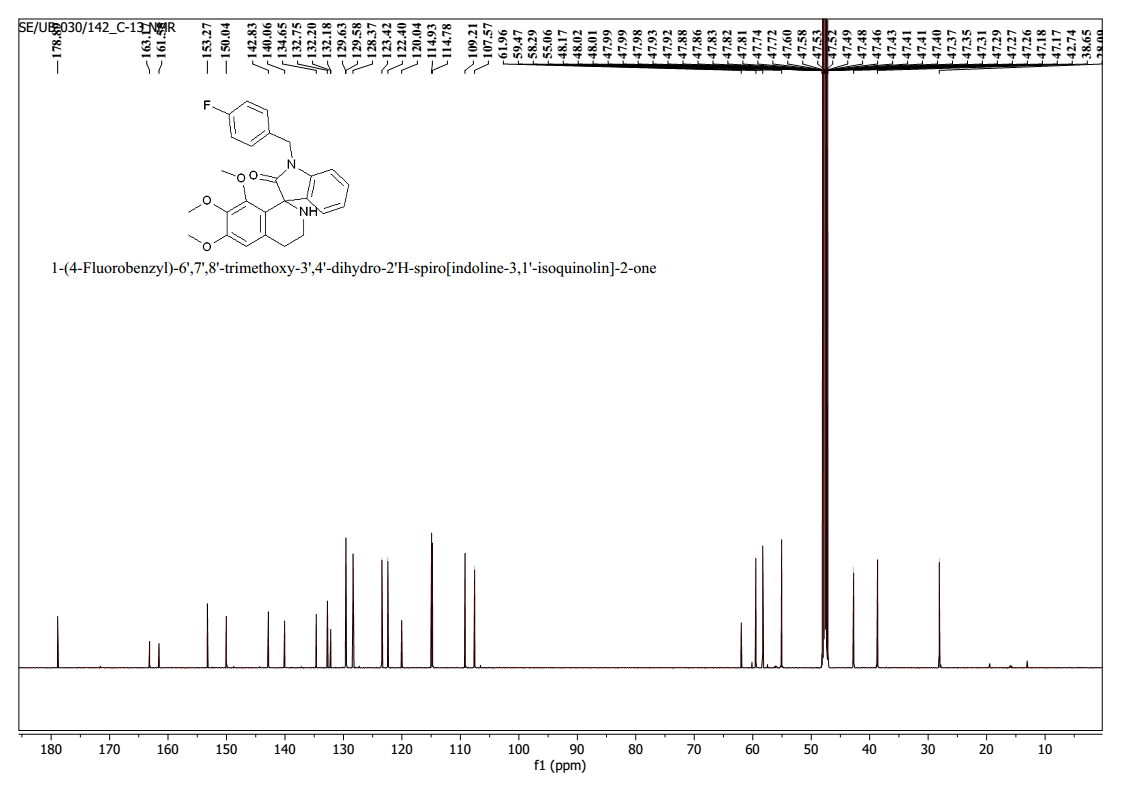
^13^C NMR of 1-(4-Fluorobenzyl)-6',7',8'-trimethoxy-3',4'-dihydro-2'H-spiro[indoline-3,1'-isoquinolin]-2-one (6d)**

**
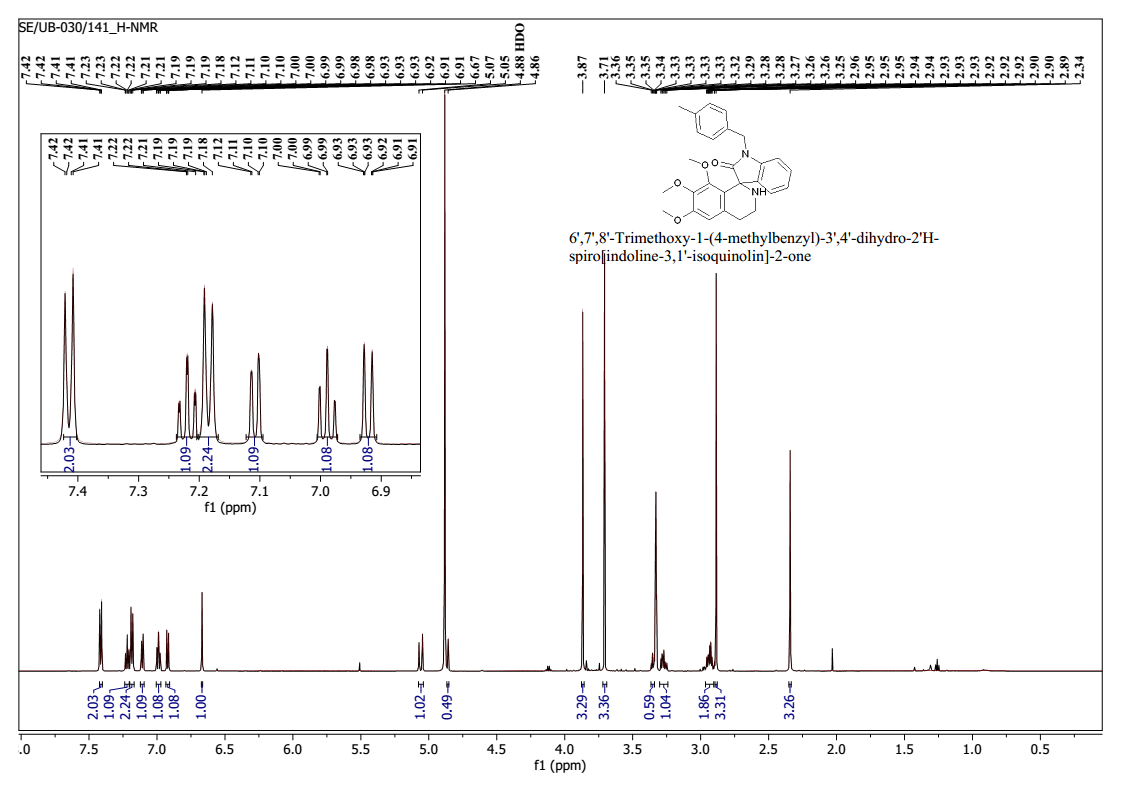
^1^H NMR** **of** **6',7',8'-Trimethoxy-1-(4-methylbenzyl)-3',4'-dihydro-2'H-spiro[indoline-3,1'-isoquinolin]-2-one (6e)**

**
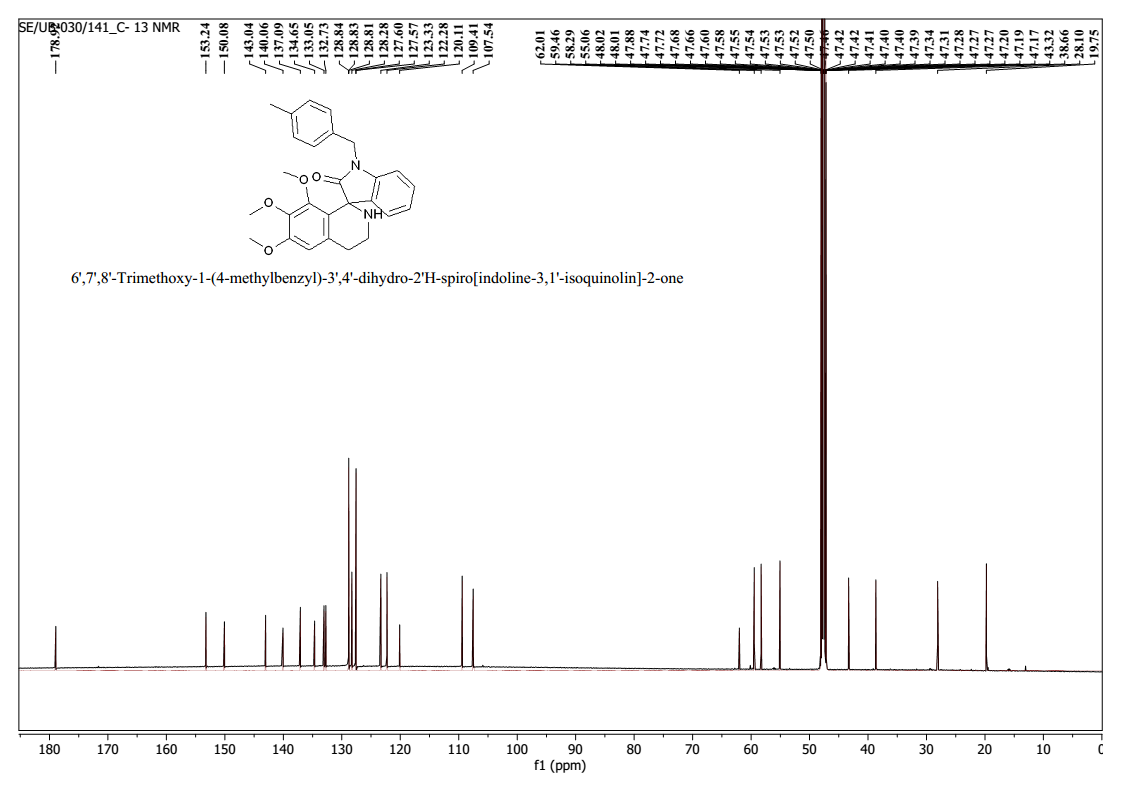
**

**^13^C NMR of 6',7',8'-Trimethoxy-1-(4-methylbenzyl)-3',4'-dihydro-2'H-spiro[indoline-3,1'-isoquinolin]-2-one (6e)**

**^LC-MS SPECTRA OF ALL COMPOUNDS^**


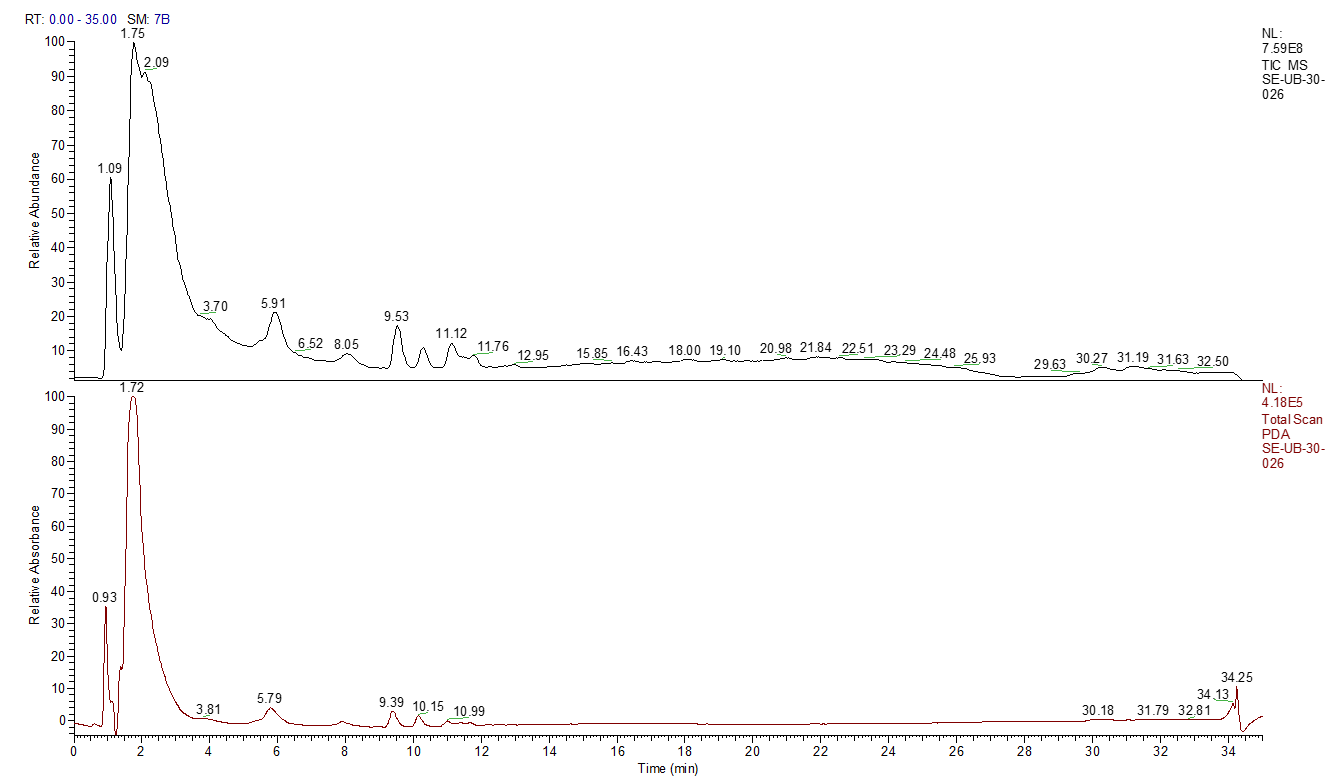


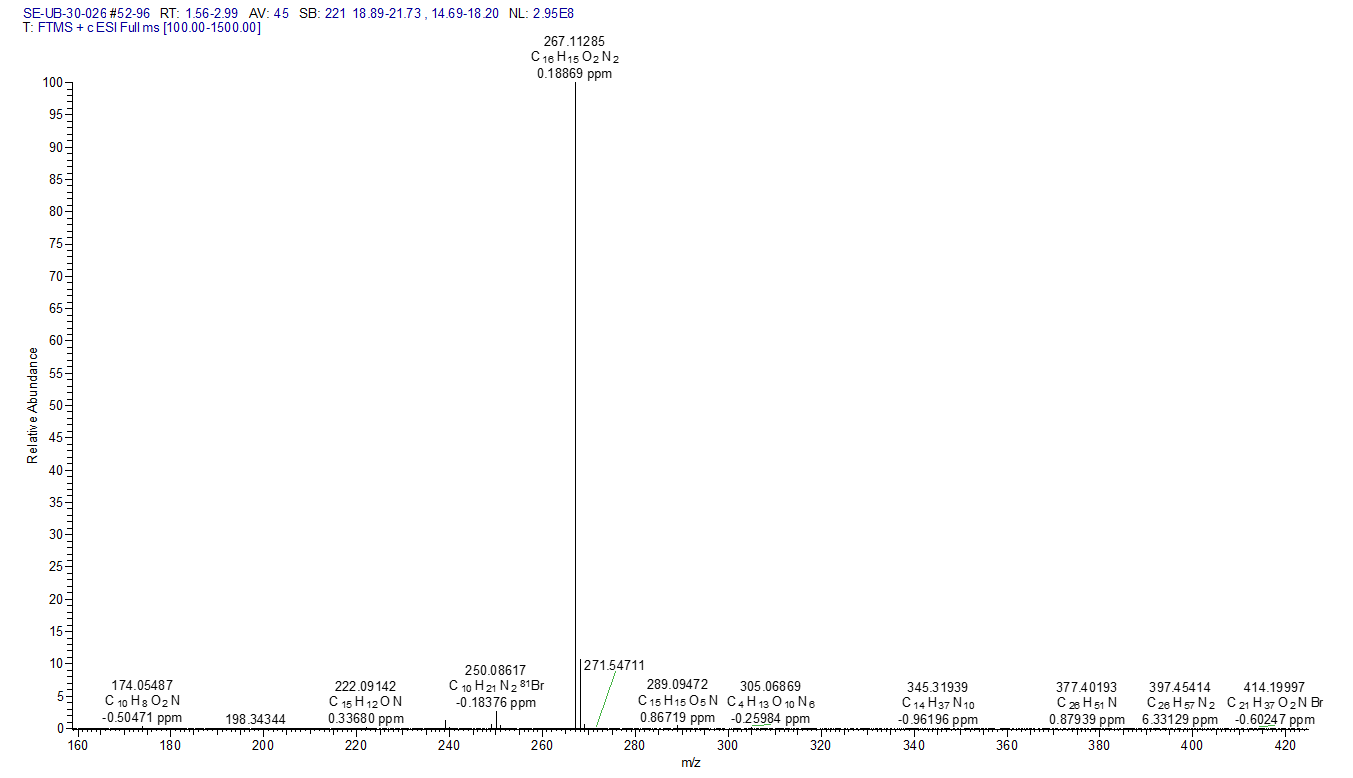


**LC-MS spectra of 8'-Hydroxy-3',4'-dihydro-2'H-spiro[indoline-3,1'-isoquinolin]-2-one (1a)**


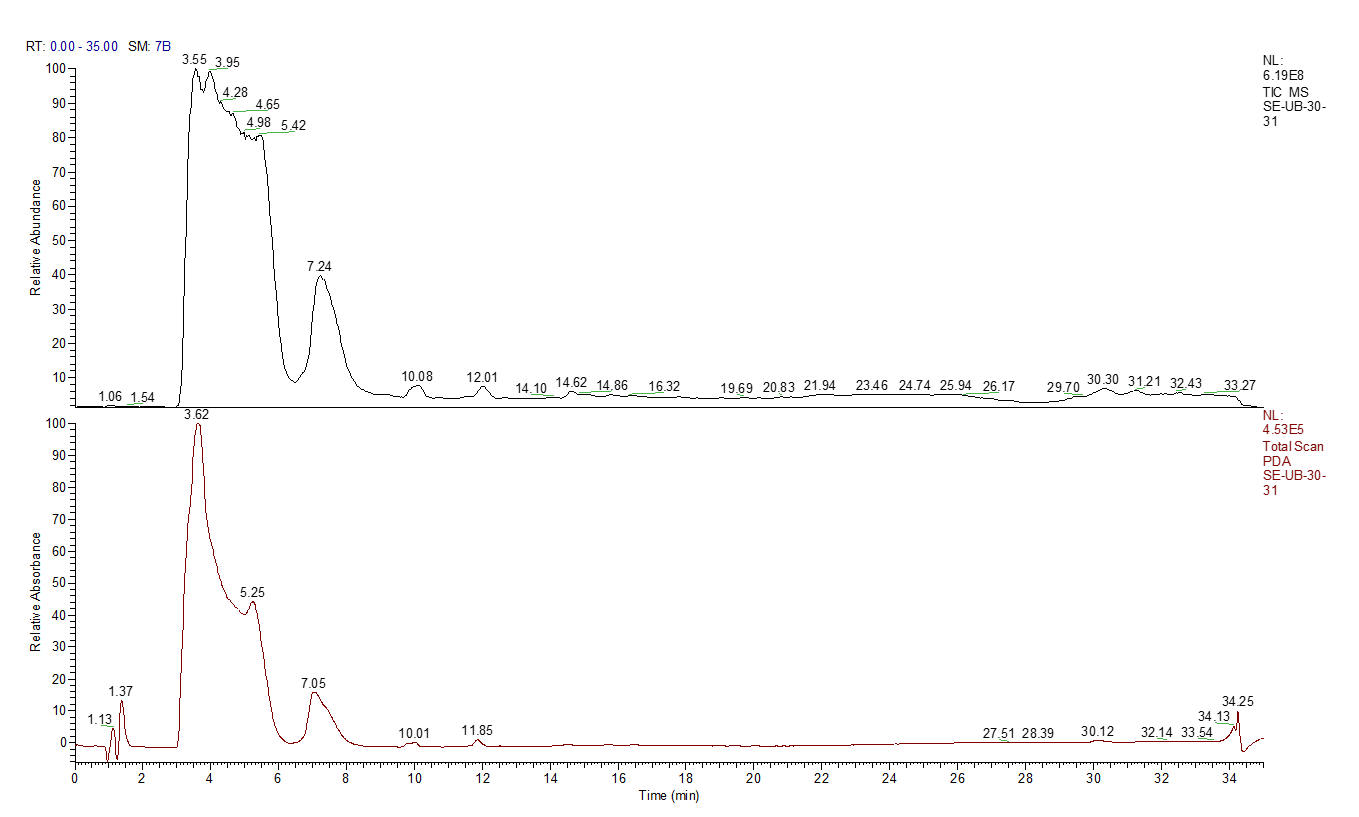


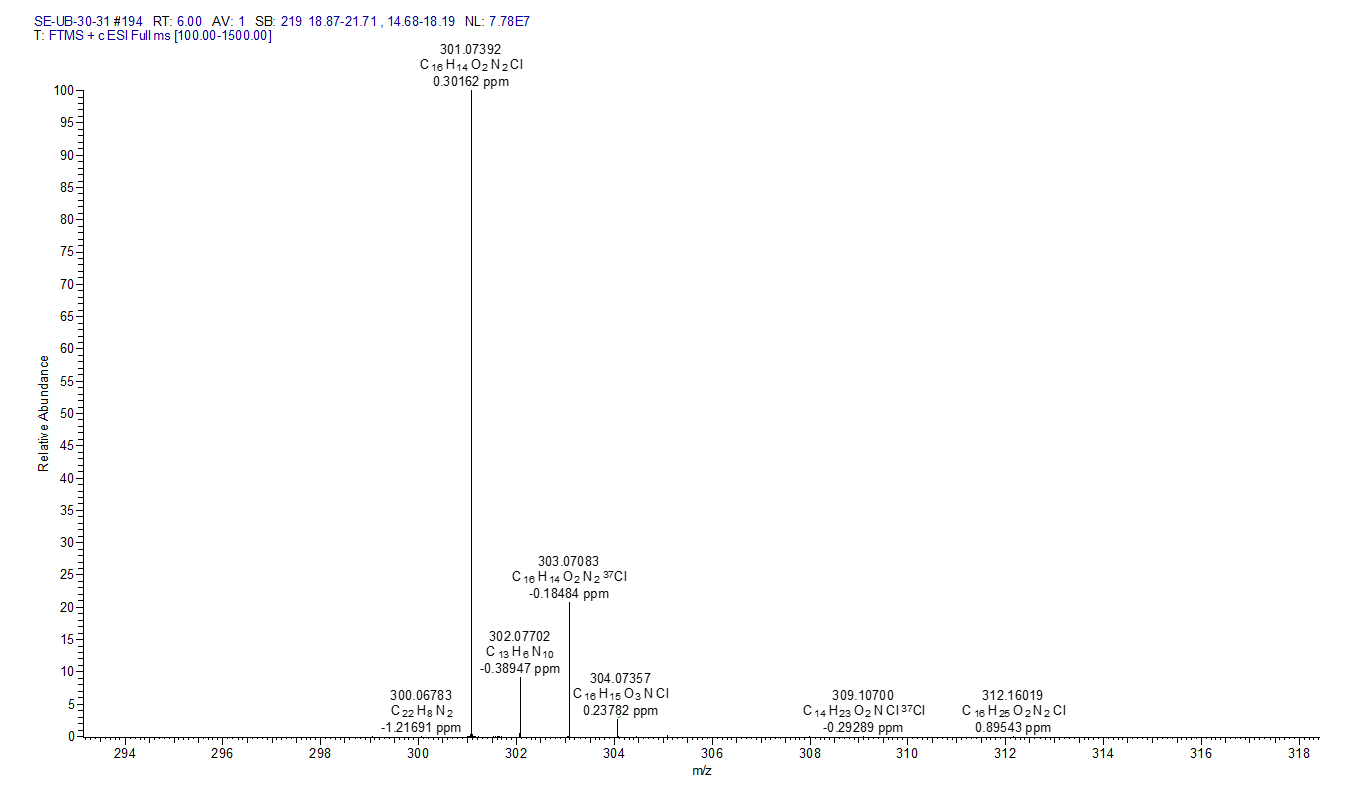


**LC-MS spectra of 5-Chloro-6'-hydroxy-3',4'-dihydro-2'H-spiro[indoline-3,1'-isoquinolin]-2-one (2b)**


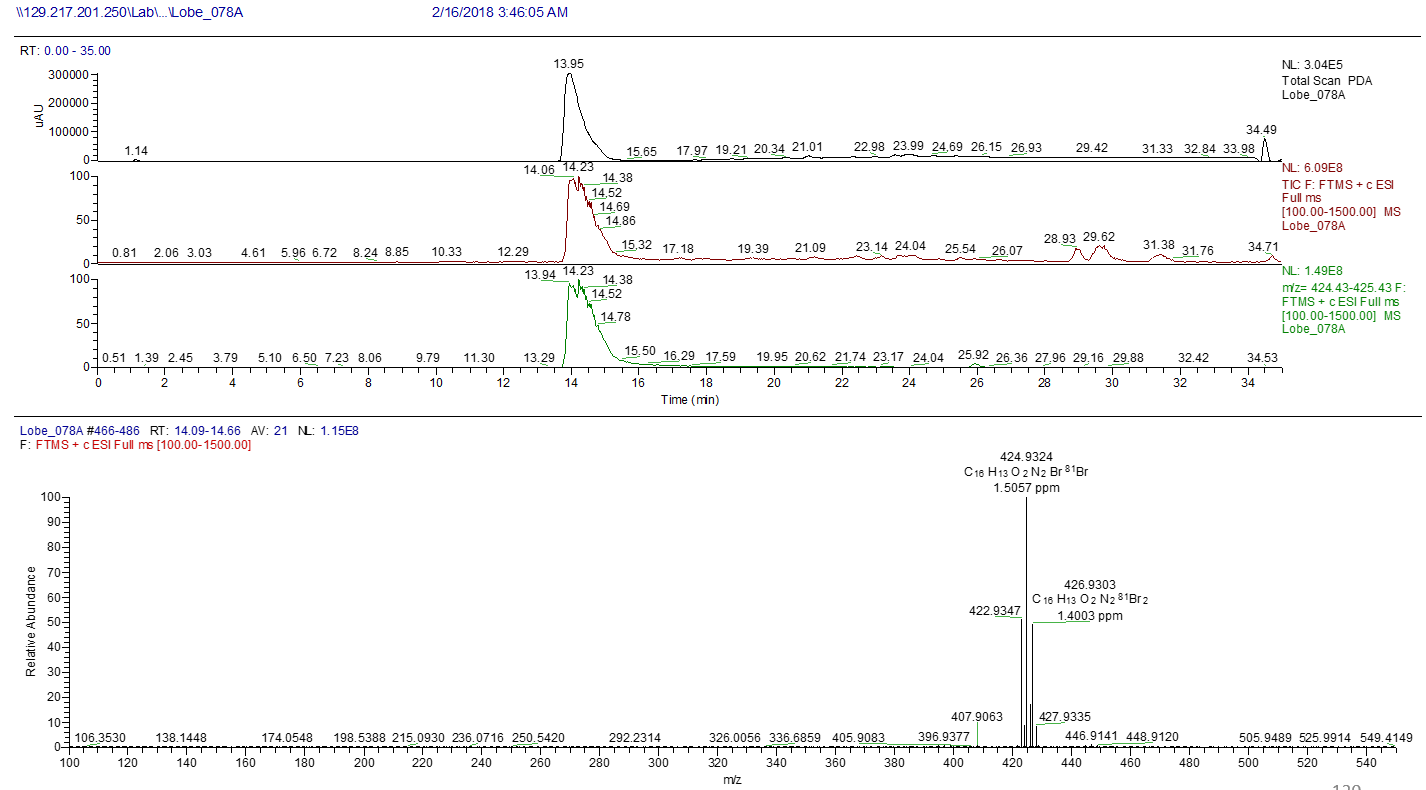


**LC-MS spectra of 5,7-** **Dibromo-8'-hydroxy-3',4'-dihydro-2'H-spiro[indoline-3,1'-isoquinolin]-2-one (1c)**


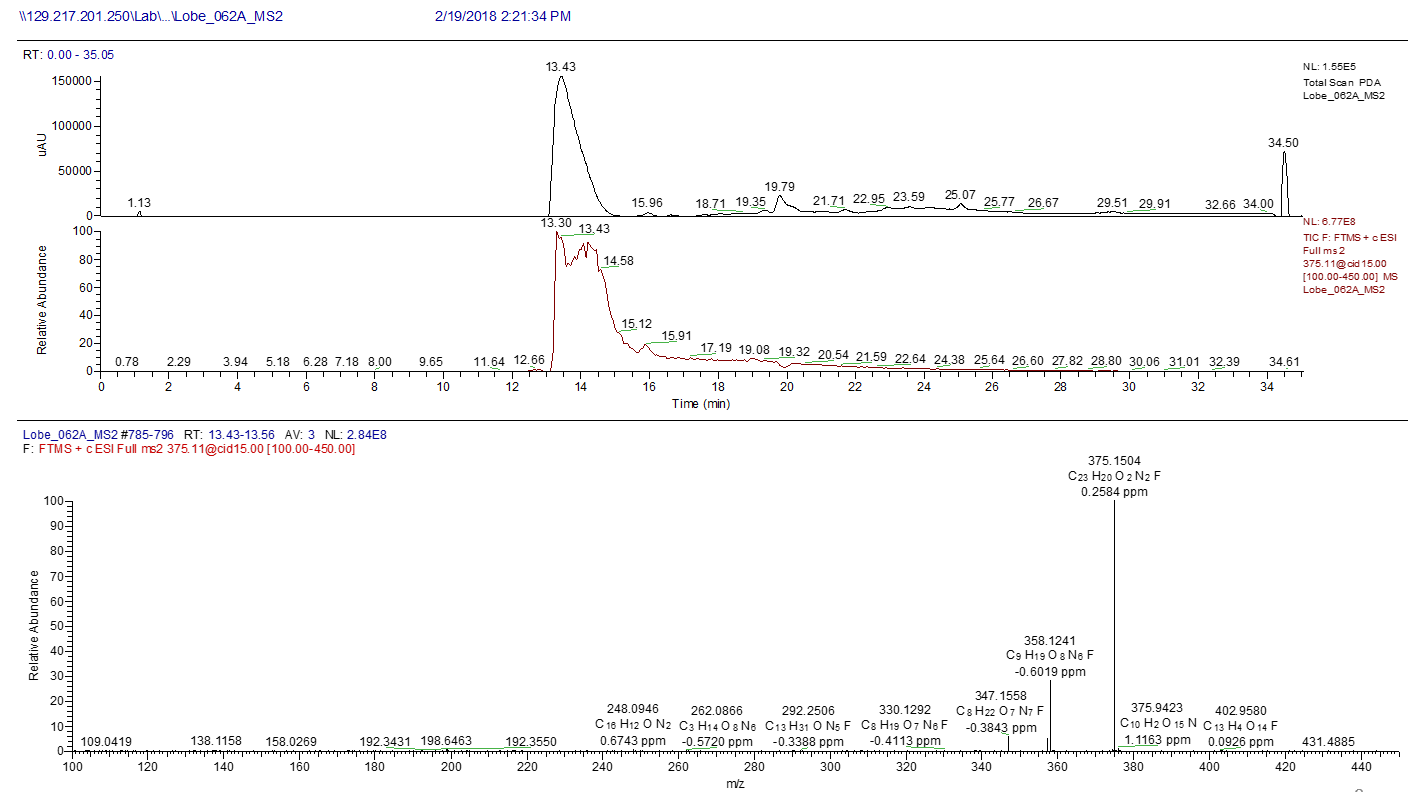


**LC-MS spectra of 1-(4-Fluorobenzyl)-8'-hydroxy-3',4'-dihydro-2'H-spiro[indoline-3,1'-isoquinolin]-2-one (1d)**

**^^**

**LC-MS spectra of 1-(4-Fluorobenzyl)-6'-hydroxy-3',4'-dihydro-2'H-spiro[indoline-3,1'-isoquinolin]-2-one (2d)**

**^^**

**LC-MS spectra of 1-(4-Chlorobenzyl)-8'-hydroxy-3',4'-dihydro-2'H-spiro[indoline-3,1'-isoquinolin]-2-one (1e)**

**^^**

**LC-MS spectra of 1-(4-Chlorobenzyl)-6'-hydroxy-3',4'-dihydro-2'H-spiro[indoline-3,1'-isoquinolin]-2-one (2e)**

**^^**

**LC-MS spectra of 1-(4-bromobenzyl)-8'-hydroxy-3',4'-dihydro-2'H-spiro[indoline-3,1'-isoquinolin]-2-one (1f)**

**^^**

**LC-MS spectra of 1-(4-Bromobenzyl)-6'-hydroxy-3',4'-dihydro-2'H-spiro[indoline-3,1'-isoquinolin]-2-one (2f)**

**^^**

**LC-MS spectra of** **1-(3,4-Dichlorobenzyl)-8'-hydroxy-3',4'-dihydro-2'H-spiro[indoline-3,1'-isoquinolin]-2-one (1g)**

**^^**

**LC-MS spectra of 1-(3,4-Dichlorobenzyl)-6'-hydroxy-3',4'-dihydro-2'H-spiro[indoline-3,1'-isoquinolin]-2-one (2g)**

**^^**


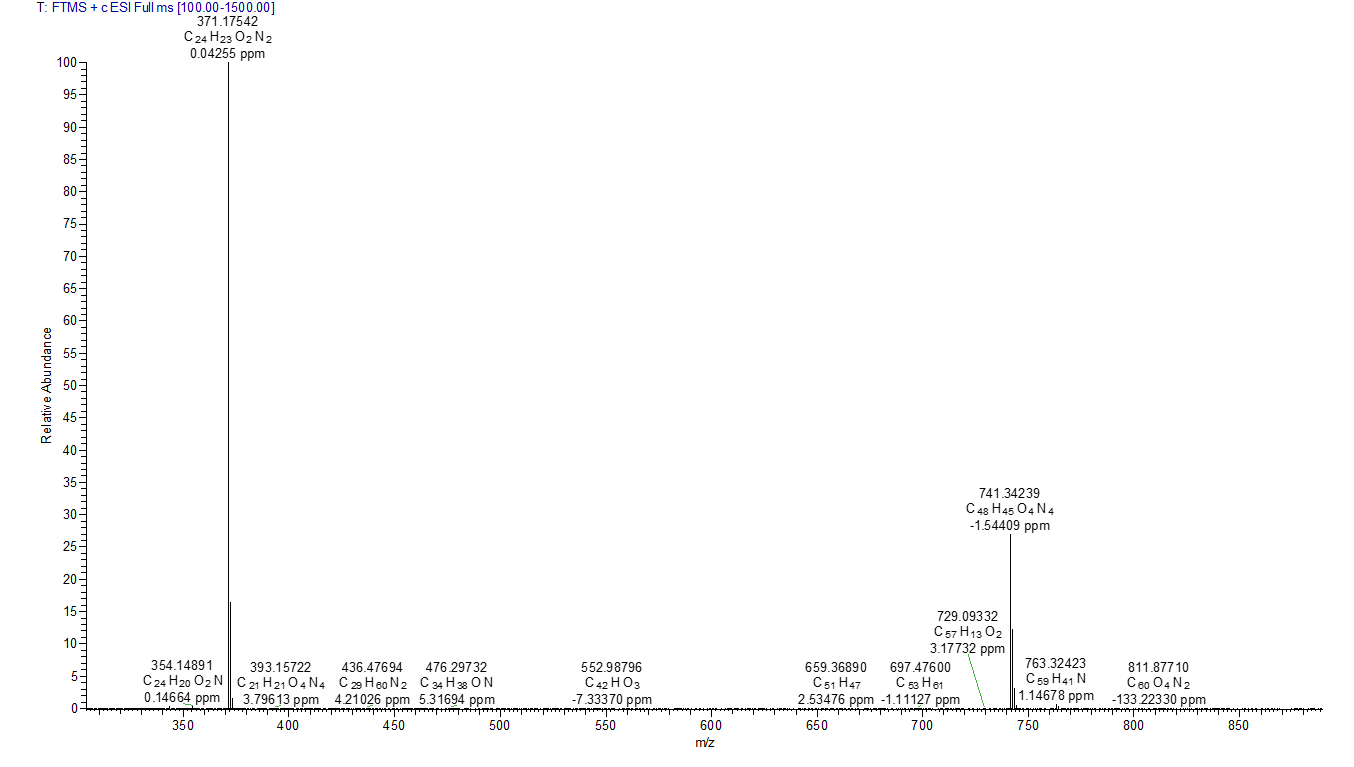


**LC-MS spectra of 8'-Hydroxy-1-(4-methylbenzyl)-3',4'-dihydro-2'H-spiro[indoline-3,1'-isoquinolin]-2-one (1h)**

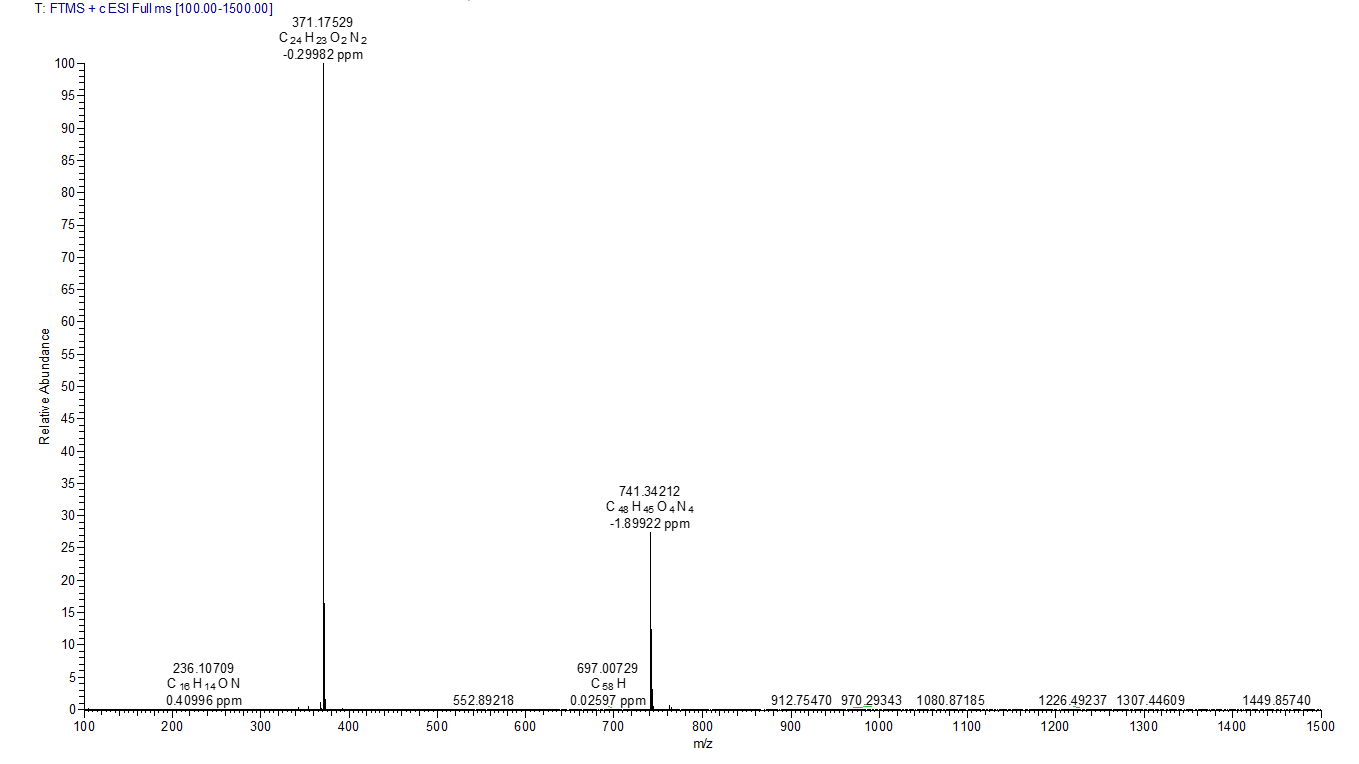


**LC-MS spectra of 6'-Hydroxy-1-(4-methylbenzyl)-3',4'-dihydro-2'H-spiro[indoline-3,1'-isoquinolin]-2-one (2h)**

**LC-MS spectra of 8'-Hydroxy-1-(2-nitrobenzyl)-3',4'-dihydro-2'H-spiro[indoline-3,1'-isoquinolin]-2-one (1i)**

**LC-MS spectra of 6'-Hydroxy-1-(2-nitrobenzyl)-3',4'-dihydro-2'H-spiro[indoline-3,1'-isoquinolin]-2-one (2i)**

**LC-MS spectra of 8’-Hydroxy-1-(naphthalen-2-ylmethyl)-3',4'-dihydro-2'H-spiro[indoline-3,1'-isoquinolin]-2-one (1j)**

**LC-MS spectra of 6’-Hydroxy-1-(naphthalen-2-ylmethyl)-3',4'-dihydro-2'H-spiro[indoline-3,1'-isoquinolin]-2-one (2j)**

**LC-MS spectra of 5-Chloro-1-(4-fluorobenzyl)-8'-hydroxy-3',4'-dihydro-2'H-spiro[indoline-3,1'-isoquinolin]-2-one (1k)**

**LC-MS spectra of 5-Chloro-1-(4-fluorobenzyl)-6'-hydroxy-3',4'-dihydro-2'H-spiro[indoline-3,1'-isoquinolin]-2-one (2k)**

**LC-MS spectra of 5-Chloro-1-(4-chlorobenzyl)-8'-hydroxy-3',4'-dihydro-2'H-spiro[indoline-3,1'-isoquinolin]-2-one (1l)**

**LC-MS spectra of 5-Chloro-1-(4-chlorobenzyl)-6'-hydroxy-3',4'-dihydro-2'H-spiro[indoline-3,1'-isoquinolin]-2-one (2l)**

**LC-MS spectra of 5-Chloro-6'-hydroxy-1-(4-methylbenzyl)-3',4'-dihydro-2'H-spiro[indoline-3,1'-isoquinolin]-2-one (2o)**

**LC-MS spectra of** **6',7'-Dihydroxy-3',4'-dihydro-2'H-spiro[indoline-3,1'-isoquinolin]-2-one (3a)**

**LC-MS spectra of 5-Chloro-6',7'-dihydroxy-3',4'-dihydro-2'H-spiro[indoline-3,1'-isoquinolin]-2-one (3b)**

**LC-MS spectra of**  **6'-Methoxy-3',4'-dihydro-2'H-spiro[indoline-3,1'-isoquinolin]-2-one (4a)**

**LC-MS spectra of** **5-Chloro-6'-methoxy-3',4'-dihydro-2'H-spiro[indoline-3,1'-isoquinolin]-2-one (4b)**

**LC-MS spectra of 5,7-Dibromo-6'-methoxy-3',4'-dihydro-2'H-spiro[indoline-3,1'-isoquinolin]-2-one (4c)**

**LC-MS spectra of 6'-Methoxy-1-(4-fluorobenzyl)-3',4'-dihydro-2'H-spiro[indoline-3,1'-isoquinolin]-2-one (4d)**

**LC-MS spectra of** **6'-Methoxy-1-(4-methylbenzyl)-3',4'-dihydro-2'H-spiro[indoline-3,1'-isoquinolin]-2-one (4e)**

**LC-MS spectra of** **6',7'-Dimethoxy-3',4'-dihydro-2'H-spiro[indoline-3,1'-isoquinolin]-2-one (5a)**

**LC-MS spectra of** **5-Chloro-6',7'-dimethoxy-3',4'-dihydro-2'H-spiro[indoline-3,1'-isoquinolin]-2-one (5b)**

**LC-MS spectra of 5,7-dibromo-6',7'-dimethoxy-3',4'-dihydro-2'H-spiro[indoline-3,1'-isoquinolin]-2-one (5c)**

**LC-MS spectra of 1-(4-Fluorobenzyl)-6',7'-dimethoxy-3',4'-dihydro-2'H-spiro[indoline-3,1'-isoquinolin]-2-one (5d)**

**LC-MS spectra of 6',7'-Dimethoxy-1-(4-methylbenzyl)-3',4'-dihydro-2'H-spiro[indoline-3,1'-isoquinolin]-2-one (5e)**

**LC-MS spectra of 1-(4-Bromobenzyl)-5-chloro-6',7'-dimethoxy-3',4'-dihydro-2'H-spiro[indoline-3,1'-isoquinolin]-2-one (5f)**


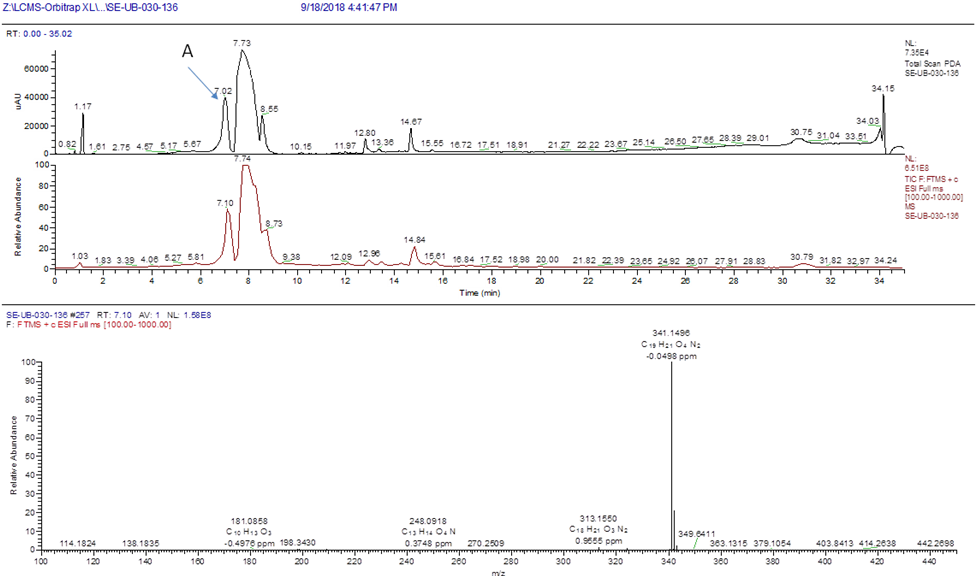


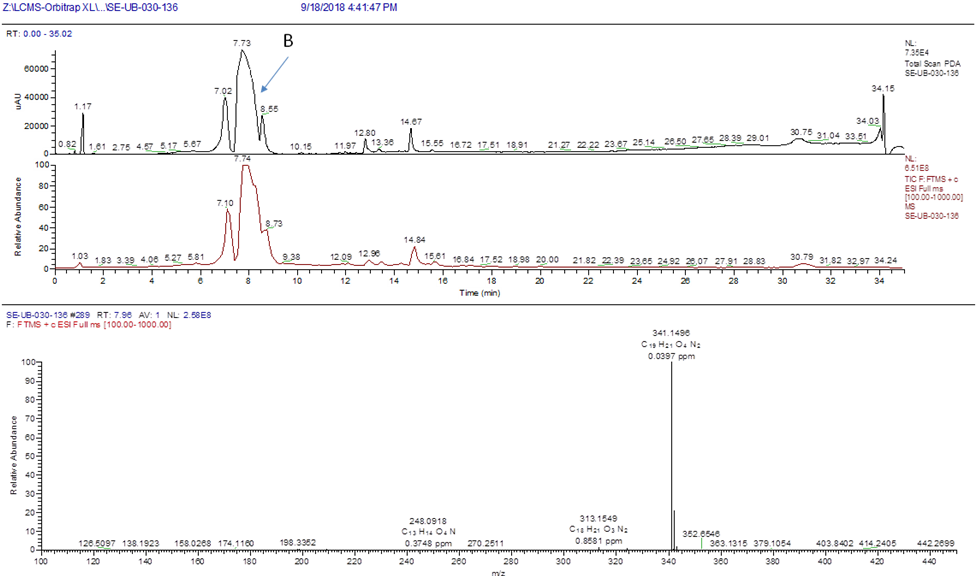


**LC-MS spectra of 6',7',8'-Trimethoxy-3',4'-dihydro-2'H-spiro[indoline-3,1'-isoquinolin]-2-one (6a)**

**LC-MS spectra of 5-Chloro-6',7',8'-trimethoxy-3',4'-dihydro-2'H-spiro[indoline-3,1'-isoquinolin]-2-one (6b)**

**LC-MS spectra of** **5,7-Dibromo-6',7',8'-trimethoxy-3',4'-dihydro-2'H-spiro[indoline-3,1'-isoquinolin]-2-one (6c)**

**LC-MS spectra of 1-(4-Fluorobenzyl)-6',7',8'-trimethoxy-3',4'-dihydro-2'H-spiro[indoline-3,1'-isoquinolin]-2-one (6d)**

**LC-MS spectra of 6',7',8'-Trimethoxy-1-(4-methylbenzyl)-3',4'-dihydro-2'H-spiro[indoline-3,1'-isoquinolin]-2-one (6e)**

Biological Screening Data -Part 1: Percent Growth of Cancer Cell Line (Compounds were tested at 10 µM concentration in the NC1 60 Screen)

| **CANCER TYPE** | **CELL LINE** | **1a** | **1e** | **2b** | **1c** | **2g** | **2h** | **3b** | **4a** | **4b** | **4c** | **4d** |
| --- | --- | --- | --- | --- | --- | --- | --- | --- | --- | --- | --- | --- |
| Leukemia | CCRF-CEM | 101.0 | 93.1 | 98.2 | 89.2 | 89.6 | 73.8 | 114.6 | 106.6 | 104.5 | 113.0 | 101.0 |
| Leukemia | HL-60(TB) | 107.9 | 111.9 | 106.9 | 96.6 | 107.5 | 108.7 | 102.7 | 101.0 | 96.4 | 117.3 | 115.0 |
| Leukemia | K-562 | 99.1 | 86.5 | 103.6 | 91.7 | 81.0 | 89.9 | 78.9 | 104.8 | 98.9 | 98.8 | 96.7 |
| Leukemia | MOLT-4 | 108.4 | 94.9 | 102.4 | 93.7 | 88.0 | 97.0 | 97.6 | 108.5 | 95.5 | 95.9 | 89.1 |
| Leukemia | RPMI-8226 | 103.4 | 90.9 | 103.3 | 86.1 | 84.4 | 94.4 | 78.2 | 104.9 | 100.3 | 105.6 | 95.4 |
| Leukemia | SR | 109.3 | 100.6 | 106.9 | 80.6 | 89.1 | 95.2 | 88.3 | 95.4 | 86.1 | 96.4 | 93.7 |
| Non-Small Cell Lung Cancer | A549/ATCC | 107.7 | 93.4 | 100.0 | 87.6 | 94.9 | 95.0 | 109.0 | 98.1 | 97.1 | 99.2 | 100.4 |
| Non-Small Cell Lung Cancer | EKVX | 104.1 | 91.9 | 93.7 | 75.6 | 86.9 | 98.7 | 84.9 | 90.7 | 94.2 | 78.7 | 87.2 |
| Non-Small Cell Lung Cancer | HOP-62 | 105.3 | 91.5 | 93.5 | 76.5 | 94.5 | 100.7 | 94.2 | 95.4 | 86.7 | 86.1 | 97.7 |
| Non-Small Cell Lung Cancer | HOP-92 | 84.4 | **69.9** | 82.7 | 72.2 | 74.7 | **68.0** | 120.5 | 95.6 | 94.7 | 93.2 | 98.0 |
| Non-Small Cell Lung Cancer | NCI-H226 | 105.6 | 91.9 | 99.8 | 78.1 | 91.1 | 99.4 | 102.8 | 88.4 | 87.2 | 100.8 | 95.4 |
| Non-Small Cell Lung Cancer | NCI-H23 | 98.9 | 94.6 | 97.5 | 87.1 | 92.6 | 98.0 | 90.7 | 97.5 | 93.1 | 98.4 | 105.1 |
| Non-Small Cell Lung Cancer | NCI-H322M | 101.2 | 89.0 | 94.4 | 91.4 | 90.3 | 93.7 | 92.7 | 99.7 | 106.9 | 90.3 | 90.4 |
| Non-Small Cell Lung Cancer | NCI-H460 | 110.5 | 100.7 | 99.5 | 90.9 | 102.7 | 102.9 | 95.8 | 101.1 | 103.1 | 101.5 | 99.3 |
| Non-Small Cell Lung Cancer | NCI-H522 | 102.8 | 94.0 | 94.3 | 87.6 | 94.9 | 94.9 | 84.9 | 96.6 | 95.3 | 91.6 | 91.9 |
| Colon Cancer | COLO 205 | 111.2 | 109.1 | 112.5 | 103.2 | 103.5 | 113.0 | 107.8 | 126.3 | 120.2 | 112.1 | 114.7 |
| Colon Cancer | HCC-2998 | 108.1 | 109.8 | 111.4 | 101.0 | 109.2 | 104.0 | 104.0 | 106.2 | 109.5 | 104.7 | 102.4 |
| Colon Cancer | HCT-116 | 108.9 | 90.6 | 97.9 | 90.3 | 83.5 | 100.1 | 88.6 | 97.9 | 98.5 | 103.2 | 93.1 |
| Colon Cancer | HCT-15 | 112.8 | 97.7 | 95.9 | 100.7 | 97.4 | 97.5 | 79.2 | 98.0 | 99.3 | 104.2 | 102.2 |
| Colon Cancer | HT29 | 109.2 | 98.1 | 101.4 | 99.6 | 103.8 | 102.0 | 115.8 | 109.4 | 109.4 | 103.7 | 105.4 |
| Colon Cancer | KM12 | 105.8 | 100.7 | 104.6 | 82.4 | 95.5 | 100.8 | 111.4 | 96.0 | 98.0 | 104.3 | 101.6 |
| Colon Cancer | SW-620 | 99.2 | 98.1 | 99.4 | 94.6 | 98.1 | 105.7 | 92.1 | 97.2 | 96.6 | 97.4 | 101.0 |
| CNS Cancer | SF-268 | 97.7 | 92.1 | 94.9 | 84.1 | 88.7 | 99.5 | 103.7 | 96.0 | 94.1 | 100.1 | 96.6 |
| CNS Cancer | SF-295 | 108.7 | 102.6 | 100.8 | 92.2 | 94.4 | 95.1 | 103.9 | 94.1 | 96.3 | 102.6 | 100.6 |
| CNS Cancer | SF-539 | 97.6 | 92.7 | 91.6 | 90.2 | 88.3 | 97.1 | 89.1 | 105.4 | 90.2 | 94.0 | 95.0 |
| CNS Cancer | SNB-19 | 99.0 | 86.9 | 93.9 | 93.5 | 89.3 | 100.6 | 97.2 | 96.8 | 99.7 | 91.5 | 85.3 |
| CNS Cancer | SNB-75 | 95.8 | 82.1 | 97.8 | 81.7 | 86.6 | 88.5 | 72.3 | 81.8 | 91.4 | **70.6** | 91.4 |
| CNS Cancer | U251 | 102.1 | 96.5 | 96.4 | 81.7 | 92.3 | 97.4 | 101.1 | 97.1 | 94.1 | 96.7 | 99.4 |
| Melanoma | LOX IMVI | 100.2 | 93.2 | 97.3 | 87.0 | 98.5 | 96.1 | 88.5 | 99.8 | 96.2 | 102.5 | 101.6 |
| Melanoma | MALME-3M | 103.6 | 86.7 | 98.6 | 91.0 | 97.9 | 93.2 | 90.3 | 100.2 | 107.9 | 88.3 | 91.7 |
| Melanoma | M14 | 110.9 | 96.4 | 105.8 | 93.7 | 98.9 | 105.3 | 102.4 | 98.2 | 100.6 | 97.7 | 99.7 |
| Melanoma | MDA-MB-435 | 105.3 | 99.1 | 104.2 | 99.6 | 96.8 | 98.9 | 105.6 | 100.4 | 102.0 | 101.3 | 110.4 |
| Melanoma | SK-MEL-2 | 118.9 | 112.1 | 111.4 | 99.1 | 114.7 | 120.0 | 102.6 | 106.5 | 105.8 | 105.8 | 109.7 |
| Melanoma | SK-MEL-28 | 108.9 | 106.9 | 106.8 | 99.0 | 103.2 | 103.0 | 102.3 | 108.0 | 106.4 | 100.9 | 98.3 |
| Melanoma | SK-MEL-5 | 106.3 | 97.7 | 103.9 | 93.5 | 94.1 | 98.5 | 94.2 | 101.1 | 100.9 | 98.4 | 96.1 |
| Melanoma | UACC-257 | 104.7 | 98.7 | 104.0 | 93.6 | 94.7 | 100.8 | 106.7 | 102.1 | 101.3 | 100.3 | 102.2 |
| Melanoma | UACC-62 | 99.0 | 85.0 | 92.8 | 76.9 | 82.4 | 89.3 | 96.2 | 92.4 | 88.5 | 86.6 | 83.8 |
| Ovarian Cancer | IGROV1 | 104.4 | 97.6 | 103.9 | **62.8** | 102.4 | 98.7 | 85.0 | 89.8 | 90.4 | 82.4 | 82.3 |
| Ovarian Cancer | OVCAR-3 | 105.2 | 97.5 | 101.9 | 85.9 | 95.9 | 105.2 | 81.7 | 102.9 | 99.8 | 101.1 | 101.4 |
| Ovarian Cancer | OVCAR-4 | 101.4 | 86.1 | 104.2 | 84.7 | 93.0 | 95.4 | 91.3 | 99.9 | 105.0 | 95.2 | 111.1 |
| Ovarian Cancer | OVCAR-5 | 100.4 | 101.4 | 99.1 | 98.8 | 93.8 | 99.6 | 95.5 | 103.7 | 104.6 | 91.3 | 86.8 |
| Ovarian Cancer | OVCAR-8 | 104.9 | 106.2 | 107.0 | 93.2 | 103.6 | 105.7 | 95.9 | 98.0 | 97.7 | 101.1 | 101.9 |
| Ovarian Cancer | NCI/ADR-RES | 104.5 | 101.9 | 108.2 | 100.5 | 98.0 | 103.1 | 102.4 | 102.1 | 105.1 | 104.4 | 103.5 |
| Ovarian Cancer | SK-OV-3 | 90.0 | 105.4 | 100.3 | 75.8 | 100.4 | 108.0 | 92.9 | 97.8 | 95.3 | 84.2 | 107.2 |
| Renal Cancer | 786-0 | 105.0 | 97.5 | 95.8 | 88.4 | 88.7 | 106.1 | 106.0 | 99.3 | 103.1 | 94.8 | 99.9 |
| Renal Cancer | A498 | 106.0 | 84.7 | 92.9 | 87.5 | 91.7 | 96.6 | **49.8** | 79.1 | **70.4** | **67.0** | **47.1** |
| Renal Cancer | ACHN | 101.4 | 91.2 | 97.5 | 83.7 | 97.3 | 101.6 | 90.2 | 101.7 | 87.5 | 93.7 | 97.7 |
| Renal Cancer | CAKI-1 | 93.3 | 80.0 | 90.5 | **64.5** | 78.2 | 83.9 | 92.3 | 78.4 | 76.2 | 82.8 | 82.0 |
| Renal Cancer | RXF 393 | 109.1 | 113.5 | 109.3 | 98.9 | 99.0 | 103.0 | 105.5 | 101.6 | 107.3 | 133.0 | 108.0 |
| Renal Cancer | SN12C | 101.8 | 96.6 | 95.6 | 93.4 | 94.8 | 106.1 | 94.5 | 99.3 | 91.7 | 92.5 | 91.0 |
| Renal Cancer | TK-10 | 114.5 | 107.0 | 107.5 | 110.8 | 106.5 | 107.8 | 105.4 | 105.1 | 107.0 | 105.7 | 99.7 |
| Renal Cancer | UO-31 | 87.8 | **64.8** | 83.2 | **64.7** | **67.6** | 75.0 | 71.1 | 81.5 | 77.6 | **62.1** | **58.1** |
| Prostate Cancer | PC-3 | 99.6 | 86.3 | 94.4 | 83.8 | 81.2 | 94.5 | 73.7 | 96.3 | 94.7 | 97.9 | 82.6 |
| Prostate Cancer | DU-145 | 105.7 | 101.1 | 104.9 | 98.1 | 101.9 | 106.3 | 110.8 | 104.4 | 104.4 | 110.2 | 111.8 |
| Breast Cancer | MCF7 | 100.4 | 104.1 | 97.8 | 89.7 | 112.6 | 115.9 | 74.3 | 88.8 | 95.2 | 100.8 | 107.5 |
| Breast Cancer | MDA-MB-231/ATCC | 97.4 | 88.4 | 92.5 | 77.7 | 87.2 | 101.3 | 88.1 | 91.1 | 98.6 | 74.7 | 76.1 |
| Breast Cancer | HS 578T | 105.1 | 91.0 | 99.8 | 82.9 | 94.4 | 104.0 | 92.3 | 92.5 | 88.2 | 94.8 | 96.2 |
| Breast Cancer | BT-549 | 108.7 | 94.8 | 110.1 | 114.3 | 98.6 | 108.8 | 104.4 | 104.8 | 125.4 | 102.5 | 95.5 |
| Breast Cancer | T-47D | 111.0 | 92.2 | 107.0 | 78.0 | 94.0 | 101.8 | 96.8 | 96.6 | 98.4 | 90.3 | 96.7 |
| Breast Cancer | MDA-MB-468 | 97.7 | 106.8 | 108.6 | 100.4 | 99.7 | 110.1 | 123.1 | 93.8 | 100.3 | 137.4 | 113.3 |

Biological Screening Data - Part 2: Percent Growth of Cancer Cell Line (Compounds were tested at 10 µM concentration in the NC1 60 Screen)

| **CANCER TYPE** | **CELL LINE** | **4e** | **5a** | **5b** | **5c** | **5d** | **5e** | **5f** | **6a** | **6b** | **6c** | **6d** | **6e** |
| --- | --- | --- | --- | --- | --- | --- | --- | --- | --- | --- | --- | --- | --- |
| Leukemia | CCRF-CEM | 93.7 | 94.4 | 98.5 | 85.1 | 106.5 | 96.2 | 84.4 | 87.0 | 93.8 | 104.4 | 103.8 | 107.6 |
| Leukemia | HL-60(TB) | 98.5 | 101.3 | 100.2 | 100.1 | 90.4 | 98.3 | 77.9 | 95.3 | 99.6 | 127.5 | 96.8 | 113.2 |
| Leukemia | K-562 | 89.5 | 95.6 | 89.1 | 95.3 | 87.7 | 75.8 | **55.4** | 92.1 | 100.6 | 106.0 | 94.4 | 96.5 |
| Leukemia | MOLT-4 | 87.4 | 107.3 | 91.0 | 94.8 | 95.5 | 95.7 | **57.0** | 77.6 | 90.4 | 91.8 | 85.3 | 92.4 |
| Leukemia | RPMI-8226 | 95.1 | 93.6 | 99.2 | 84.3 | **65.4** | **52.2** | **46.3** | 94.1 | 94.0 | 88.3 | 85.7 | 92.0 |
| Leukemia | SR | 85.9 | 94.2 | 80.2 | 76.3 | 92.4 | 74.4 | **62.3** | 82.8 | 91.9 | 86.0 | 96.3 | 100.7 |
| Non-Small Cell Lung Cancer | A549/ATCC | 86.4 | 97.2 | 94.0 | 91.3 | 105.8 | 87.4 | 82.4 | 87.8 | 89.8 | 95.5 | 109.4 | 92.5 |
| Non-Small Cell Lung Cancer | EKVX | 72.0 | 91.9 | 93.5 | 89.1 | 97.4 | 97.9 | 86.9 | 86.5 | 79.5 | 84.6 | 98.4 | 92.7 |
| Non-Small Cell Lung Cancer | HOP-62 | 82.8 | 89.8 | 87.4 | 88.9 | 94.2 | 93.3 | 90.7 | 92.5 | 88.9 | 88.5 | 103.0 | 98.4 |
| Non-Small Cell Lung Cancer | HOP-92 | 83.3 | 83.5 | 80.3 | 82.6 | 91.4 | 92.0 | 86.6 | 80.6 | 78.8 | 95.9 | 94.3 | 106.2 |
| Non-Small Cell Lung Cancer | NCI-H226 | 76.5 | 94.7 | 91.0 | 108.6 | 94.5 | 79.8 | 74.3 | 99.7 | 87.1 | 94.9 | 93.6 | 91.0 |
| Non-Small Cell Lung Cancer | NCI-H23 | 89.6 | 93.7 | 90.0 | 88.8 | 96.2 | 85.9 | 80.2 | 106.7 | 99.5 | 95.5 | 99.2 | 107.5 |
| Non-Small Cell Lung Cancer | NCI-H322M | 87.9 | 100.9 | 104.8 | 98.0 | 91.9 | 93.5 | 99.9 | 93.2 | 91.6 | 94.3 | 96.4 | 90.7 |
| Non-Small Cell Lung Cancer | NCI-H460 | 94.7 | 101.9 | 98.1 | 96.4 | 98.5 | 89.2 | 83.7 | 79.7 | 82.9 | 99.9 | 100.3 | 102.0 |
| Non-Small Cell Lung Cancer | NCI-H522 | 88.5 | 93.4 | 95.0 | 83.9 | 83.2 | 83.9 | 73.5 | 83.4 | 88.8 | 84.9 | 91.8 | 80.6 |
| Colon Cancer | COLO 205 | 122.8 | 111.5 | 115.0 | 112.4 | 100.7 | 118.6 | 98.9 | 116.6 | 105.0 | 110.9 | 111.4 | 112.9 |
| Colon Cancer | HCC-2998 | 105.9 | 105.2 | 97.8 | 104.2 | 97.6 | 102.9 | 93.0 | 110.1 | 119.3 | 105.6 | 106.6 | 105.6 |
| Colon Cancer | HCT-116 | 85.9 | 98.4 | 96.3 | 94.3 | 91.6 | 92.4 | **68.5** | 98.2 | 94.0 | 102.9 | 91.2 | 97.1 |
| Colon Cancer | HCT-15 | 97.6 | 102.9 | 93.4 | 92.5 | 94.6 | 95.9 | 74.0 | 100.6 | 98.4 | 104.8 | 109.5 | 108.0 |
| Colon Cancer | HT29 | 97.8 | 108.7 | 108.1 | 96.1 | 106.0 | 98.2 | 96.3 | 94.5 | 96.4 | 104.4 | 107.6 | 102.1 |
| Colon Cancer | KM12 | 95.7 | 93.8 | 94.6 | 92.6 | 102.7 | 92.7 | 83.3 | 94.6 | 92.1 | 101.7 | 105.4 | 103.6 |
| Colon Cancer | SW-620 | 92.4 | 91.5 | 95.4 | 96.8 | 105.9 | 92.5 | 90.7 | 91.7 | 95.2 | 98.9 | 101.4 | 103.1 |
| CNS Cancer | SF-268 | 87.8 | 94.4 | 90.4 | 88.1 | 96.2 | 91.5 | 83.4 | 97.4 | 93.0 | 95.0 | 95.6 | 98.0 |
| CNS Cancer | SF-295 | 96.5 | 100.7 | 97.6 | 101.7 | 100.4 | 101.1 | 93.9 | 101.7 | 101.9 | 106.6 | 107.0 | 102.5 |
| CNS Cancer | SF-539 | 97.5 | 95.0 | 90.8 | 89.0 | 99.2 | 95.8 | 103.9 | 92.9 | 90.5 | 93.8 | 100.0 | 102.5 |
| CNS Cancer | SNB-19 | 92.9 | 98.7 | 101.9 | 102.2 | 93.6 | 95.9 | 92.9 | 94.5 | 94.2 | 90.5 | 87.5 | 91.4 |
| CNS Cancer | SNB-75 | 89.1 | 95.4 | **68.9** | 75.0 | 72.6 | 100.5 | 79.4 | 108.1 | 88.2 | 71.1 | 89.0 | 88.0 |
| CNS Cancer | U251 | 89.6 | 89.5 | 88.1 | 92.1 | 105.4 | 94.0 | 84.1 | 88.2 | 87.2 | 99.2 | 109.3 | 100.2 |
| Melanoma | LOX IMVI | 93.5 | 94.5 | 89.2 | 93.6 | 99.3 | 98.6 | 85.6 | 92.4 | 93.7 | 93.9 | 99.1 | 97.4 |
| Melanoma | MALME-3M | 93.4 | 92.8 | 103.5 | 101.1 | 78.7 | 88.0 | 91.1 | 89.1 | 92.8 | 85.1 | 83.2 | 87.0 |
| Melanoma | M14 | 95.2 | 106.3 | 102.2 | 104.3 | 97.1 | 98.1 | 89.8 | 95.5 | 91.4 | 101.3 | 99.3 | 92.7 |
| Melanoma | MDA-MB-435 | 103.1 | 103.7 | 101.4 | 98.0 | 96.3 | 98.4 | 90.0 | 95.6 | 93.0 | 102.7 | 100.5 | 99.3 |
| Melanoma | SK-MEL-2 | 100.5 | 107.8 | 103.3 | 104.9 | 107.2 | 109.2 | 100.4 | 98.7 | 100.1 | 106.6 | 113.2 | 105.0 |
| Melanoma | SK-MEL-28 | 104.3 | 104.8 | 109.0 | 111.1 | 98.5 | 100.0 | 98.6 | 96.9 | 93.8 | 97.9 | 99.7 | 103.4 |
| Melanoma | SK-MEL-5 | 98.2 | 99.1 | 91.7 | 104.5 | 87.5 | 75.3 | **56.7** | 100.0 | 91.8 | 93.5 | 92.2 | 93.4 |
| Melanoma | UACC-257 | 99.2 | 95.8 | 98.0 | 102.7 | 99.5 | 90.6 | 74.4 | 83.6 | 83.6 | 102.2 | 109.4 | 97.8 |
| Melanoma | UACC-62 | 84.1 | 94.3 | 91.4 | 87.3 | **69.7** | **66.5** | **55.0** | 92.5 | 78.5 | 73.4 | 72.5 | 78.5 |
| Ovarian Cancer | IGROV1 | 85.9 | 83.4 | 91.4 | 105.0 | 82.8 | 79.6 | 90.2 | 87.2 | 76.9 | 74.5 | 89.5 | 89.4 |
| Ovarian Cancer | OVCAR-3 | 91.6 | 95.6 | 99.1 | 99.4 | 95.1 | 88.3 | 76.3 | 101.7 | 91.0 | 101.6 | 103.3 | 103.7 |
| Ovarian Cancer | OVCAR-4 | 94.2 | 105.4 | 95.2 | 93.0 | 85.3 | 97.8 | **60.7** | 86.7 | 80.1 | 100.4 | 93.3 | 98.3 |
| Ovarian Cancer | OVCAR-5 | 80.2 | 98.8 | 105.1 | 100.1 | 91.8 | 93.9 | 98.7 | 99.7 | 100.3 | 92.5 | 102.7 | 102.0 |
| Ovarian Cancer | OVCAR-8 | 93.9 | 98.5 | 95.5 | 95.5 | 107.7 | 98.0 | 87.1 | 93.6 | 91.7 | 98.4 | 107.4 | 97.0 |
| Ovarian Cancer | NCI/ADR-RES | 92.4 | 101.5 | 95.7 | 100.7 | 99.5 | 98.8 | 82.0 | 93.2 | 97.9 | 102.6 | 101.5 | 100.8 |
| Ovarian Cancer | SK-OV-3 | 90.6 | 87.7 | 81.4 | 89.9 | 102.4 | 88.8 | 82.9 | 180.2 | 164.9 | 90.4 | 104.4 | 106.8 |
| Renal Cancer | 786-0 | 84.7 | 95.0 | 92.2 | 94.4 | 98.5 | 95.3 | 89.1 | 100.1 | 98.8 | 96.3 | 105.5 | 101.0 |
| Renal Cancer | A498 | **53.9** | **63.3** | 78.9 | **64.3** | 88.9 | 82.8 | 80.1 | 79.5 | 88.9 | 93.9 | 74.3 | 91.7 |
| Renal Cancer | ACHN | 91.4 | 97.6 | 95.6 | 103.0 | 99.3 | 93.6 | 89.1 | 92.4 | 86.8 | 86.6 | 97.1 | 99.6 |
| Renal Cancer | CAKI-1 | **61.0** | 83.4 | 77.9 | 73.2 | 76.3 | 75.0 | 69.9 | 93.3 | 71.5 | 74.8 | 86.9 | 83.5 |
| Renal Cancer | RXF 393 | 93.8 | 108.2 | 102.0 | 100.6 | 99.6 | 88.0 | 99.7 | 109.3 | 100.3 | 108.8 | 104.4 | 100.8 |
| Renal Cancer | SN12C | 88.7 | 96.1 | 97.5 | 91.3 | 92.1 | 91.9 | 87.2 | 99.6 | 94.5 | 93.2 | 90.8 | 91.9 |
| Renal Cancer | TK-10 | 105.5 | 98.9 | 100.5 | 99.5 | 110.7 | 110.5 | 102.6 | 92.0 | 109.8 | 103.7 | 113.0 | 105.6 |
| Renal Cancer | UO-31 | 70.2 | 75.6 | 91.1 | 79.9 | **63.4** | 74.8 | 84.0 | 78.1 | **68.5** | **50.6** | **64.2** | **64.1** |
| Prostate Cancer | PC-3 | 88.4 | 94.6 | 96.0 | 94.0 | **66.2** | **57.8** | **47.9** | 86.4 | 81.2 | 78.3 | 78.5 | 82.4 |
| Prostate Cancer | DU-145 | 105.2 | 103.4 | 101.7 | 102.3 | 106.1 | 105.9 | 96.6 | 101.8 | 96.1 | 108.5 | 108.6 | 106.5 |
| Breast Cancer | MCF7 | 84.6 | 89.1 | 84.3 | 85.2 | 97.8 | 83.1 | 83.7 | 92.7 | 93.1 | 88.7 | 110.3 | 103.1 |
| Breast Cancer | MDA-MB-231/ATCC | 75.4 | 93.4 | 90.8 | 101.5 | 84.9 | 76.9 | 80.3 | 93.5 | 72.3 | 73.3 | 84.3 | 92.7 |
| Breast Cancer | HS 578T | 70.6 | 83.4 | 92.1 | 86.8 | 89.3 | 80.8 | 87.7 | 85.9 | 87.2 | 82.1 | 93.0 | 95.1 |
| Breast Cancer | BT-549 | 115.7 | 124.8 | 118.2 | 127.4 | 92.2 | 106.6 | 103.6 | 121.4 | 112.7 | 105.8 | 103.5 | 103.4 |
| Breast Cancer | T-47D | 85.4 | 90.7 | 83.5 | 93.5 | 76.2 | 78.3 | 73.1 | 93.5 | 84.4 | 92.8 | 93.7 | 83.6 |
| Breast Cancer | MDA-MB-468 | 93.8 | 94.1 | 95.7 | 107.8 | 93.0 | 84.3 | **64.3** | 98.7 | 98.7 | 105.8 | 92.1 | 88.0 |

The compounds 1d, 2d, 2e, 1f, 2f, 1g, 1h, 1i, 2i, 1j, 2j, 1k, 2k, 1l, 2l, 2o and 3a were not tested.
